# Mitogenomic data indicate admixture components of Central-Inner Asian and Srubnaya origin in the conquering Hungarians

**DOI:** 10.1371/journal.pone.0205920

**Published:** 2018-10-18

**Authors:** Endre Neparáczki, Zoltán Maróti, Tibor Kalmár, Klaudia Kocsy, Kitti Maár, Péter Bihari, István Nagy, Erzsébet Fóthi, Ildikó Pap, Ágnes Kustár, György Pálfi, István Raskó, Albert Zink, Tibor Török

**Affiliations:** 1 Department of Genetics, University of Szeged, Szeged, Hungary; 2 Department of Pediatrics and Pediatric Health Center, University of Szeged, Szeged, Hungary; 3 SeqOmics Biotechnology Ltd., Mórahalom, Hungary; 4 Institute of Biochemistry, Biological Research Centre, Szeged, Hungary; 5 Department of Anthropology, Hungarian Natural History Museum, Budapest, Hungary; 6 Department of Biological Anthropology, University of Szeged, Szeged, Hungary; 7 Institute of Genetics, Biological Research Centre, Szeged, Hungary; 8 Institute for Mummies and the Iceman, EURAC, Bolzano, Italy; University of Florence, ITALY

## Abstract

It has been widely accepted that the Finno-Ugric Hungarian language, originated from proto Uralic people, was brought into the Carpathian Basin by the conquering Hungarians. From the middle of the 19^th^ century this view prevailed against the deep-rooted Hungarian Hun tradition, maintained in folk memory as well as in Hungarian and foreign written medieval sources, which claimed that Hungarians were kinsfolk of the Huns. In order to shed light on the genetic origin of the Conquerors we sequenced 102 mitogenomes from early Conqueror cemeteries and compared them to sequences of all available databases. We applied novel population genetic algorithms, named Shared Haplogroup Distance and MITOMIX, to reveal past admixture of maternal lineages. Our results show that the Conquerors assembled from various nomadic groups of the Eurasian steppe. Population genetic results indicate that they had closest connection to the Onogur-Bulgar ancestors of Volga Tatars. Phylogenetic results reveal that more than one third of the Conqueror maternal lineages were derived from Central-Inner Asia and their most probable ultimate sources were the Asian Scythians and Asian Huns, giving support to the Hungarian Hun tradition. The rest of the lineages most likely originated from the Bronze Age Potapovka-Poltavka-Srubnaya cultures of the Pontic-Caspian steppe. Available data imply that the Conquerors did not have a major contribution to the gene pool of the Carpathian Basin.

## Introduction

Foundation of the Hungarian state is connected to the conquering Hungarians, which arrived from the Pontic steppes and occupied the Carpathian Basin at 895–905 AD as a confederation of seven tribes under the leadership of prince Árpád. Modern Hungarians are generally identified as successors of the conquering Hungarians (hence shortened as Conquerors). Until the middle of the 19^th^ century it was generally accepted that Hungarians were kinsfolk of the Huns and Scythians, besides Árpád was a direct descendant of the great Hun leader Attila. Hun-Hungarian affinity was declared in Hungarian and foreign written sources and has been maintained in Hungarian folk memory [1–3]. In the second half of the 19th century the Hungarian language was reclassified as belonging to the Uralic branch of the Finno-Ugric language family [4]. Philological arguments launched a reevaluation of previous assumptions and as a result, the credibility of medieval historical sources, including Hun-Hungarian relations, has been questioned. In following decades the conquering Hungarians were deemed descendants of hypothetic proto Uralic people, the putative common ancestors of people belonging to this language family. Lately most philologists proclaim separability of linguistic and genetic relations, but appearance of the Hungarian language in the Carpathian Basin is explicitly linked to the Conquerors [5].

The possible genetic relation of modern Hungarians to Finno-Ugric groups was tested in several studies [6–8], however all these found Hungarians being genetically unrelated to Uralic people. One of the latest studies [9] reported that a Y-chromosome haplogroup (*N-L1034*) is shared between 4% of the Hungarian Seklers (Hungarian-speaking ethnic group living in Transylvania) and 15% of the closest language relatives the Mansis, though the same marker is also present in Central Asian Uzbeks and has been detected just in one Hungarian [10]. These results indicated that Uralic genetic links hardly exist in modern Hungarians.

The genetic composition of the Conquerors was also analyzed in several ancient DNA (aDNA) studies [11–13] and indeed, all these detected significant presence of east Eurasian major mtDNA haplogroups (Hg-s), which are rare in modern Hungarians but are found in Uralic people. Another study [14] showed the presence of *N-Tat* (*M46*) Y-chromosome marker (a major clade of the above mentioned *N-L1034*) in two of the Conqueror samples and one living Sekler, which was interpreted as a Finno-Ugric link. It is notable that in the latest studies [12,13] population genetic analysis also indicated considerable Central Asian affinity of the Conquerors. However in these studies just hypervariable regions (HVR) of the mtDNA were analyzed, and more reliable Next Generation Sequencing (NGS) data [15] have not been available from the Conquerors yet. Entire mitochondrial genome sequences enable a much higher resolution analysis, as most variable sites of mtDNA are located outside HVR [16].

In order to elicit the genetic origin and relationships of the Conquerors, we set out to assemble a full length mtDNA sequence database from the earliest Conqueror cemeteries. Full length mitogenomes are the most informative source of maternal population histories, as some of the subclades have very distinctive geographic distribution [17,18], reviewed in [19]. Thus the availability of ancient mitogenomes obtained with NGS greatly enhanced the resolution of the phylogeographic approach, making it possible to refine the view of peopling of the Americas [20] and Europe [21–23]. We also made use of this approach by comparing the mtDNA genomes of 102 Conqueror individuals to available public databases. Applying phylogenetic analysis we could allocate the presumptive geographical origin of individual Conqueror Hg lineages to distant regions of East and West Eurasia, while population genetic results pointed at source populations in Volga district, today’s Belarus, Tuva and Central Asia, providing new information about the origin of the Conquerors which is reconcilable with historical sources.

## Materials and methods

### Archaeological background

**Fig 1:**
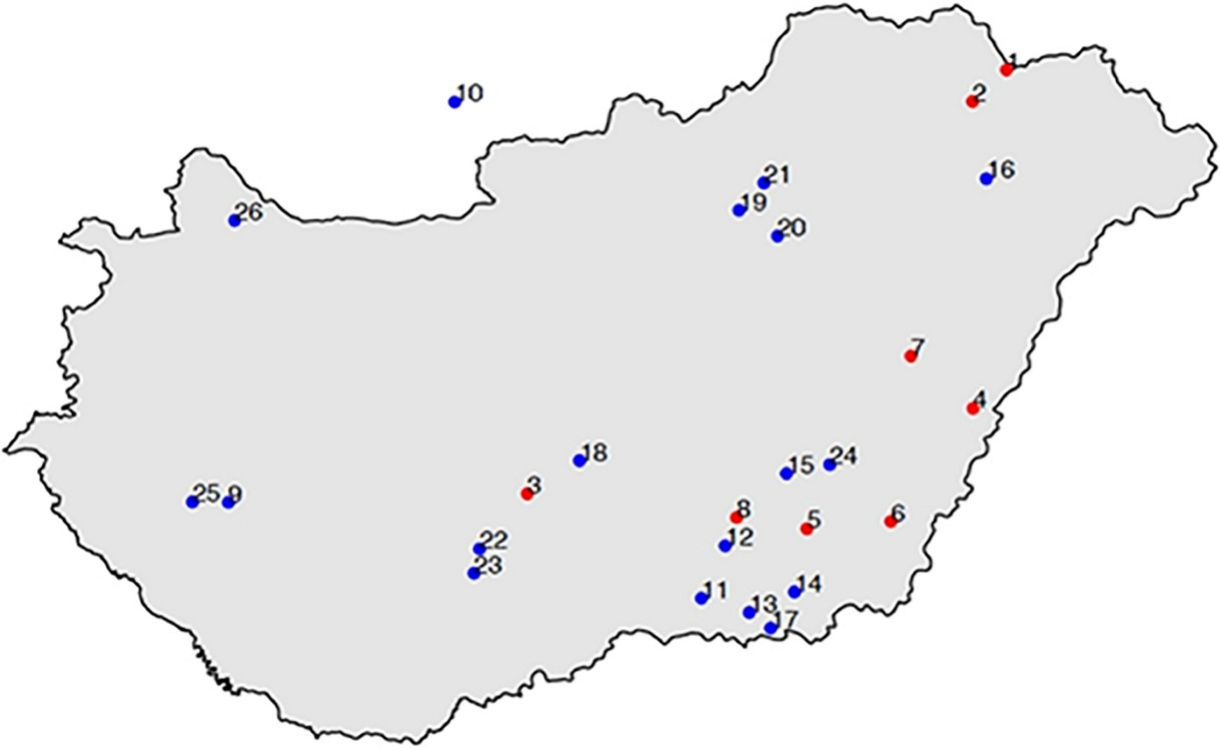
Location of the Hungarian Conqueror cemeteries. Red dots indicate cemeteries reported in this study, blue dots indicate cemeteries from which HVR sequences were reported in [11,12]. Numbers indicate the following sites: 1. Karos-Eperjesszög, 2. Kenézlő-Fazekaszug-II, 3. Harta-Freifelt, 4. Magyarhomoróg, 5. Orosháza-Görbicstanya, 6. Szabadkígyós-Pálliget, 7. Sárrétudvari-Hízóföld, 8. Szegvár-Oromdűlő, 9. Balatonújlak-Erdődűlő, 10. Levice-Géňa, 11. Kiskundorozsma-Hosszúhát, 12. Baks-Iskola, 13. Szeged-Öthalom, 14. M43 no. 25 site Makó-Igási járandó, 15. Szentes-Derekegyháza, 16. Nyíregyháza-Oross Megapark, 17. Kiszombor, 18. Izsák-Balázspuszta, 19. Aldebrő-Mocsáros, 20. Besenyőtelek-Szőrhát, 21. Eger-Szépasszonyvölgy, 22. Fadd-Jegeshegy, 23. Mözs-Szárazdomb, 24. Örménykút, 25. Zalavár-Kápolna, 26. Lébény-Kaszás. Map was created with the maps package of R [24].

In the 10^th^ century a uniform well distinguishable new archaeological culture appeared in the Carpathian Basin which can be connected to the historical record of the conquering Hungarians. We extracted ancient DNA from 102 Conqueror individuals, derived from 8 different cemeteries (Fig 1).

As one of our purposes was to characterize the entire population from a few early Conqueror cemeteries, the majority of samples came from three cemeteries of Karos-Eperjesszög, representing the earliest Conquerors in the Carpathian Basin. These three cemeteries are located in the upper Tisza river region on neighboring sand dunes a few 100 meters from each other, with the richest archaeological findings of the period, and were probably used by contemporary neighboring communities from the last years of the ninth century to the middle of the tenth century, based on dating with coins and comparative analysis of archaeological findings [25]. Basic archaeological description of the cemeteries were given in [13], further details are provided in S1 Appendix and S1A Table.

Specimen numbers are the same as complete repository information, which correspond to the sample names provided in the paper, summarized in S1A Table. Sample geographic locations are provided in S1 Appendix and Fig 1. Name of permit issuing authority for this study:

Department of Anthropology; Hungarian Natural History Museum; Budapest, H-1083; Hungary

Permit number: M-2017-006.

### NGS sequencing

Details of the aDNA purification, hybridization capture, sequencing and sequence analysis methods are given in [15] and were deposited to http://dx.doi.org/10.17504/protocols.io.rmvd466. In order to authenticate the results, we considered the latest recommendations of [26] throughout of the experiments. We tried to apply the modifications recommended by [27] on a few samples (Kenézlő-Fazekaszug/ 1027, 1044, 1045 and 10936, Sárrétudvari-Hízóföld/ 66 and 103), but in our hands this method gave rather varying coverage. In some of the samples (Karos2/2, 17, 18, 33, 44, 67, Karos3/7, 9, 11, 13, 17, 18, Sárrétudvari-Hízóföld/9-anc11, Sárrétudvari-H/81, 136 and Kenézlő-F/1025, 1031, 1036, 1041, 1042) we decreased the recommended USER and UGI concentrations of [28] to half (0.03 U/μL) and at the same time increased the incubation time from 30 to 40 minutes. This modification removed uracils with comparable efficiency to the original method.

Details of NGS data are shown in S2A and S2B Table. Most genomes had satisfactory coverage, but we also included several low coverage sequences, whose Hg-s could be unmistakably classified, as these revealed meaningful maternal relationships within and between cemeteries. Contamination was estimated with two methods; a) using the Schmutzi algorithm and b) calculating the proportion of reads which did not correspond to the consensus sequence in diagnostic positions as in [15], the two methods gave consistent results. The raw nucleotide sequence data of the 102 samples were deposited to the European Nucleotide Archive (http://www.ebi.ac.uk/ena) under accession number PRJEB21279.

### Phylogenetic study

We have downloaded all available modern (n = 32683) and ancient (n = 564, S3C and S3D Table) complete mtDNA genome sequences from the NCBI and European Nucleotide Archive databases or requested them from the authors. This database was also augmented with 314 mitogenomes including 272 new Hungarian ones described in [29]. Then we determined the haplogroups of all sequences with the HaploFind program [30], and arranged them according to haplogroups. Next we selected each subset of sequences (28-180/Hg) corresponding to the Hg of individual Conqueror samples. Selected sequence subsets were aligned with MAFFT version 7 [31,32] using progressive G-INS-1 setting. Aligned multifasta groups were converted into Nexus file with MEGA [33], then Median-Joining networks [34] were drawn with PopART [35]. Finally phylogeographic connections were inferred by looking up the geographic origin of the closest matching samples from the literature (S1 Fig).

### Population genetic study

We have created an Eurasian population database by grouping those mtDNA genomes according to their geographic origin, for which this information was available (S3A Table). Our population database contains 12224 modern samples from 62 Eurasian populations (S3B Table), not considering India and Southeast Asia. In cases when populations were underrepresented we grouped related neighboring groups, like Mansis with Khantys, Belgians with Dutch etc., as listed in S3B Table. We also created a similar mitogenomic population database from 25 ancient Eurasian populations including 496 sequences, though most of these contain low number of samples (S3C Table).

We compared the genetic similarity of populations with two independent methods. We applied the traditional sequence based method calculating pair-wise population differentiation values (Fst) with Arlequin 3.5.2.2 [36] from entire mtDNA genomes (S4A Table) assuming a Tamura & Nei substitution model (Tamura and Nei, 1993) with a gamma value of 0.325. Significant variations in Fst values were tested by 10,000 permutations between populations. As individual insertions and deletions make the alignment of multiple mtDNA genomes troublesome, only variable positions were aligned, and insertions and deletions were recoded to SNP-s as follows. Whole mtDNA genome fasta files were aligned to the NC_012920 human mtDNA reference sequence by an IUPAC code aware in-house aligner using the Needleman–Wunsch algorithm with weight parameters: match 6, IUPAC2match (R, Y, M, W, S, K) 3, IUPAC3match (B, D, H, V) 2, IUPAC4match (N) 1, mismatch -12, gap open -24, gap extend -6. Modern sequences with more than 500 missing or uncertain nucleotides (nt.) were excluded from further analysis. Then all nt. positions where any variation was detected were outputted to VCF files. Since Arlequin cannot manage VCF files SNPs, deletions and insertions were recoded by the following rules: nt-s with no variation at the given position were coded as the reference nt.; SNPs with variation were coded as the alternate allele; all insertions were coded as additional nt. letters, C for samples with reference sequence and T for samples containing the insertion; all deletions were also coded as additional nt. letters, T for samples with reference sequence and C for samples containing the deletion. Then Arlequin input files (arp) were generated from the recoded DNA sequences.

Multidimensional scaling (MDS) was applied on the matrix of linearized Slatkin Fst values [37] and visualized in the two-dimensional space using the cmdscale function implemented in R 3.0.3 [38].

In a second novel approach we also calculated so called Shared Haplogroup Distance (SHD) values between populations [29]. This method considers that all individuals within the same sub-Hg were descended from a single foremother, therefore their maternal lineages are more closely related to each-other than to individuals of neighboring sub-Hg-s. Thus presence of identical terminal subgroups in two populations testifies shared ancestry or past admixture. While Fst based calculations are best suited for measuring evolutionary distances between not admixing populations, we demonstrate that SHD based distance reveals recent admixtures more accurately. We also show that SHD (and MITOMIX see below) results are in accord with Fst calculations. SHD calculations give a distance value between 0–1, which is minimum between populations containing the same sub-haplogroups with identical frequencies, and maximum between populations with no sub-Hg overlap. We used corrected SHD vales, which also takes into account the mutation and fixation rate on the mtDNA genome, thereby allows some connection between progenitor and progeny Hg lineages [29]. Pair-wise SHD distances were calculated between all 87 ancient and modern populations from the frequency of 1942 sub-Hg-s occurring in any of them (S4B Table).

As an additional benefit, SHD enables a hypothesis independent computation to reveal plausible past admixture events. Thus we have also introduced another novel algorithm called MITOMIX, which computes all possible combinations and proportions of *K* populations to find the best fitting admixtures with the smallest SHD values from a test population [29]. In our experience most test populations are adequately admixed from 3–6 other populations, as *K* values greater than 6 do not significantly improve the result. Theoretically MITOMIX can accurately reconstruct past population admixtures if representative data are available from all periods and locations, but it allows meaningful insights even from limited data [29]. With this method we have calculated the best population admixtures giving the most similar mitogenome composition to that of the Conquerors, as well as for their possible source populations (S5 Table).

### Craniofacial reconstructions

**Fig 2:**
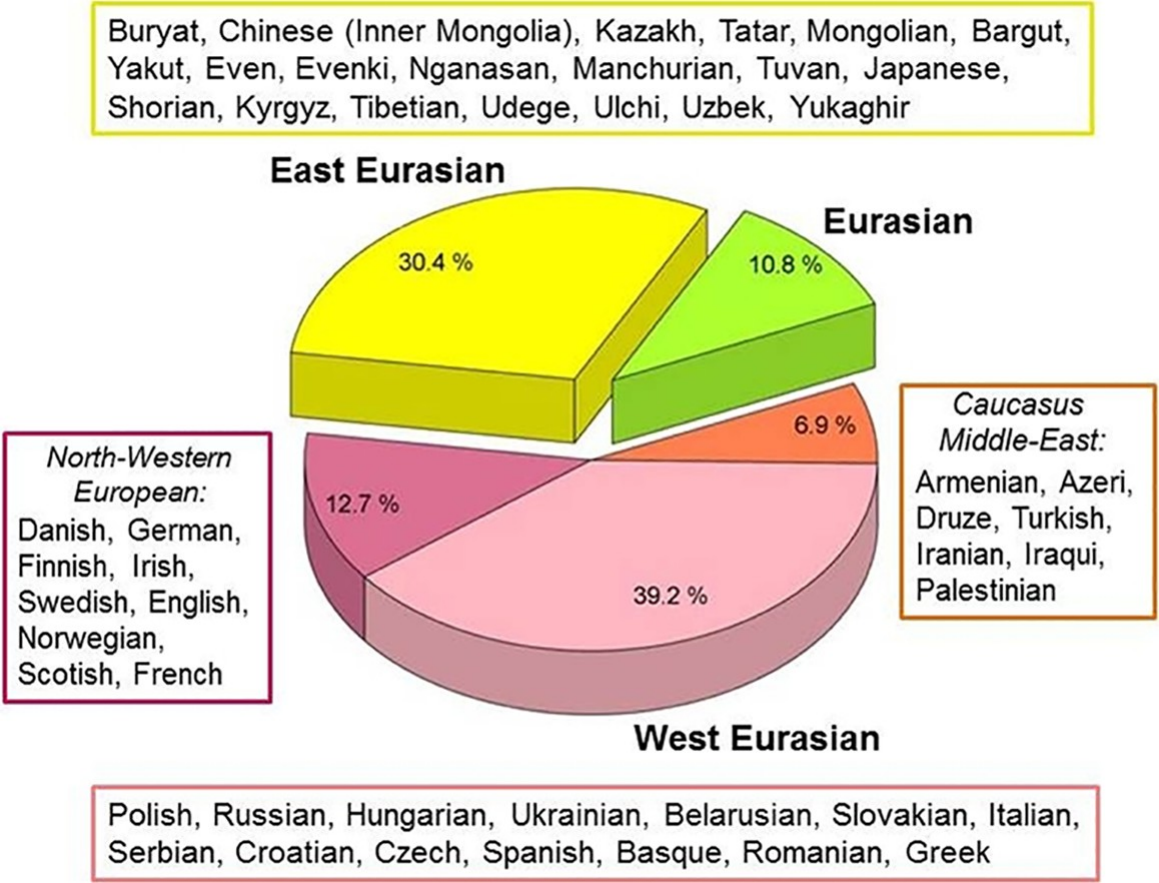
Skulls and sculpting craniofacial reconstructions of Hungarian Conqueror individuals. **A**: Karos2/52 mature aged leader with Europid anthropological features. **B**: Karos2/60 senile aged man with Europo-Mongoloid features. **C**: Karos2/47 adult woman with Europo-Mongoloid features.

The sculpting craniofacial reconstructions of three skulls from the Karos cemeteries (Fig 2) were carried out by Gyula Skultéty in cooperation with the Hungarian Natural History Museum, Department of Anthropology [39,40]. During the facial reconstruction, soft tissue layers were grafted back onto the plaster copy of the skulls carefully following the bone conformation to accurately recreate the facial features according to the published guidelines [41–43]. Facial reconstruction was performed by traditional sculpting anatomy, that is plasticine muscles were attached in their anatomically correct position [40,44]. The width of a muscle was determined by the ruggedness of the bone surface by means of a table compiled from measurements taken from 45 different points of the skull. These data have been collected by scientific methods [45].

## Results

### Phylogenetic study

**Fig 3:**
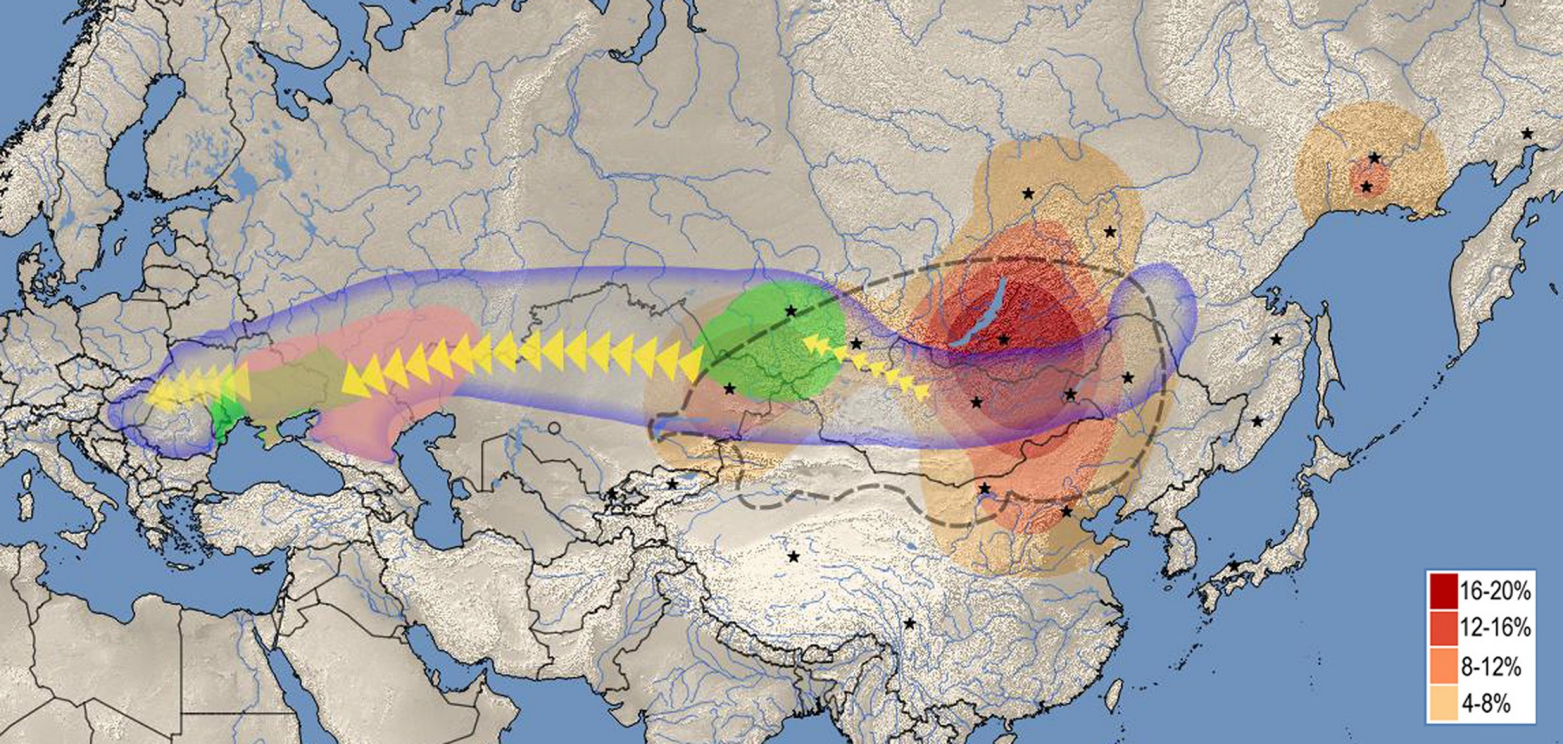
Phylogeographic origin of the 102 Conqueror maternal lineages. Data are summarized from S1 Fig. Origin of modern individuals with closest matches to Conqueror sequences are listed next to the indicated regions, ordered according to the frequency of appearances.

Using the NGS sequencing method combined with target enrichment, we could obtain 102 ancient mitogenome sequences, 78 of which are first reported in this paper, while 24 had been reported in [15]. The 102 sequences belong to 67 sub-Hg-s, and first we elucidated the phylogenetic relations of each Hg-s using M-J Networks as shown in S1 Fig. The closest sequence matches pointed at a well-defined geographical region in most cases, which is indicated next to the phylogenetic trees and is summarized on Fig 3.

Phylogenetic trees revealed that the Conqueror maternal lineages originated from two distant geographical regions; 31 were unequivocally derived from East Eurasia, while 60 from West Eurasia. The remaining 11 Conqueror Hg-s are ubiquitous in Eurasia. Out of the 60 west Eurasian lineages 13 are characteristic for modern Northwestern Europeans, while 7 have primarily Caucasus-Middle-East distribution.

As high similarity of mitogenomes infer recent common maternal ancestor, sequence correlation levels provide important phylogeographic information. Origin of modern individuals with closest matches to Conqueror sequences are listed on Fig 3. We detected a very prominent frequency of Hg *N1a1a1a1a*, represented by 7 Conqueror samples, while two more samples belonged to the progenitor *N1a1a1a1* lineage (S1 Fig; Network 36). *N1a1a1a1a* has Central Asian origin, as its current distribution is restricted to Kazakhstan, Altai, Buryat Republic and Russia, attesting that these areas were the center of expansion [46]. This Hg was detected in a Bronze Age Sintashta sample from Kazakhstan [47], an Iron Age Pazyryk Scythian [48] and an early Sarmatian sample [49], while its progenitor Hg *N1a1a1a1* has a wide Eurasian distribution [46]. Our phylogeographic data imply a probable expansion of *N1a1a1a1* from the European Pontic Steppe to Central Asia around the Bronze Age and its sub-clade *N1a1a1a1a* from Central Asia both to Inner Asia and back to Europe from the Iron Age. Besides many of the sequences showed close matches with samples from ancient cultures (S1 Fig), the most recurrent being the Bronze Age Srubnaya (Timber-grave) culture with 6 closely related Conqueror sequences. More than one related sequences were found to samples from Neolith-Bronze Age Hungary, Yamnaya-Eneolith Samara, Armenian Neolith-Bronze Age.

**Fig 4:**
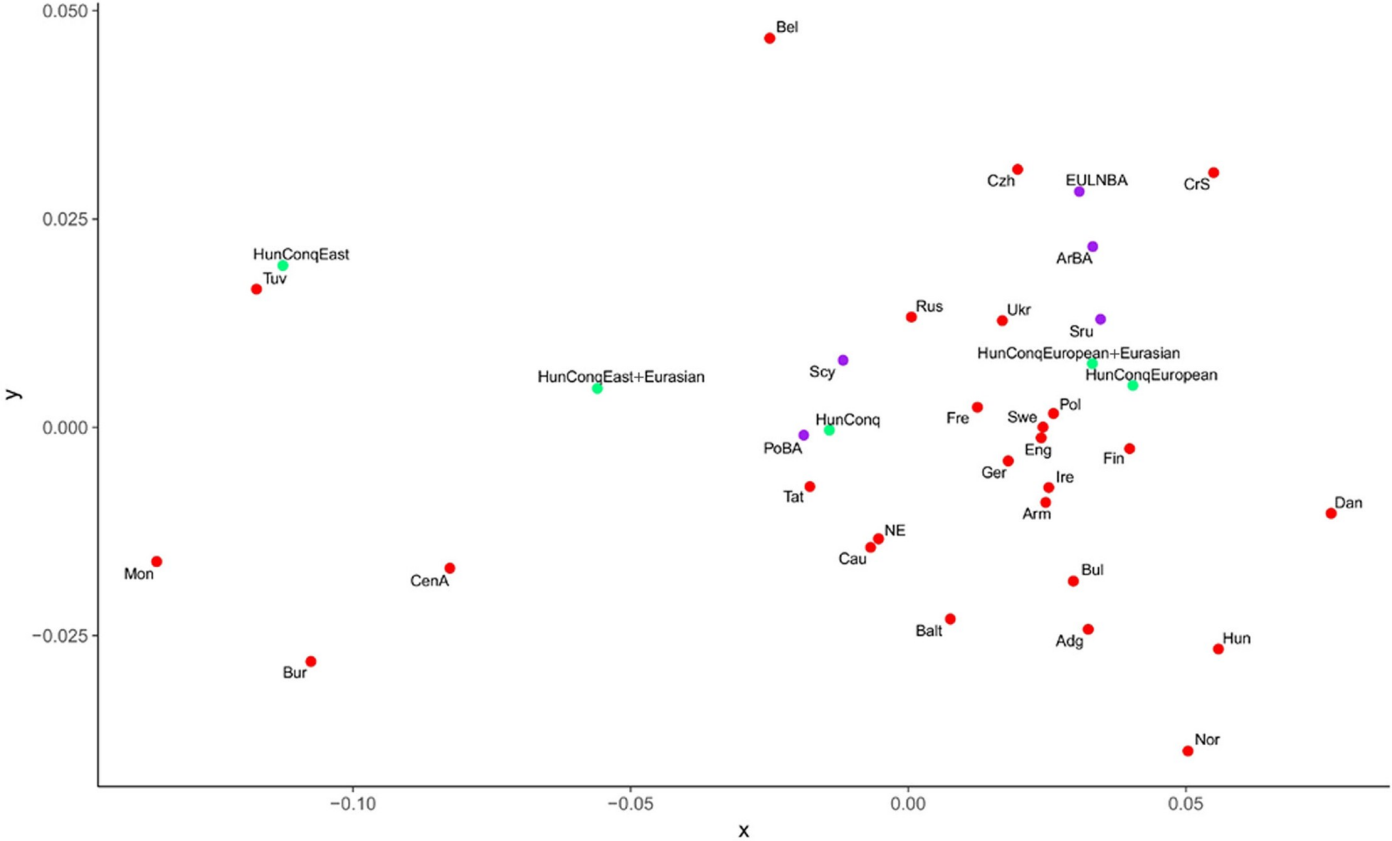
The most feasible origin and migration route of different components of the Hungarian Conquerors based on this study. Red heat map displays the geographic distribution of closest East Eurasian sequence matches to individual Conqueror samples. Stars denote geolocations of East Eurasian ethnic groups listed on S1 Fig (summarized on S1A Table), map was drawn from their frequency of occurence. Heat map designate the area from which the East Eurasian lineages most likely originated, well corresponding to the range of the ancient Xiongnu Empire outlined by dashed line. Areas where Asian and European Scythian remains were found are labeled green. Asian Scythians around Tuva correspond to the most probable sources of Eurasian lineages. Pink label shows the presumptive range of the Srubnaya culture, from where European lineages were most likely derived. Bluish line frames the Eurasian steppe zone, within which all presumptive ancestors of the Conquerors were found. The map was created using QGIS 2.18.4[51].

Distribution of the closest east Eurasian sequence matches outlines a well-defined geographic region (Fig 4, red heat map) centering around modern Buryatia-Northern Mongolia, with some extension through Tuva into Central Asia, an area well corresponding to the center and range of the ancient Asian Hun (Xiongnu) Empire especially considering that Yakuts, Evenks and Evens lived more south in the past [50].

Even though phylogenetic analysis may indicate the ultimate source region of individual maternal lineages, but these together do not necessarily correspond to actual populations, which has to be studied by population genetic methods.

### Population genetic study

**Fig 5:**
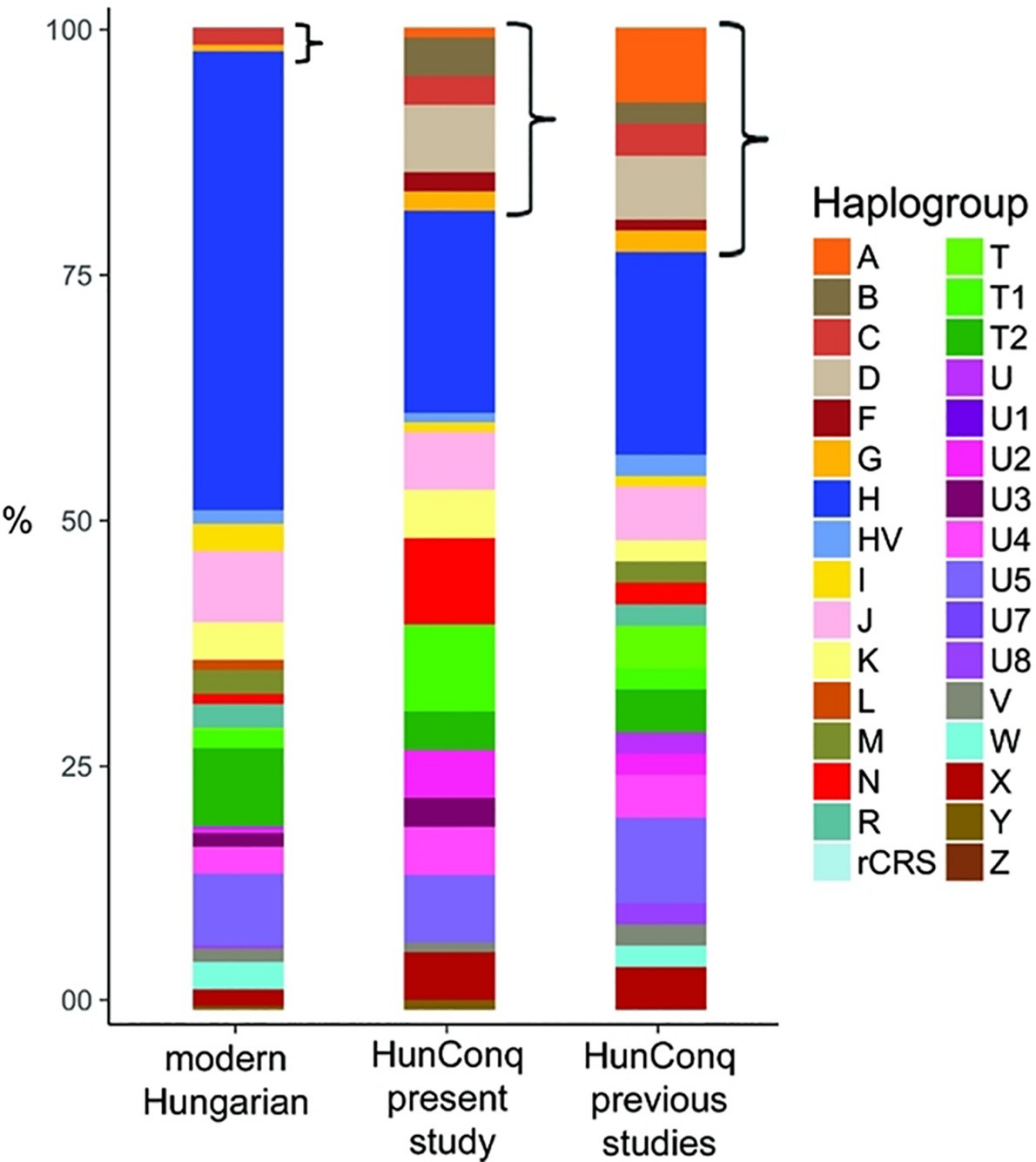
MDS plot from linearized Slatkin Fst values of S4A Table. Only populations from Table 1 were depicted, which showed close Fst and SHD distance values to the Conquerors. Abbreviations of population names are given in S3B Table.

As the studied samples apparently represent real Conqueror populations we measured their genetic distances from all recent and ancient populations. For increasing the resolution of the method we compared mitogenomes of populations, albeit this inherently reduces sample representativeness. Besides the traditional Fst distance calculations we used a novel approach [29], which calculates so called Shared Haplogroup Distances (SHD). The simple logic behind SHD is that sub-Hg-s originated from a single most recent common ancestor, thus presence of identical subgroups links population histories in an extent of sharing, which is proportional to the SHD value. Both pair-wise distance matrices are shown in S4A and S4B Table. The Fst and SHD methods gave comparable results (S4C Table), thus close distance values measured with both methods can be considered very plausible relationships. Latter populations are summarized on Table 1 and the MDS plot from linearized Slatkin Fst values of this subset is displayed on Fig 5.

**Table 1:** Fst and SHD distances of modern (rec) and ancient (arch) populations measured from different Conqueror subpopulations.

**A**										
**Conqueror subpopulation**	**all 102 samples**	**all 102 samples**	**60 European**	**60 European**	**60 European + 11 Eurasian**	**60 European** **+ 11 Eurasian**	**31** **East Eurasian**	**31 East Eurasian**	**31 East Eurasian + 11 Eurasian**	**31 East Eurasian + 11 Eurasian**
**pop. distance**	**Fst**	**SHD**	**Fst**	**SHD**	**Fst**	**SHD**	**Fst**	**SHD**	**Fst**	**SHD**
**Adg_rec**	0.03069	0.941297	0.01847	0.916744	0.01936	0.929424	0.13176	1.000000	0.08642	0.998740
**ArBA_arch**	0.03063	0.929942	0.01694	0.915418	0.01812	0.919066	0.11985	1.000000	0.08027	0.998740
**Arm_rec**	0.02265	0.938107	0.01425	0.938045	0.01308	0.935801	0.12225	1.000000	0.07625	0.992298
**BalBA_arch**	no data	0.920117	no data	0.882045	no data	0.890278	no data	1.000000	no data	0.975465
**Balt_rec**	0.02087	0.935454	0.02184	0.930559	0.02062	0.921932	0.10376	1.000000	0.06336	0.980575
**Bel_rec**	0.03520	0.918819	0.05475	0.910862	0.05163	0.927491	0.08100	0.945993	0.05312	0.953125
**Bul_rec**	0.02543	0.935656	0.01647	0.918425	0.01470	0.920755	0.13112	1.000000	0.08118	0.995443
**Bur_rec**	0.07933	0.924067	0.13283	0.976039	0.12876	0.959720	0.04334	0.947674	0.04783	0.929555
**Cau_rec**	0.01963	0.919181	0.02536	0.931991	0.02370	0.934971	0.08777	0.991891	0.05296	0.990639
**CenA_rec**	0.05540	0.912816	0.10903	0.980569	0.10397	0.983404	0.03136	0.895111	0.02984	0.905585
**CrS_rec**	0.04000	0.881627	0.03109	0.878611	0.02665	0.870518	0.15432	1.000000	0.09882	0.979546
**Czh_rec**	0.03158	0.891439	0.03319	0.903625	0.02864	0.871589	0.12000	0.990123	0.07398	0.957738
**Dan_rec**	0.03603	0.878530	0.01890	0.867675	0.01712	0.870875	0.16505	0.999586	0.10783	0.990991
**Eng_rec**	0.02249	0.899587	0.01663	0.884781	0.01561	0.884072	0.11607	0.998612	0.07224	0.990175
**EULNBA_arch**	0.03244	0.938095	0.02289	0.957061	0.02199	0.926604	0.11739	1.000000	0.07719	0.956861
**Fin_rec**	0.03113	0.901420	0.02458	0.895796	0.02262	0.895406	0.13771	0.999028	0.08810	0.989871
**Fre_rec**	0.01474	0.942980	0.00950	0.943249	0.00842	0.939393	0.10498	1.000000	0.06241	0.991027
**Ger_rec**	0.01871	0.934574	0.01890	0.925990	0.01661	0.932258	0.11106	1.000000	0.06581	0.998740
**Hun_rec**	0.03190	0.890426	0.02364	0.885860	0.02125	0.890290	0.15281	0.984949	0.09680	0.975225
**Ire_rec**	0.02310	0.919608	0.02109	0.888993	0.01971	0.898154	0.11360	0.999485	0.07024	0.997708
**Mon_rec**	0.09321	0.933012	0.14892	0.992259	0.14527	0.988984	0.04207	0.924401	0.05331	0.926916
**NE_rec**	0.01698	0.944518	0.01908	0.939517	0.01813	0.933443	0.08938	0.994413	0.05383	0.983392
**Nor_rec**	0.03419	0.933588	0.02570	0.909962	0.02511	0.918304	0.14542	1.000000	0.09406	0.996202
**PoBA_arch**	-0.02559	0.940929	-0.00459	0.907988	-0.00849	0.922299	0.03135	1.000000	-0.00159	1.000000
**Pol_rec**	0.03021	0.890441	0.02545	0.889243	0.02223	0.877829	0.13205	0.997879	0.08273	0.979629
**Rus_rec**	0.02505	0.873977	0.02999	0.898988	0.02708	0.870649	0.09965	0.991407	0.06093	0.956590
**Scy_arch**	0.01516	0.961319	0.02678	0.933904	0.02411	0.943931	0.08116	1.000000	0.04594	0.998740
**Sru_arch**	0.00852	0.913433	-0.00190	0.896586	-0.00393	0.894591	0.11688	1.000000	0.06535	0.995443
**Swe_rec**	0.01790	0.895493	0.00936	0.889648	0.00798	0.887236	0.12056	1.000000	0.07260	0.991380
**Tat_rec**	0.00941	0.858003	0.02367	0.928852	0.01849	0.877419	0.07007	0.968067	0.03399	0.920860
**Tuv_rec**	0.07980	0.914069	0.14084	0.982747	0.13197	0.894807	0.04943	0.971963	0.04569	0.883068
**Ukr_rec**	0.02527	0.920220	0.02799	0.889057	0.02471	0.903087	0.10272	1.000000	0.06250	0.998740
**B**										
**Similarity levels in descending order**										
**Fst value**					0.0–0.01	0.01–0.02	0.02–0.03	0.03–0.05		
**Shared Hg. Frequency Distance (SHD) value**					0.86–0.88	0.88–0.90	0.90–0.92	0.92–0.94		

(A) Distance values. (B) Color code of distance values. Only populations which showed close distance values with both methods for any of the Conqueror subpopulations are displayed here from S4C Table. Details of subgrouping are shown in S1A and S1B Table.

From the Conqueror population Volga Tatars have the smallest overall distance with both methods (Table 1, all 102 samples), and accordingly they are positioned very close on the MDS plot implying closest genetic relation at the population level.

As for the further analysis it is important to point at important differences between the Fst and SHD methods, which is illuminated by their different patterns on Table 1 and S4C Table. Fst is best suited to measure genetic distance between isolated populations where the effect of evolutionary sequence divergence is determining [29] and in case of population admixture it shall identify populations with similar admixture patterns or at best the predominant source. In contrast SHD is capable of indicating admixing sources and its value is proportional to admixing ratios [29]. The presence of 30% east Eurasian and 60% west Eurasian Hg-s in the Conquerors is a clear indication of past admixtures whose sources should be identified. Besides Volga Tatars the Fst similarity list includes exclusively west Eurasian populations (Table 1) most likely related to the majority admixture components. In contrast SHD clearly identifies potential east Eurasian admixture sources; Buryats (Bur), Central Asians (CenA), Mongolians (Mon) and Tuvans (Tuv). The efficacy of the SHD method can be demonstrated by an artificial partition of the Conqueror population into east and west Eurasian components (S1B Table) and performing the same analysis with each component. Though this grouping correspond just to speculative populations not real ones, the results clearly confirm our above claims. The entire Conqueror population shows nearly identical Fst distance patterns to that of its dominant European subset (Table 1), irrespective of the presence of the 11 Eurasian lineages and accordingly the European and European+Eurasian Conqueror subpopulations map very close on the MDS plot (Fig 5). On the other hand now both methods identify the same east Eurasian populations close to the Conqueror east Eurasian subsets which had been identified within the entire population just by SHD, moreover nearly the same SHD distance values are measured in the subsets as in the total Conqueror population (Table 1). Thus SHD is much more sensitive in correctly identifying genetic similarity levels to multiple potential source populations, however it does not necessarily inform about real admixing sources, as admixtures could have happened multiple times.

Our another novel algorithm MITOMIX [29], can reveal more details of admixture histories as it performs a hypothesis independent search to find the best admixture combinations from available populations giving the smallest SHD value from a test population. MITOMIX indicates that if all modern and ancient populations are considered as potential source, the Conquerors are best admixed from 26–38% modern Belarusians, 19–34% Tuvans, 18% ancient Baltic Late Bronze Age and 13% Srubnaya populations (S5A Table). Other possible admix components may include 9–26% Volga Tatars, Poltavka-Potapovka, Sintastha and Combed Ware populations. Thus MITOMIX principally derives East Eurasian Conqueror lineages from Tuvans, Belarusians and Volga Tatars, though latter two are located now in Europe. Belarusians comprise 22% Lipka Tatars in our dataset [52], who arrived to Europe after the Conquerors’ era, but seemingly with similar Hg-s. Belarusians are best admixed from Russians, Romanians and Central Asians (S5B Table), while Tuvans are best admixed from Central Asians and Mongolians with some Yakut and European elements (S5C Table). Main admixture components of Volga Tatars are 24–46% Conquerors, 20–50% Russians, 16–30% Mansis+Khantys (Yug) and 9–18% Norvegians (S5F Table).

Though these results should be interpreted with caution due to imperfect population data, they sketch the most feasible population processes; ancestors of modern Tuvans could be the nearest sources of east Eurasian Conqueror lineages and accordingly they map very close to the eastern Conqueror subset (Fig 5). In turn Tuvans originated mainly from Inner and Central Asian ancestors, and all these are detected in the entire Conqueror population by SHD (Table 1). Belarusians come to play just because of their similar European and Lipka Tatar components, while Volga Tatars still harbor a salient Conqueror like subpopulation consisting of both eastern and western lineages (S5F Table, Table 1). Thus MITOMIX confirms the direct genetic relation to Volga Tatars, detected by both Fst and SHD, and this relation must be closest in time.

MITOMIX derives west Eurasian Conqueror lineages by augmenting the European components of above populations, with admixtures from Baltic Bronze Age (BalBA), Srubnaya (Sru) and Poltavka-Potapovka (PoBA) populations (S5A Table). This is again in line with Fst data, as Srubnaya maps closest to the west Eurasian Conqueror subset while PoBA maps very close to the entire Conqueror group (Fig 5). Although we have shown that limited sample size may give meaningful results [29], these inferences vindicate caution as we have just 8 mitogenomes from PoBA, 14 from Srubnaya and no sequences from BalBA.

When only ancient populations are considered as a source, the best admix includes 36–44% Poltavka-Potapovka, 18–20% Baltic Bronze Age, 11–29% Combed Ware, 14–18% Sintashta and 14% Srubnaya components (S5A Table), all of which are comprised of solely west Eurasian Hg-s. However ancient MITOMIX gives significantly higher SHD distances signifying that our ancient database lacks important east Eurasian components.

## Discussion

The most plausible interpretation of the phylogenetic and population genetic results is that the majority of eastern lineages were ultimately derived from Inner Asia which then migrated to Central Asia where they admixed with Eurasian lineages before moving to Europe, where they in turn incorporated west Eurasian elements. As the Conquer population was apparently assembled from multiple sources this raises the questions as to when did the admixtures happen, which ancient populations could have been the source and how can our results be reconciled with historical, archaeological, anthropological and other genetic data.

### Relation to Volga Tatars

Our data testify closest genetic relation to this modern population. Volga Tatars incorporate three main ethnic components [53]; the Volga Bulgars, which arrived in the 8^th^ century, and intermingled with local Scythian and Finno-Ugric populations, then in the 13^th^ century Kipchak Tatars of the Golden Horde brought a final Central-Inner Asian genetic layer and their language to the region. MITOMIX seems to identify these historical components, as Finno-Ugric Mansis and Khantys (Yug) comprise a major component of Volga Tatars besides Russians, while Scythians also appear among their potential sources (S5F Table). Our remarkable result is that the Conquerors seem to provide a predominant (26–41%) component of Volga Tatars (S5F Table), while the opposite value is significantly lower (9–26%; S5A Table). This asymmetry is due to the absence of some Tatar components, like Finno-Ugric ones, from the Conquerors. Thus our data indicate that rather Volga Tatars harbor a “Conqueror like” genetic component than the opposite, which may be linked historically to the Volga Bulgars.

This assumption is well supported by archaeological, anthropological and historical sources; Volga Bulgars were one of the few groups which had the same partial horse burial customs [54] and similar grave goods as the Conquerors. Both groups are characterized by similar anthropological types [55], and practiced identical symbolic trepanation customs [56] which is documented with such a high frequency just among the related Danube Bulgars [57]. Historical data link both groups to the Onogurs [58], the Conquerors must have belonged to the Onogur tribal union, as the name “Hungarian” is derived from “Onogur” [5,59]. Historical sources imply that ruling dynasties of both groups might be traced back to the Hun ruling dynasty [60]. Taken together the direct genetic relation of the Conquerors to Onogur-Bulgar ancestors of Volga Tatars is very feasible.

### East Eurasian relations

Identifying admixture sources further back in time is more precarious, but ancient DNA and historical data allow drawing some inferences. We may rely on the better grounded Bulgar prehistory and the Tuvan genetic affinity of the Conquerors, which define a time window, a geographic region and a migration route through Central Asia to the Pontic steppes.

Both anthropological [61] and genetic data [47,62] indicate that until the Bronze Age Asia was populated mainly by Europid Sintashta-Andronovo people west of the Altai, while populations with Mongoloid traits and genes were confined east of the Altai. The first eastern Hg lineages appeared in West Siberia at the beginning of Bronze Age [63], in the Altai at the Middle Bronze Age [64], while in Central Asia just around the 6^th^ century BC corresponding to the Xiongnu invasions [65].

In the Iron Age the Tuva region was inhabited by Scytho-Siberians, which were already an admixed population of east and west Eurasians [49]. During the Iron Age Scytho-Siberians further admixed with European Scythians in both directions, giving rise to 18–26% eastern lineages in European Scythians by the 2^nd^ century BC [49,66]. Before 200 AD Tuva became part of the Asian Hun (Xiongnu) empire and Hun migration from Mongolia to west through Altai and Tuva lead to a significant increase of Mongoloid anthropological components in Central Asia between the 3^rd^ century BC and 2^nd^ century AD [61,67]. Thus western (Eurasian) lineages in the Tuva region can be attributed to Andronovo and Scythian periods, while appearance of east Eurasian lineages to Asian Scythian and Xiongnu periods. Genetic similarity between Xiongnu and modern Turcic and Mongolian speaking groups indicate that the Xiongnu period played a determining role in shaping the genetic profile of Eastern and Central Asia [68], supporting our phylogeographic implications (Fig 4) that Xiongnus could be among the ancestors of the Conquerors. A HVR based population genetic study [69] has indeed shown similarity between Xiongnus and among others Conquerors, as well as Volga Tatars. At any case, the eastern Hg lineages must have been brought to Europe by nomadic groups originating from this region.

During the first centuries AD Northern Xiongnus were expelled from Inner Asia and escaped westward [70], leading to another major wave of east Eurasian gene flow into Central Asia, then further to the Pontic steppes. According to some archaeologists traces of European Huns can be detected on the Pontic steppe already in the 2^nd^ century AD [71], but European Huns entered history just from the middle of the 4^th^ century as an empire. The Xiongnu origin of European Huns has been accepted by most historians [72–74], but evidences are scarce.

A decade after the fall of the European Hun empire (472 AD) another grouping of Turkic tribes, the Ogurs appeared on the Pontic steppe from Central Asia. The Onogurs are the first nomadic groups from the east, which are reliably connected by historical sources to the later appearing Bulgars, and less reliably to the Conquerors [58]. Onogurs had been part of the Hunnic people, and after the death of Attila’s son Irnik, European Hun remains fused with the Onοgurs [58]. The ensuing Avar invasion brought Onogur groups to the Carpathian Basin, others became part of the later Danube Bulgar and Volga Bulgar states.

The succeeding group arriving from East Eurasia to the Pontic steppes in the middle of 6th century were the Avars, who established an empire in the Carpathian Basin lasting for three centuries [75]. It is relevant to note that none of the Hungarian medieval sources know about Avars, presumably because they were not distinguished from the Huns [2], as many foreign medieval sources also identified Avars with the Huns [3].

Subsequent east-west migrations are connected to Göktürk, Kipchak and Mongolian groups, but these could have minor effect on the Conquerors as mostly arrived after the 10^th^ century, moreover most Turkic loanwords in Hungarian originate from West Old Turkic [76], the Oghur Turkic branch associated with previous Turkic speaking groups as Onogurs, Bulgars, Khazars and maybe the Avars.

Taken together genetic and historical data refer to four major groups delivering significant east Eurasian lineages to Europe which could be connected to the Conquerors; Asian Scythians, Huns, Onogurs and Avars. Of these groups we have mitogenome sequences just from European Scythians [66]. Despite the presence of eastern lineages in European Scythians, they rather resemble to the European component of the Conquerors (Table 1) suggesting that eastern Conqueror lineages arrived with later invasions. Thus our genetic data are in line with historical sources which indicate that Onogurs could have been a major source of the Conqueror population, nevertheless it is obvious that Hun, Avar and Onogur waves intermingled with each other and local populations.

### West Eurasian relations

According to our data the best fitting sources of the west Eurasian lineages are the Late Bronze Age Srubnaya (Timber-grave) culture (~1,850–1,200 BC) and its ancestors the Potapovka (~2,500–1,900 BC) and Poltavka (~2,900–2,200 BC) cultures (Table 1). The Srubnaya was a nomadic culture on the Pontic-Caspian steppe, both their genetic composition and life style being closely related to the partly contemporary eastern Andronovo and Sintashta cultures, together constituting the steppe Middle-Late Bronze Age (MLBA) population. Latter was descended from the genetically tightly clustering steppe Early-Middle Bronze Age (EMBA) Yamnaya-Afanasievo-Poltavka cultures with the addition of an European Neolithic farmer genetic layer [77,78]. As a result, the steppe MLBA population very much resembled genetically to the European Late Neolithic/Bronze Age (EULNBA) populations [77], providing an explanation for the similarity of the Conquerors to EULNBA populations (Table 1, Fig 5), the appearance of a considerable number of modern European and Northwestern European maternal lineages close to the Conquerors (S1 Fig) and the presence of European Y-chromosomal Hg-s *R1b-M269* and *I2a* in the Conquerors, reported in our previous study [13].

The Armenian Bronze Age (ArmBA) population also appears very close to the Conquerors (Table 1, Fig 5), that may be explained by the 48–58% Armenian-like Near East ancestry of the steppe EMBA populations [77], which was ultimately derived from early Iranian farmers [78]. This genetic layer may also explain the appearance of modern populations from the Caucasus region (Cau, Adg, Arm) close to the Conquerors both in population genetic (Table 1) and phylogenetic analysis, (S1 Fig). Nevertheless a more recent admixture from this region is also plausible, as all presumptive carriers of the east Eurasian lineages contacted the Caucasus region during their westward migrations.

### Finno-Ugric relations

Surprisingly we did not find significant genetic relations to Finno-Ugric groups. Though population genetic analysis indicates some connection of the European Conqueror component to modern Finnish (Fin) and Baltic (Balt) people, but no relation to Saamis (Sam), Mansis and Kanthys (Yug) (S4C and S5A Tables). The Baltic relation of the European component seems to appear already in the Baltic Late Bronze Age (BalBA, 1000–230 BC), [79] measured with the SHD method (Table 1). BalBA genomes cluster with modern Lithuanians and Estonians, and lack eastern mtDNA Hg-s and Y-chromosomal haplogroup *N-tat*, (new name N1a1) which is typical for Uralic speaking groups, thus Estonians must have received their east Asian-Siberian components after the BalBA period, from a different source [79]. According to our data BalBA is best admixed from the closely related Scandinavian Neolith-Bronze Age (NNBA), Afanasevo and European Neolithic populations (S5D Table), so it is unlikely connected to Finno-Ugric groups. As only 7 Estonian mitogenomes are available, they were grouped with other modern Baltic populations (Balt; S3B Table), so the similarity of these to the Conquerors probably derives from BalBA heritage. The connection to modern Finnish population can also be explained from BalBA and steppe MLBA components which are present in modern Scandinavians, as Finnish sequence matches regularly appear together with Danish ones on our phylogenetic trees (S1 Fig, Networks; 14, 15, 19, 25, 27, 30, 35, 40, 42, 43, 49, 52, 56).

Moreover, *Y*, *B* and *N1a1a1a1a* Hg-s have not been detected in Finno-Ugric populations [80–84], implying that the east Eurasian component of the Conquerors and Finno-Ugric people are probably not directly related. The same inference can be drawn from phylogenetic data, as only two Mansi samples appeared in our phylogenetic trees on the side branches (S1 Fig, Networks; 1, 4) suggesting that ancestors of the Mansis separated from Asian ancestors of the Conquerors a long time ago. This inference is also supported by genomic Admixture analysis of Siberian and Northeastern European populations [85], which revealed that Mansis received their eastern Siberian genetic component approximately 5–7 thousand years ago from ancestors of modern Even and Evenki people. Most likely the same explanation applies to the Y-chromosome N-Tat marker which originated from China [86,87] and its subclades are now widespread between various language groups of North Asia and Eastern Europe [88].

It must be emphasized that Finno-Ugric groups are underrepresented in our population database, as we have no mitogenomic data from Komis, Maris, Mordvins and Udmurts and only limited samples from Mansis, Kanthys, Saamis and Estonians. Therefore appearance of Finno-Ugric matches from a more representative dataset cannot be excluded, but our data imply that incidental Finno-Ugric link is rather expected in the European component if any.

### Genetic relation of different Conqueror cemeteries

**Fig 6:**
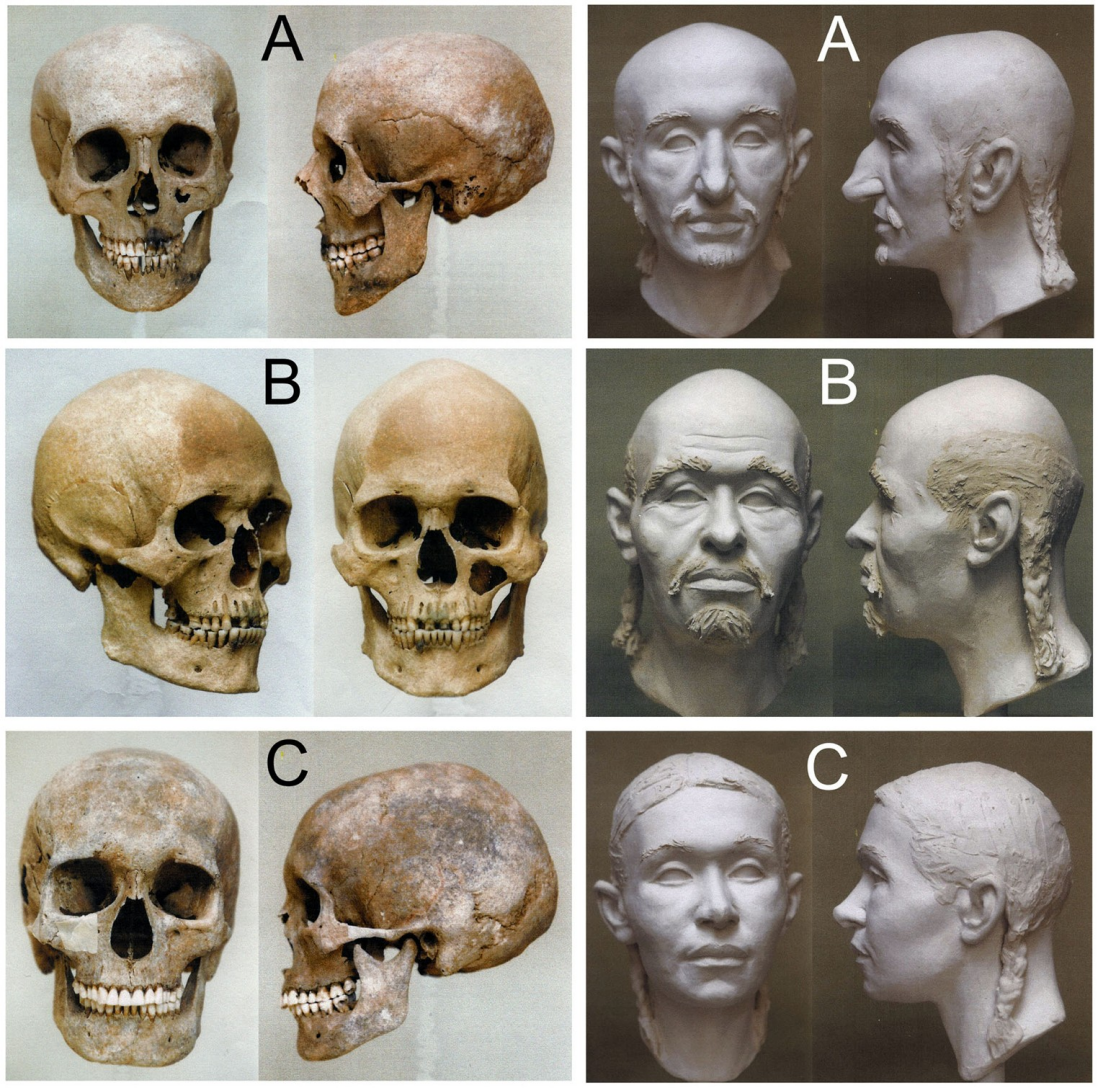
Comparison of major Hg distributions from modern and ancient Hungarian populations. Asian main Hg-s are designated with brackets. Major Hg distribution of Conqueror samples from this study are very similar to that of other 91 Conquerors taken from previous studies [11,12]. Modern Hungarians have very small Asian components pointing at small contribution from the Conquerors. Of the 289 modern Hungarian mitogenomes 272 are newly deposited [29].

Archaeologist presume that the rich 10th century cemeteries of Karos and Kenézlő comprise the Conqueror military elite, raising the question as to what extent can our findings be generalized to the entire Conqueror population. Our fragmentary data from other cemeteries indicate the presence of the same eastern and western genetic components (S1 Fig, Networks; 3, 4, 12, 36), moreover [12] and [11] reported 91 other Conqueror HVR haplotypes from 24 cemeteries (Fig 1), which show very similar major Hg distribution to our samples, with even larger proportion of Asian major Hg components (Fig 6).

Thus our conclusions probably apply to the entire Conqueror population, but definitely to the 10^th^ century immigrant military elite characterized with partial horse burials, though further mitogenomic and genomic data are required for the accurate answer.

We have determined the maternal lineage of the majority of samples from the three neighboring Karos cemeteries, and found likely maternal relatives with identical mtDNA genomes within cemeteries allocated into the same circles on the phylogenetic trees in S1 Fig (summarized in S1C Table), but surprisingly no identical haplotype was found between the three Karos cemeteries. The only exceptions are the two chiefs; Karos2/52 and Karos3/11, who had identical *X2f* maternal haplotypes and *I2a1* Y chromosomal haplotypes (data not shown), so were probably brothers. This indicates that these neighboring communities did not intermarry perhaps because of different group-identity. Furthermore the east Eurasian haplogroup lineages from the three Karos cemeteries indicate a discernible structuring (S1D Table); the Karos3 cemetery has a definite south-east Chinese affinity, the Karos1 a North-East Siberian affinity, while the Karos2 lineages are widely distributed from East to Central Asia. In contrast, despite the low number of samples analyzed from other Conqueror cemeteries we detected potential relatives with identical mtDNA genomes between distant cemeteries (S1C Table). This suggests that individual tribes might have been split and fragments of different tribes settled together upon the conquest.

### Relation of the Conquerors to modern Hungarians

Modern Hungarians are genetically very similar to their European neighbors [89] nevertheless they contain some 3–5% east Eurasian components traceable with uniparental markers [29,90,91], (Fig 6). Genome wide SNP data also detected the presence of 4% east Asian component in modern Hungarians [92] with an approximate time of admixture dated to the first millennium AD, corresponding to the invasions of Huns, Onogur-Bulgars, Avars and Hungarian Conquerors from the Asian steppes, which are completely in line with our results.

Thus genetic heritage of the Conquerors definitely persists in modern Hungarians, but they contributed to less than 10% of the recent Hungarian gene pool, as they were not alone to bring in east Eurasian lineages. This dilution could have started with the conquer, as contemporary local population size in the Carpathian Basin was estimated larger than that of the Conquerors [93,94]. Anthropological data also have the same implication, as the Conquerors differed from the subsequent Árpádian Age population, which was more similar to preconquest Avar Age populations [95,96]. According to early anthropological studies people of the Avar and Conquest age Carpathian Basin were very heterogeneous and immigrants arrived in several phases between the 5^th^ and 9^th^ centuries [97], which in our view admixed with the autochthonous population, of which genetic data are still barely available between the Bronze Age and Conquest period.

The large genetic diversity of the Conquerors which seemingly assembled from multiple ethnic sources and their relative low proportion, having no lasting effect on Hungarian ethnogenesis, raises doubts about the Conqueror origin of the Hungarian language. Even if our samples represent mainly the Conqueror elite, the “elite dominance” linguistic hypothesis seems inconsistent when it presumes that the same Turkic elite was first readily assimilated linguistically by Finno-Ugric groups, and then it assimilated locals of the Carpathian Basin. Turkic character of the Conquerors is indicated by their “Turk” denomination in contemporary sources as well as Turkic tribal names and person names of tribe leaders of the conquest-period [98]. Above data infer that preconquest presence of the language in the Carpathian Basin, is an equally grounded hypothesis, as had been proposed by several scientists (a summary in English is given in [99]).

## Conclusions

The large diversity of Hg-s detected in the Conquerors reflects a quite complex genetic history, which was summarized from our data on Fig 4. Their uniform archaeological findings and predominantly Europid anthropological features (Fig 2, S1A Table) indicate a long lasting admixture on the Pontic steppe, thus their final composition was likely formed there during the last centuries prior to the conquest.

A significant fraction of their ancestors undoubtedly arrived from Asia, which probably originated from Asian Scythians and Xiongnus. On the Pontic steppes Asian nomads assimilated with descendants of the Srubnayas and this mixed population could have been the basis of many medieval Pontic nomadic groups, including Conquerors. Their ancestors were certainly part of the European Hun Empire, the succeeding Avar and Bulgar empires, and when they came into power they very probably incorporated European Hun remains, as recognized previously [100]. Our genetic data seem to support the Hun-Conqueror connection which could have been the basis of the historical-cultural Hungarian Hun tradition [3]. Direct genetic relation of the Conquerors to medieval Onogur-Bulgars warrants further studies, as they are linked by archaeological, anthropological and historical data as well as our population genetic indications.

Our conclusions are well supported by anthropological studies, which found analogies of the lower class Conqueror individuals on the eastern European steppes, but parallels of the upper warrior class were mainly found at the fringes of the Xiongnu empire, in South Siberia and South-Central Asia [101]. Finally our data indicate that all potential ancestors of the Conquerors were steppe nomadic people, which is in full agreement with their archaeological legacy.

## Supporting information

S1 Appendix Basic description of the studied cemeteries. (DOCX)

S1 Fig Phylogenetic trees (1–58), made with Median-Joining Network, from mtDNA sequences of the 102 Hungarian Conquerors. Phylogenetic trees are arranged in alphabetic order according to haplogroups. The 67 sub-haplogroups are depicted on 58 Networks. Samples falling into the same sub-haplogroup with the studied sample are encircled. The smallest colored circles represent one individual; circle sizes are proportional to the number of individuals with identical sequences. (When large number of sequences with few phylogenetically informative SNP-s are aligned, the algorithm may force the most similar but not identical sequences into the same large circle.) Green circles identify Hungarian conqueror samples, red circles represent modern samples, and violet circles correspond to ancient samples. A few ancient samples belonging to the shown haplogroup could not be properly aligned due to incomplete sequences, and these were connected with dashed line to the tree. Number of crosslines between neighboring circles denotes mutation distances. Length of connecting lines is irrelevant, as they were modified in order to fit page. Genebank accesion number and origin of samples closest to the studied conquerors are listed next to the circles. Known Conqueror Y-chromosome haplogroups were added in blue color. We summarized the probable origin of the samples’ Hg lineage in colored framed text. In some cases comments are given next to the trees.
(PDF)

S1 Table Details of Hungarian Conqueror samples. Table a. Description of samples including anthropological and archaeological details. Haplogroups and closest matching sequences are also summarized from S1 Fig. Probable origins of Hg lineages are color coded, codes and details of anthropological ages are given to the right. Table b. Conqueror subpopulations considered in population genetic analysis. Sample groupings are based on S1a Table. Table c. List of samples with Identical mtDNA sequences indicating potential direct maternal relations. Table d. Distribution of the East Eurasian Hg-s in the three Karos cemeteries.
(XLSX)

S2 Table Sequence data. Table a. Details of NGS data for each samples. Samples highlighted with blue were published in [15]. Lowest coverage sequences containing larger gaps were highlighted with pink. Contamination was estimated with two methods; a) using the Schmutzi algorithm and b) calculating the proportion of reads which did not correspond to the consensus sequence in diagnostic positions as in [15]. All DNA extracts were partial UDG treated, except Karos2/52 for which UDG treated and non treated libraries were merged to increase coverage and misincorporation values of both libraries are provided (values of non treated labelled with *). Table b. List of SNP-s provided against rCRS. Following the recommendations in [102], we excluded common indels (hotspots) at nucleotide positions: 309.1C(C), 315.1C, 523-524del (or 522-523del), 3106del, 16182C, 16183C, 16193.1C(C), 16519C. Red numbers indicate SNP-s missing due to lack of coverage. Haplogroup was determined both with HaploGrep (based on SNP list) and HaploFind (based on Fasta sequences), haplogroups defined differently are highlighted with yellow background. In the analysis we used consistently HapoFind defined Hg assignments.
(XLSX)

S3 Table Population database. Table a. Modern population database with NCBI GenBank accession numbers and haplogroups given next to population abbreviations (provided in S3B Table). 314 newly deposited mitogenomes from [29] including 272 Hungarian, 46 French, 18 Croatian, 12 Belgian, 11 German and 12 Romanian samples are provided at the end of the list. Table b. Summary of the modern (top) and ancient (below) population database with abbreviations used in this study. In case of low sample size related neighboring populations were merged whose list is provided next to their group name. Table c. Ancient mtDNA genome database. Pink background highligts samples, for which sequence was not available, so these were only included in SHD analysis. Yellow background highlights haplogroups, which were classified differently by Haplofind than published originally (Haplofind/original). Supplementary references are provided below the table. Table d. Ancient samples considered only in the phylogenetic analysis but not used in population genetic analysis. Supplementary references are provided below the table.
(XLSX)

S4 Table Population genetic data. Table a. Pairwise Fst (top) and linearized Slatkin Fst (below) matrix of population distances between all combinations of modern and ancient population. In the upper right part of the table probability values are depicted, + correspond to significant P values (<0.05), while—means not significant P values. Color code (provided below the table) highlights the best similarity levels. Abbreviations of population names are given in S3B Table. Table b. Pair-wise Shared Haplogroup Distance (SHD) values measured between all combinations of modern and ancient populations. Color code (provided below the table) highlights the best similarity levels. Abbreviations of population names are given in S3B Table. Table c. Comparison of population genetic distance values measured with two different methods (Fst and SHD) between Hungarian Conqueror subpopulations and all ancient (arch) and modern (rec) Eurasian populations. Color code (provided below the table) highlights the best similarity levels. Abbreviations of population names are given in S3B Table.
(XLSX)

S5 Table MITOMIX results. Table a. Best MITOMIX results for the entire Conqueror population from available population Hg frequency data (S3A and S3C Table). Data were computed from all possible proportions of all possible population combinations (top) or just from contemporary or older ancient populations (below). The best 64/20 combinations giving the smallest SHD distances to the Conquerors are listed. East Eurasian populations are highlighted with yellow, Volga Tatars are highlighted with green. Finno-Ugric groups (Yug) do not appear among the potential sources. Table b. Best MITOMIX results for modern Belarussians from available population Hg frequency data (S3A and S3C Table). Data were computed from all possible proportions of all possible population combinations. The best combinations giving the smallest SHD distances are listed. Table c. Best MITOMIX results for modern Tuvans from available population Hg frequency data (S3A and S3C Table). Data were computed from all possible proportions of all possible population combinations. The best combinations giving the smallest SHD distances are listed. Table d. Best MITOMIX results for the ancient Baltic Bronze Age population from available population Hg frequency data (S3A and S3C Table). Data were computed from all possible proportions of all possible population combinations (top) or just from contemporary or older ancient populations (below). The best combinations giving the smallest SHD distances are listed. Table e. Best MITOMIX results for the ancient Srubnaya population from available population Hg frequency data (S3A and S3C Table). Data were computed from all possible proportions of all possible population combinations (top) or just from contemporary or older ancient populations (below). The best combinations giving the smallest SHD distances are listed. Table f. Best MITOMIX results for Volga Tatars from available population Hg frequency data (S3A and S3C Table). Data were computed from all possible proportions of all possible population combinations. The best 106 combinations giving the smallest SHD distances to the Volga Tatars are listed. European Scythian (Scy) and Tuvan (Tuv) admixture sources are highlighted with yellow. Finno-Ugric groups (Yug) are among the major sources.
(XLSX)
