# Correction: Mitogenomic data indicate admixture components of Central-Inner Asian and Srubnaya origin in the conquering Hungarians

**DOI:** 10.1371/journal.pone.0208295

**Published:** 2018-11-29

**Authors:** Endre Neparáczki, Zoltán Maróti, Tibor Kalmár, Klaudia Kocsy, Kitti Maár, Péter Bihari, István Nagy, Erzsébet Fóthi, Ildikó Pap, Ágnes Kustár, György Pálfi, István Raskó, Albert Zink, Tibor Török

Multiple figure images are incorrectly switched. The image that appears as Fig 6 should be Fig 2, the image that appears as Fig 2 should be Fig 3, the image that appears as Fig 3 should be Fig 4, the image that appears as Fig 4 should be Fig 5, and the image that appears as Fig 5 should be Fig 6. The figure captions appear in the correct order. Please see the correct Figs 2–6 below.

**Fig 2:**
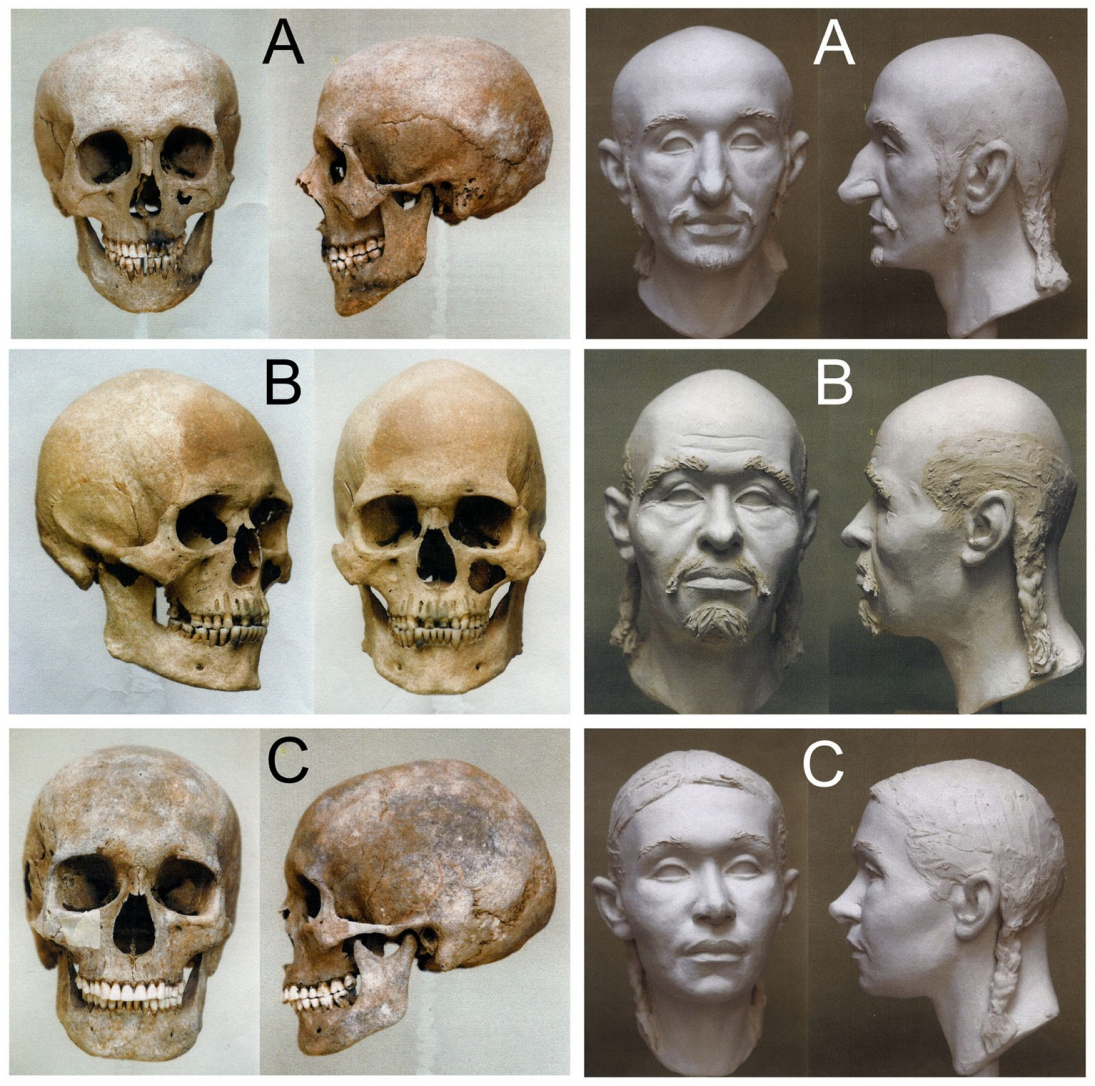
Skulls and sculpting craniofacial reconstructions of Hungarian Conqueror individuals. **A**: Karos2/52 mature aged leader with Europid anthropological features. **B**: Karos2/60 senile aged man with Europo-Mongoloid features. **C**: Karos2/47 adult woman with Europo-Mongoloid features.

**Fig 3:**
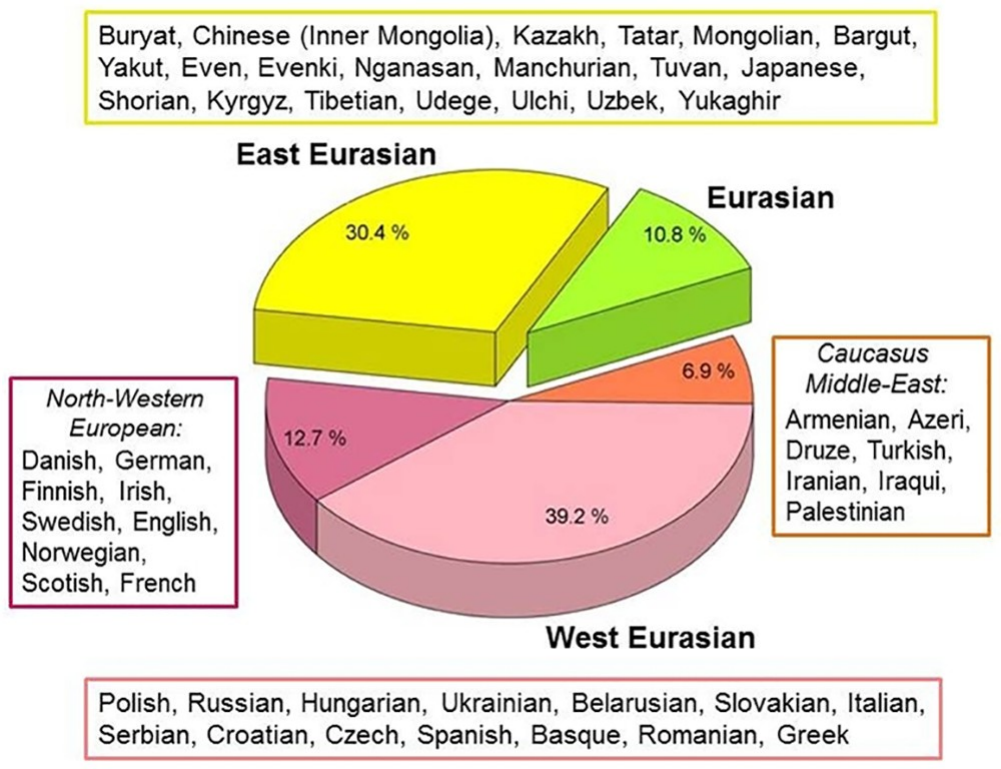
Phylogeographic origin of the 102 Conqueror maternal lineages. Data are summarized from S1 Fig. Origin of modern individuals with closest matches to Conqueror sequences are listed next to the indicated regions, ordered according to the frequency of appearances.

**Fig 4:**
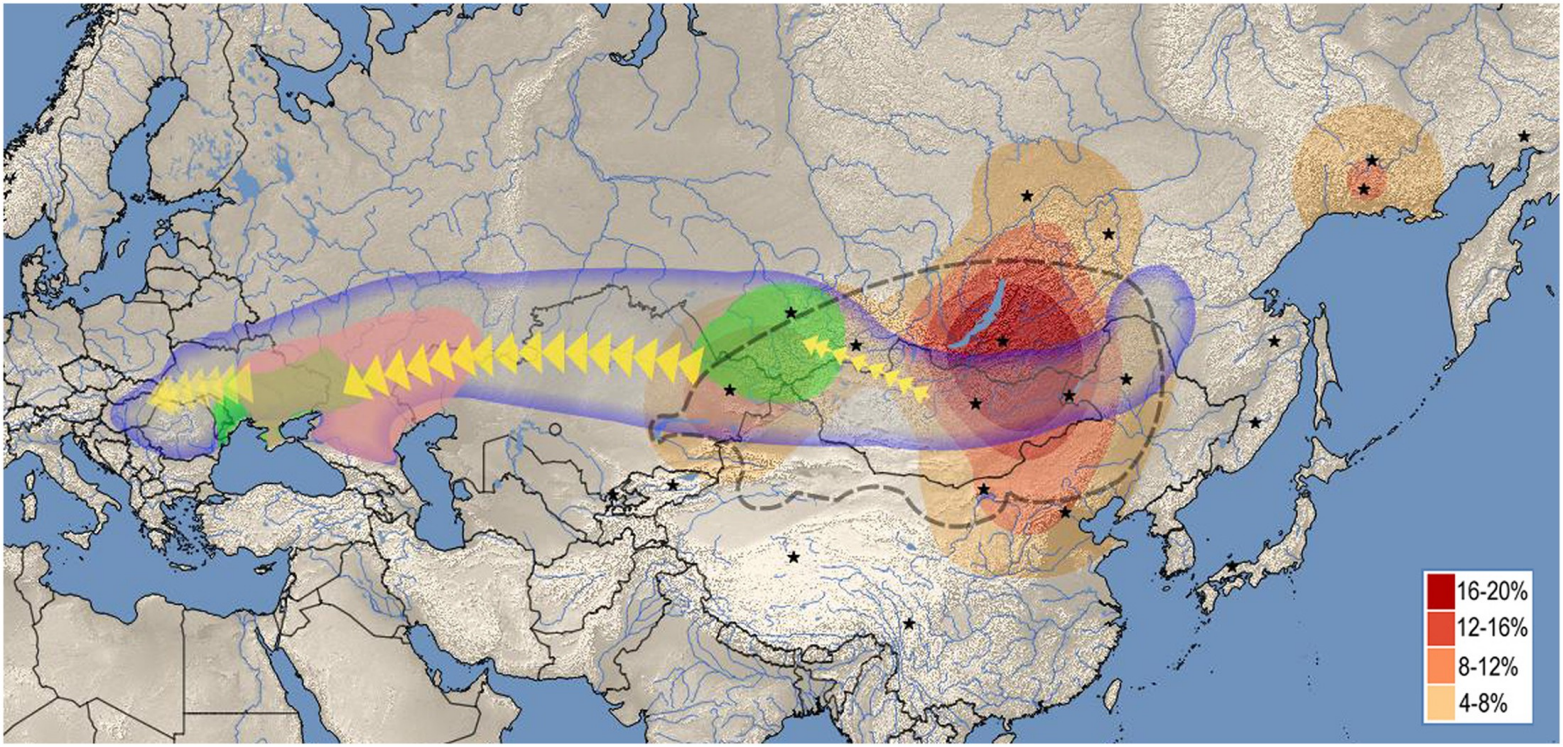
The most feasible origin and migration route of different components of the Hungarian Conquerors based on this study. Red heat map displays the geographic distribution of closest East Eurasian sequence matches to individual Conqueror samples. Stars denote geolocations of East Eurasian ethnic groups listed on S1 Fig (summarized on S1A Table), map was drawn from their frequency of occurence. Heat map designate the area from which the East Eurasian lineages most likely originated, well corresponding to the range of the ancient Xiongnu Empire outlined by dashed line. Areas where Asian and European Scythian remains were found are labeled green. Asian Scythians around Tuva correspond to the most probable sources of Eurasian lineages. Pink label shows the presumptive range of the Srubnaya culture, from where European lineages were most likely derived. Bluish line frames the Eurasian steppe zone, within which all presumptive ancestors of the Conquerors were found. The map was created using QGIS 2.18.4[51].

**Fig 5:**
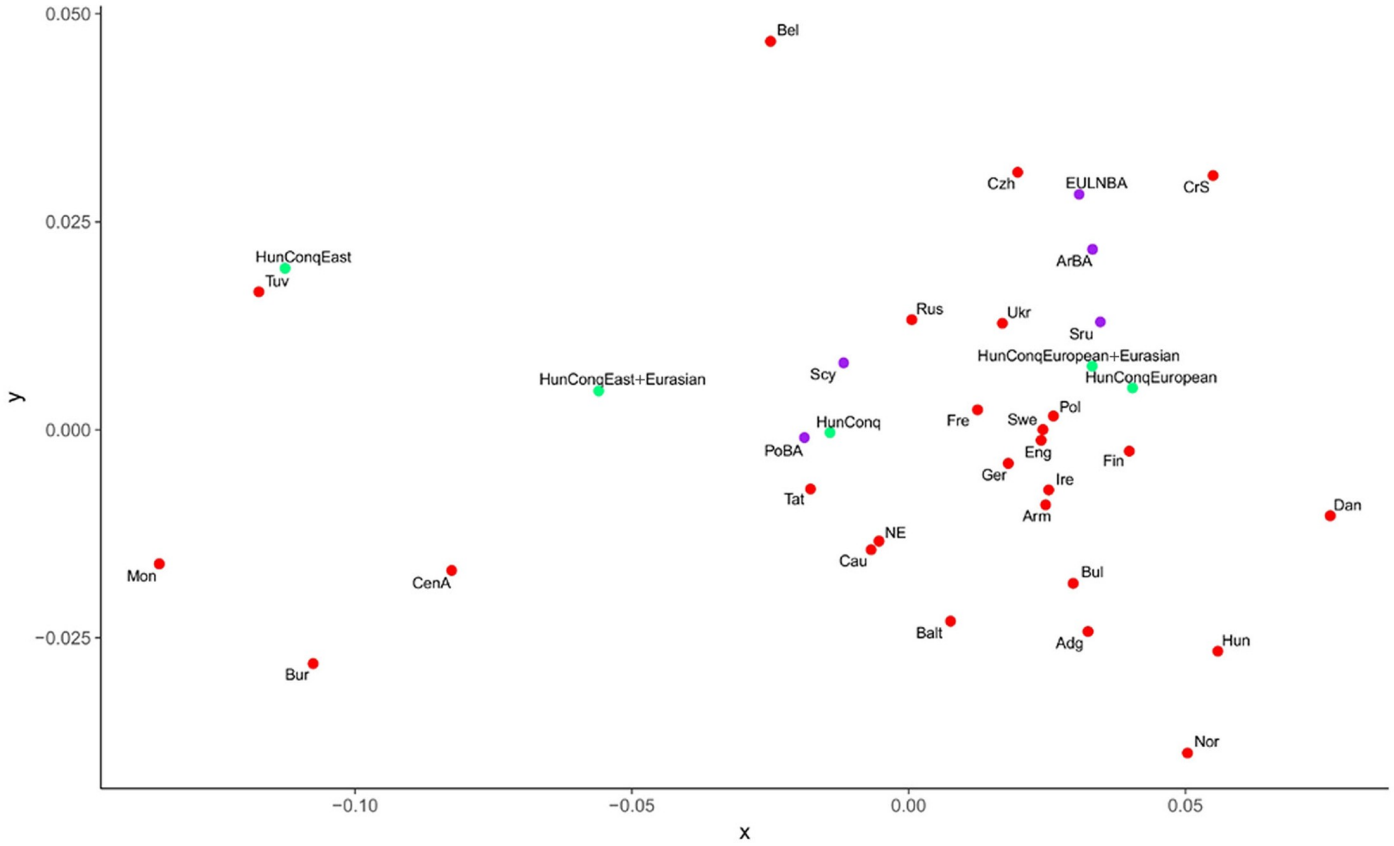
MDS plot from linearized Slatkin Fst values of S4A Table. Only populations from Table 1 were depicted, which showed close Fst and SHD distance values to the Conquerors. Abbreviations of population names are given in S3B Table.

**Fig 6:**
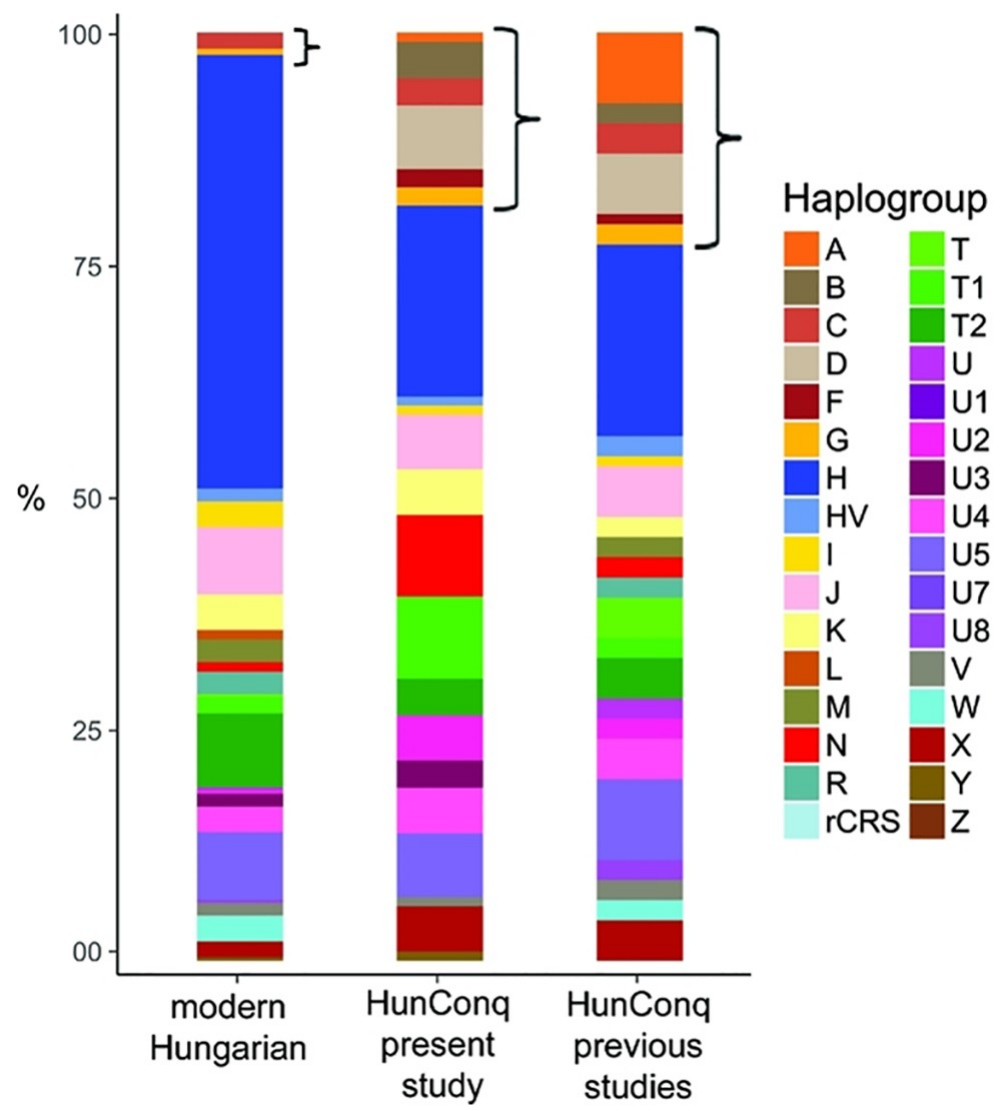
Comparison of major Hg distributions from modern and ancient Hungarian populations. Asian main Hg-s are designated with brackets. Major Hg distribution of Conqueror samples from this study are very similar to that of other 91 Conquerors taken from previous studies [11,12]. Modern Hungarians have very small Asian components pointing at small contribution from the Conquerors. Of the 289 modern Hungarian mitogenomes 272 are newly deposited [29].
