# Supplementary material for: Mitogenomic data indicate admixture components of Central-Inner Asian and Srubnaya origin in the conquering Hungarians
Source: PLoS One. 2018 Oct 18;13(10):e0205920. doi: 10.1371/journal.pone.0205920 (PMC6193700; doi:10.1371/journal.pone.0205920)
Supplement: S1 Appendix — (DOCX) [file pone.0205920.s001.docx]

**S1 Appendix**

**Basic description of the studied cemeteries.**

(Detailed archaeological and anthropological description of each graves is provided in S1a Table.)

**1., Karos-Eperjesszög I-II-III**

A more detailed description of the three Karos cemeteries is also given in [1]. The site of Karos-Eperjesszög is located in Northeast Hungary, in the so-called Bodrogköz (Borsod-Abaúj-Zemplén County). In the first half of the 10th century AD, this area served as the palatial center and burial place of the conquering Hungarians. The three cemeteries are situated on low sandhills that are approximately 200 meters from each other.

*Karos-Eperjesszög I:* Contained approximately fifty burials, most of which were destroyed during agricultural works before Tibor Horváth could excavate thirteen graves in 1936.

*Karos-Eperjesszög II:* This second cemetery was found on the center sandhill. A total of seventy-three graves were uncovered by László Révész between 1986 and 1988 [2].

*Karos-Eperjesszög III:* This third cemetery is south of Karos-Eperjesszög II and was excavated between 1988 and 1990 [2]. A total of nineteen graves were uncovered by László Révész and Mária Wolf. Dated with ^14^C, coins and findings the three Karos cemeteries were used from the last decade of the 9th century AD until the mid-10th century AD. The high number of male burials, weapons and insignia of rank indicates that the welthiest graves must have belonged to the leaders of the princely retinue [3] but the the ratio of children and elderly individuals (S1a Table) indicate a vital community. The archaeological remains of all three Karos cemeteries were examined and published in [2], and the human skeletons were studied by [4].

**2., Kenézlő****-Fazekaszug I-II**

The site is located close to Karos in Borsod-Abaúj-Zemplén County, along the river Tisza. The site was excavated in 1914 by András Jósa [5] and in 1927 by Nándor Fettich [6]. A total of fifty graves have been uncovered, all of which are dated to the Hungarian Conquest Period (10th century AD). The cemetery was used in the early 10th century AD probably by the military retinue and their families and servants. Anthropological examination of the skeletons was carried out by Lajos Bartucz [7], András Bíró and Erzsébet Fóthi [8]. Most of the skulls display europo-mongolid characteristics.

**3., Harta-Freifelt**

The site is situated one kilometer from Harta (in Bács-Kiskun County) in the field called Freifelt. The joint excavation was carried out in 2002 by Rozália Kustár and Péter Langó (Kalocsa Museum and the Archaeological Institute of HAS) [9]. A total of twenty-two undisturbed graves were discovered, dating to the first half of the 10th century AD. Archeological and anthropological analyses have not been published yet.

**4., Magyarhomoróg-Kónya-domb**

The cemetery is located at the northern border of the Magyarhomoróg village (in Hajdú-Bihar County). A total of five hundred and forty graves were excavated by István Dienes and László Kovács [10], which were dated between the 10th and 12th centuries AD.

**5., Orosháza-Görbics tanya**

The cemetery is located on the outskirts of Orosháza (in Békés County). Three graves were excavated in 1961 by István Dienes, which were dated to the 10th century AD. Anthropological analysis was performed by Gyula Farkas and Pál Lipták [11].

**6., Szabadkígyós-Pálliget**

The southern-Hungarian site of Szabadkígyós-Pálliget is located in Békés County. Seventeen graves from the 10th century AD were discovered in 1968 by Irén Juhász and Csanád Bálint. The archaeological finds were studied and published by Csanád Bálint [12], while the human remains were examined by Edit Lotterhof [13].

**7., Sárrétudvari-Hízóföld**

Two hundred and sixty two graves from the Hungarian Conquest Period were uncovered at the Hízóföld outskirts of Sárrétudvari (in Hajdú Bihar County). The site was excavated between 1983 and 1985 by the lead archaeologist, Ibolya M. Nepper [14]. The human osteological analyses were carried out by Sándor Oláh [15].

The earliest graves in the cemetery were the burials of the wealthy class, representing the first generation of the Hungarian Conquest Period. Several weapons were also excavated, which indicates that primarily the armed warriors of the nearby Bihar castle and their families were buried there. The cemetery was used until approximately 970 AD.

**8., Szegvár-Oromdűlő**

The site is located east of Szegvár (in Csongrád County). The cemetery, dating to the 10th and 11th centuries AD, was excavated from 1983 to 1996 by Gábor Lőrinczy [16]. A total of three hundred and seventy two graves were discovered, and a detailed anthropological analysis was done by Antónia Marcsik [17], while an archaeological analysis was done by Lívia Bende and Gábor Lőrinczy.

**Supplementary References**

1. Neparáczki E, Juhász Z, Pamjav H, Fehér T, Csányi B, Zink A, et al. Genetic structure of the early Hungarian conquerors inferred from mtDNA haplotypes and Y-chromosome haplogroups in a small cemetery. Molecular Genetics and Genomics. 2016: 1–14. doi:10.1007/s00438-016-1267-z

2. Révész L. A karosi honfoglaláskori temetők. Kovács L, Révész L, editors. Miskolc: Herman Ottó Múzeum és Magyar Nemzeti Múzeum; 1996.

3. Révész L. Karos-Eperjesszög, Cemeteries I-III. In: Fodor I, Révész L, Wolf M, Nepper I, editors. The Ancient Hungarians Exhibition Catalogue. Budapest: Hungarian National Museum; 1996.

4. Kustár Á. A Karos-Eperjesszögi I.-II.-III. honfoglalás kori temetők embertani vizsgálata. In: Kovács L, Révész L, editors. Magyarország honfoglalás kori és kora Árpád-kori sírleletei 1. Miskolc: A Herman Ottó Múzeum és a Magyar Nemzeti Múzeum közös kiadványa; 1996. pp. 395–456.

5. Jósa A. Honfoglalás kori emlékek Szabolcsban. Archaeológiai Értesítő. 1914;34: 303–340.

6. Fettich N. Adatok a honfoglalás kor archaeológiájához. Archaeológiai Értesítő. 1931;45: 48–112.

7. Bartucz L. Adatok a honfoglaló magyarok anthropológiájához. Archaeológiai Értesítő. 1931;45: 113–119.

8. Bíró A, Fóthi E. A Kenézlő-Fazekaszug I-II. honfoglalás kori temetők embertani vizsgálata. In: Korsós Z, editor. Kárpát-medencei Biológiai Szimpózium Előadások összefoglalói. Budapest; 2005. pp. 51–55.

9. Kustár R, Langó P. Ezüstbe öltözött lányok. Honfoglalás kori sírok Harta határában. Romsics I, editor. Kalocsa: Kalocsai Múzeumi Kiskönyvtár; 2003.

10. Kovács L. Előzetesen a magyarhomorog-kónya-dombi 10–12., századi temetőről. In: Kovács L, Révész L, editors. Népek és kultúrák a Kárpát-medencében Tanulmányok Mesterházy Károly tiszteletére. Budapest–Debrecen–Szeged; 2016. pp. 481–501.

11. Farkas G, Lipták P. Adatok Orosháza X-XII. századi népességének embertani ismeretéhez. In: Nagy G, editor. Orosháza története I. Orosháza; 1965. pp. 204–220.

12. Bálint C. X. századi temető a szabadkígyós-pálligeti táblában. A Békés Megyei Múzeumok Közleményei. 1971;1: 49–86.

13. Lotterhof E. A Szabadkígyóson feltárt X. századi temetők embertani vizsgálata. Békés Megyei Múzeumok Közleményei. 1971;1: 89–101.

14. Nepper I. Hajdú-Bihar megye 10–11. századi sírleletei. Budapest–Debrecen; 2002.

15. Oláh S. Sárrétudvari-Hízóföld honfoglalás kori temetőjének történeti embertani értékelése. University of Szeged. 1990.

16. Bende L, Lőrinczy G. A szegvár-Oromdűlő 10. és 11. századi embertani leleteinek vizsgálata. A Móra Ferenc Múzeum Évkönyve Stud Archaeol. 1997;3: 201–285.

17. Marcsik A. Szegvár-Oromdűlő 10-11. századi temető. A Móra Ferenc Múzeum Évkönyve Stud Archaeol. 1997;3: 287–322.
