# Supplementary material for: Mitogenomic data indicate admixture components of Central-Inner Asian and Srubnaya origin in the conquering Hungarians
Source: PLoS One. 2018 Oct 18;13(10):e0205920. doi: 10.1371/journal.pone.0205920 (PMC6193700; doi:10.1371/journal.pone.0205920)
Supplement: S1 Fig — Phylogenetic trees are arranged in alphabetic order according to haplogroups. The 67 sub-haplogroups are depicted on 58 Networks. Samples falling into the same sub-haplogroup with the studied sample are encircled. The smallest colored circles represent one individual; circle sizes are proportional to the number of individuals with identical sequences. (When large number of sequences with few phylogenetically informative SNP-s are aligned, the algorithm may force the most similar but not identical sequences into the same large circle.) Green circles identify Hungarian conqueror samples, red circles represent modern samples, and violet circles correspond to ancient samples. A few ancient samples belonging to the shown haplogroup could not be properly aligned due to incomplete sequences, and these were connected with dashed line to the tree. Number of crosslines between neighboring circles denotes mutation distances. Length of connecting lines is irrelevant, as they were modified in order to fit page. Genebank accesion number and origin of samples closest to the studied conquerors are listed next to the circles. Known Conqueror Y-chromosome haplogroups were added in blue color. We summarized the probable origin of the samples’ Hg lineage in colored framed text. In some cases comments are given next to the trees. (PDF) [file pone.0205920.s002.pdf]

# S1 Figure: Median-Joining Networks (1-58) for mtDNA sequences of the 102 Hungarian Conquerors

Phylogenetic trees are arranged in alphabetic order according to haplogroups. The 67 sub-haplogroups are depicted on 58 Networks. Samples falling into the same sub-haplogroup with the studied sample are encircled.

The smallest colored circles represent one individual; circle sizes are proportional to the number of individuals with identical sequences. (When large number of sequences with few phylogenetically informative SNP-s are aligned, the algorithm may force the most similar but not identical sequences into the same large circle.) Green circles identify Hungarian conqueror samples, red circles represent modern samples, and violet circles correspond to ancient samples. A few ancient samples belonging to the shown haplogroup could not be properly aligned due to incomplete sequences, and these were connected with dashed line to the tree.

Number of crosslines between neighboring circles denotes mutation distances. Length of connecting lines is irrelevant, as they were modified in order to fit page. Genebank accesion number and origin of samples closest to the studied conquerors are listed next to the circles. Known Conqueror Y-chromosome haplogroups were added in blue color.

We summarized the probable origin of the samples’ Hg lineage in colored framed text. In some cases comments are given next to the trees.

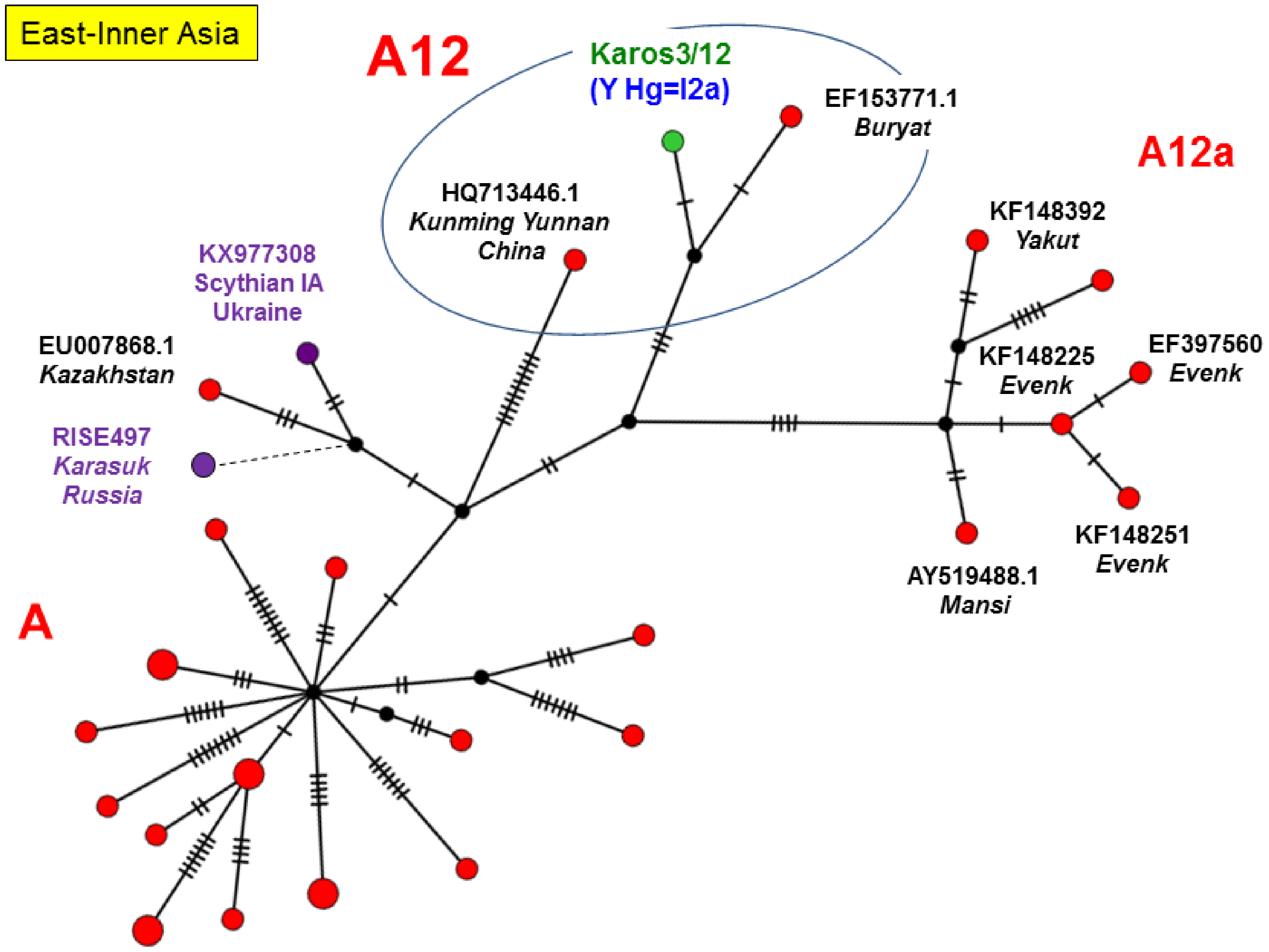
East-Inner Asia
A12
Karos3/12
(Y Hg=I2a)
EF153771.1
Buryat
A12a
HQ713446.1
Kunming Yunnan
China
KF148392
Yakut
KX977308
Scythian IA
Ukraine
EU007868.1
Kazakhstan
KF148225
Evenk
EF397560
Evenk
RISE497
Karasuk
Russia
KF148251
Evenk
AY519488.1
Mansi
A

**Network 1.**

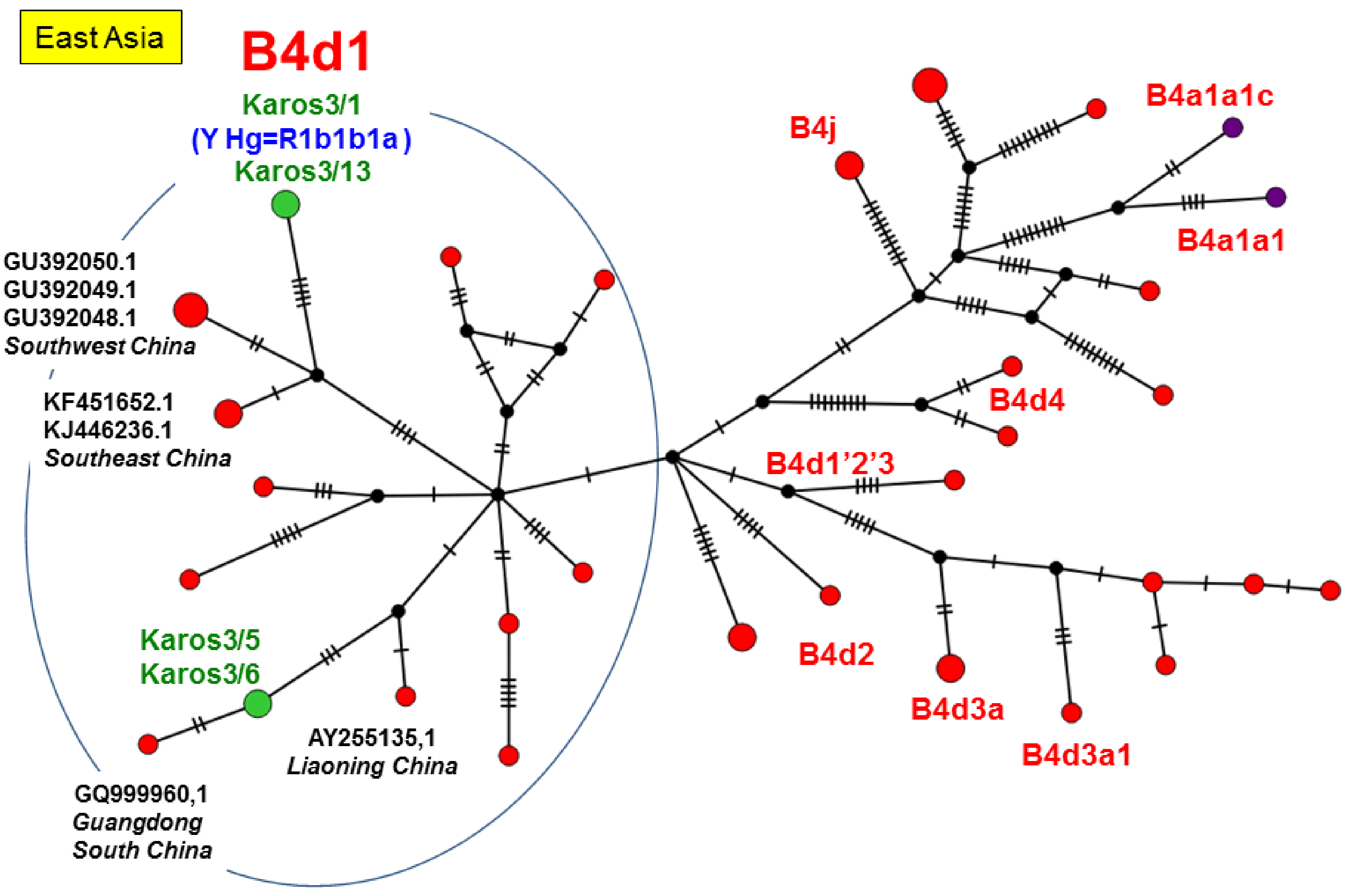
East Asia
B4d1
Karos3/1
(Y Hg=R1b1b1a )
Karos3/13
GU392050.1
GU392049.1
GU392048.1
Southwest China
KF451652.1
KJ446236.1
Southeast China
Karos3/5
Karos3/6
AY255135,1
Liaoning China
GQ999960,1
Guangdong
South China
B4j
B4a1a1c
B4a1a1
B4d4
B4d1'2'3
B4d2
B4d3a
B4d3a1

Network 2.

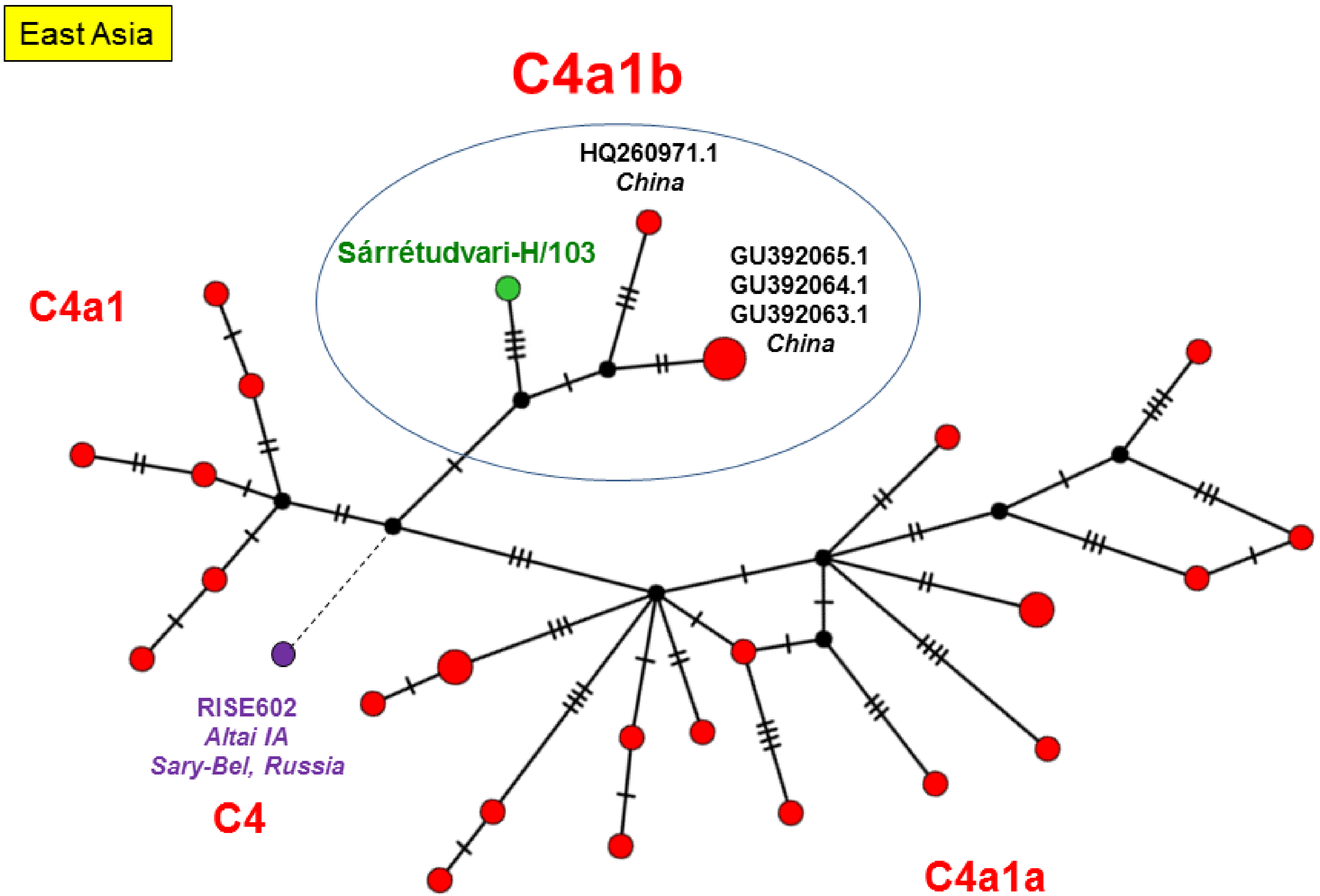
East Asia
C4a1b
HQ260971.1
China
Sárrétudvari-H/103
GU392065.1
GU392064.1
GU392063.1
China
C4a1
RISE602
Altai IA
Sary-Bel, Russia
C4
C4a1a

**Network 3.**

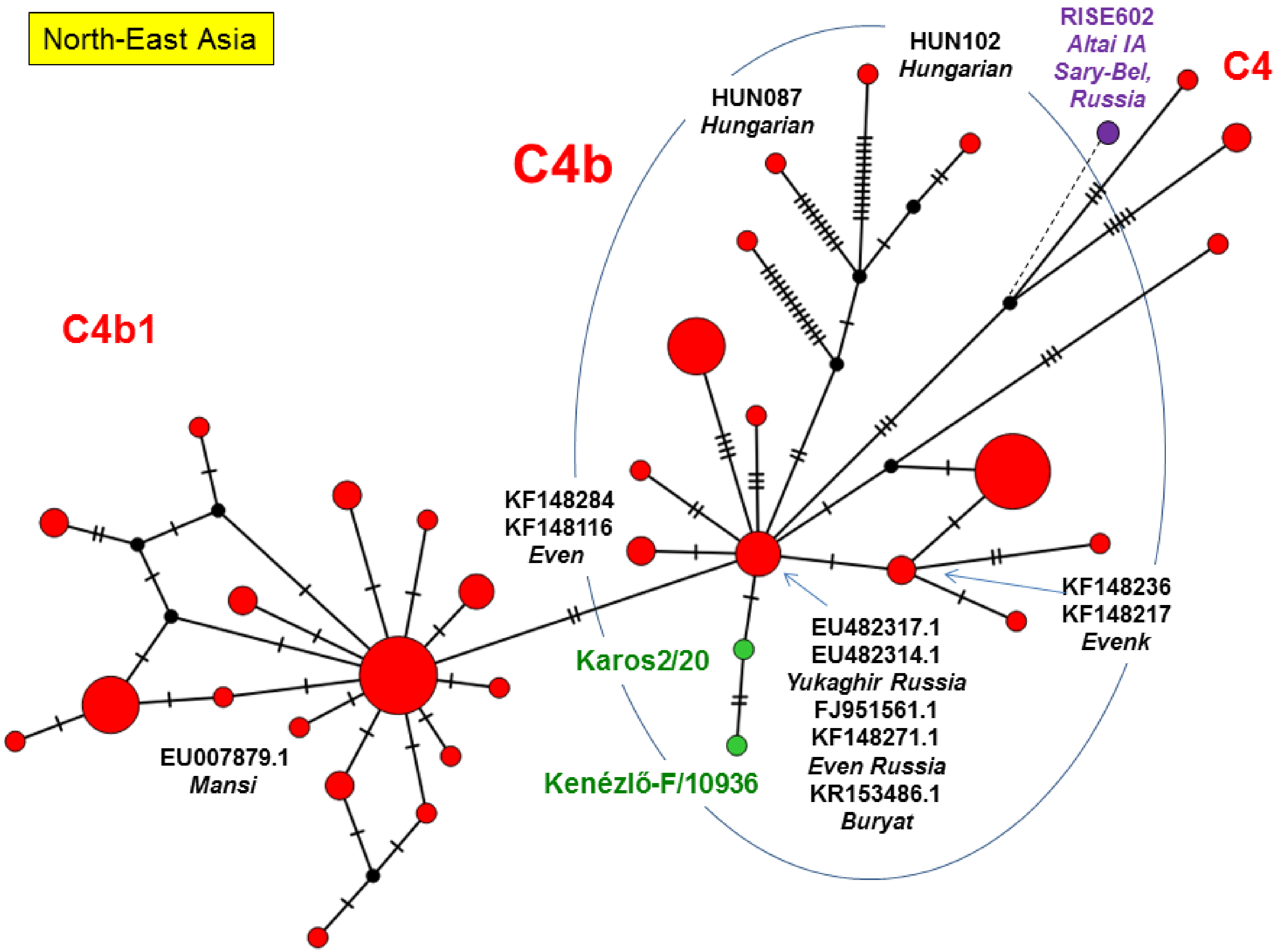
North-East Asia
C4b1
C4b
C4
HUN087
Hungarian
HUN102
Hungarian
RISE602
Altai IA
Sary-Bel,
Russia
KF148284
KF148116
Even
Karos2/20
Kenézlő-F/10936
EU482317.1
EU482314.1
Yukaghir Russia
FJ951561.1
KF148271.1
Even Russia
KR153486.1
Buryat
KF148236
KF148217
Evenk
EU007879.1
Mansi

**Network 4.**

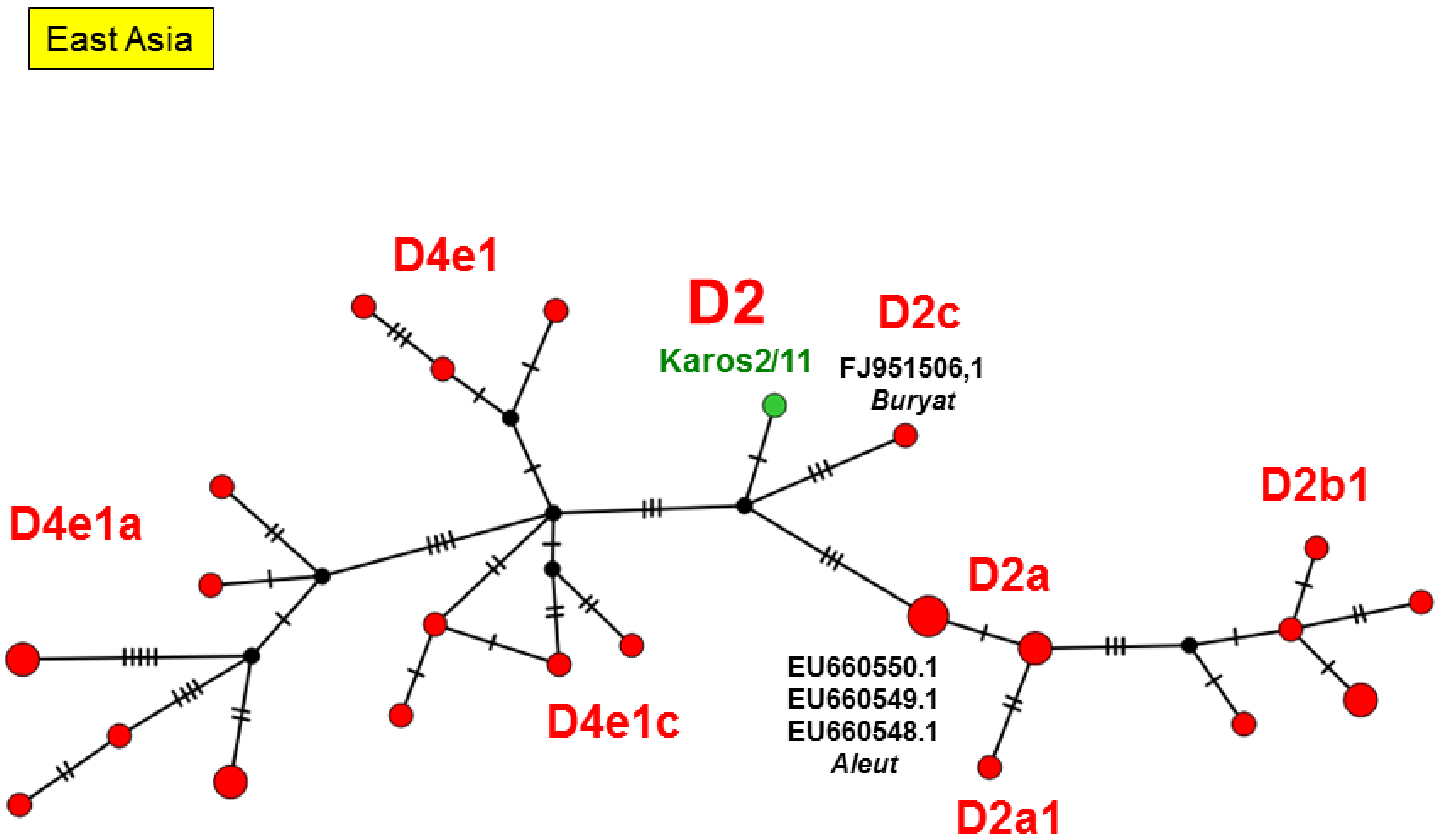
East Asia
D4e1
D2
D2c
Karos2/11
FJ951506,1
Buryat
D4e1a
D2b1
D2a
D4e1c
EU660550.1
EU660549.1
EU660548.1
Aleut
D2a1

Network 5.

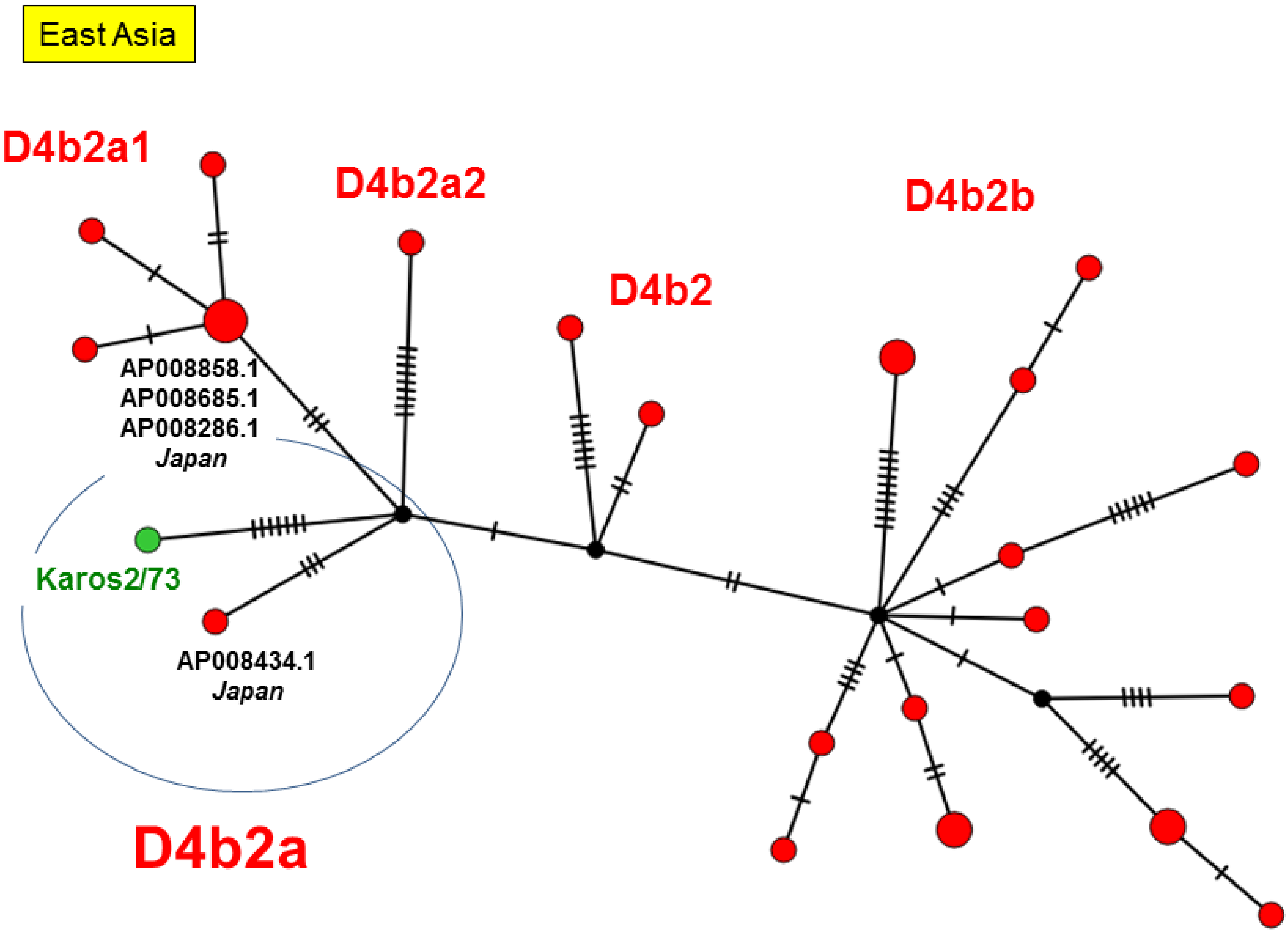
East Asia
D4b2a1
D4b2a2
D4b2
D4b2b
AP008858.1
AP008685.1
AP008286.1
Japan
Karos2/73
AP008434.1
Japan
D4b2a

**Network 6.**

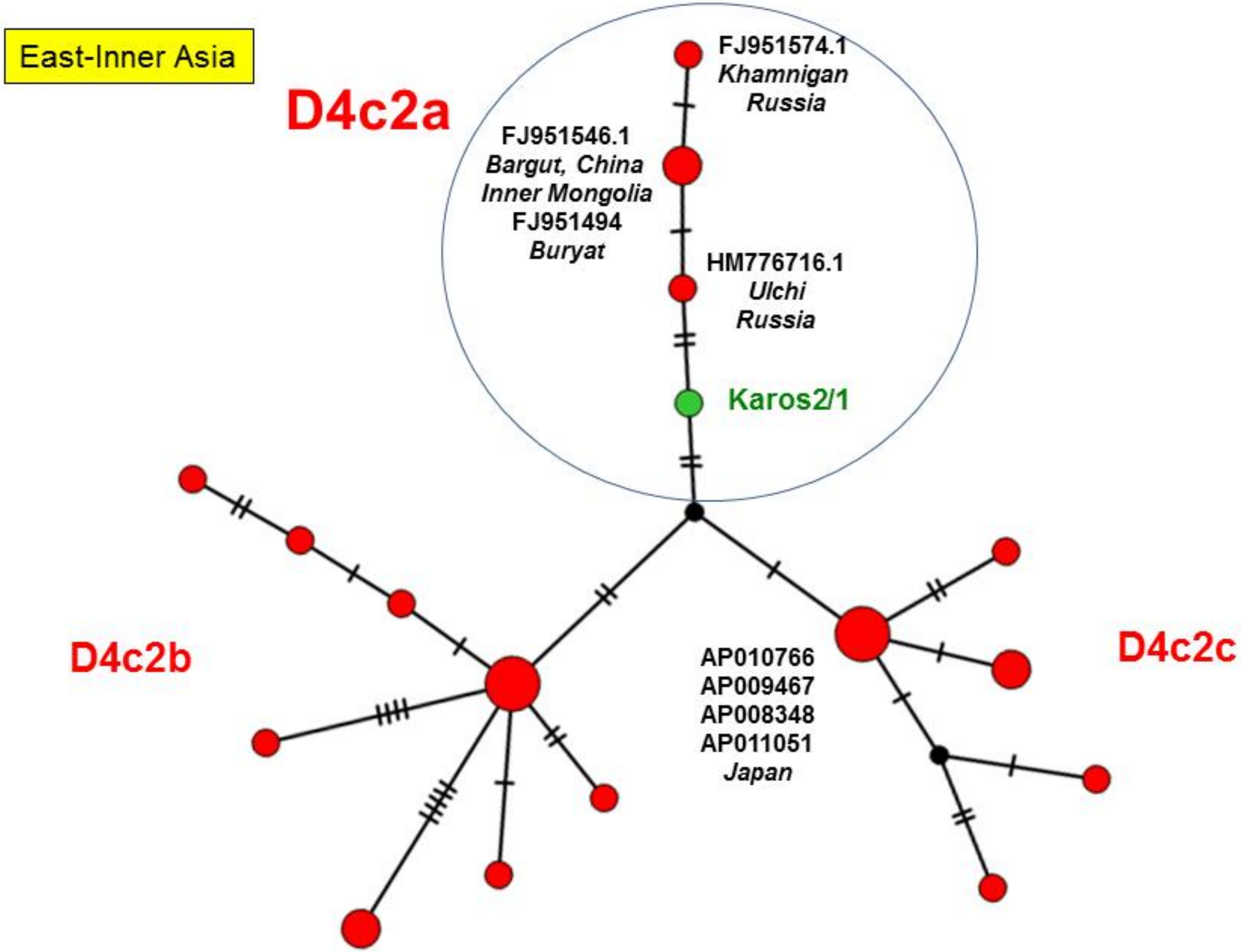
East-Inner Asia
D4c2a
FJ951574.1
Khamnigan
Russia
FJ951546.1
Bargut, China
Inner Mongolia
FJ951494
Buryat
HM776716.1
Ulchi
Russia
Karos2/1
D4c2b
D4c2c
AP010766
AP009467
AP008348
AP011051
Japan

**Network 7.**

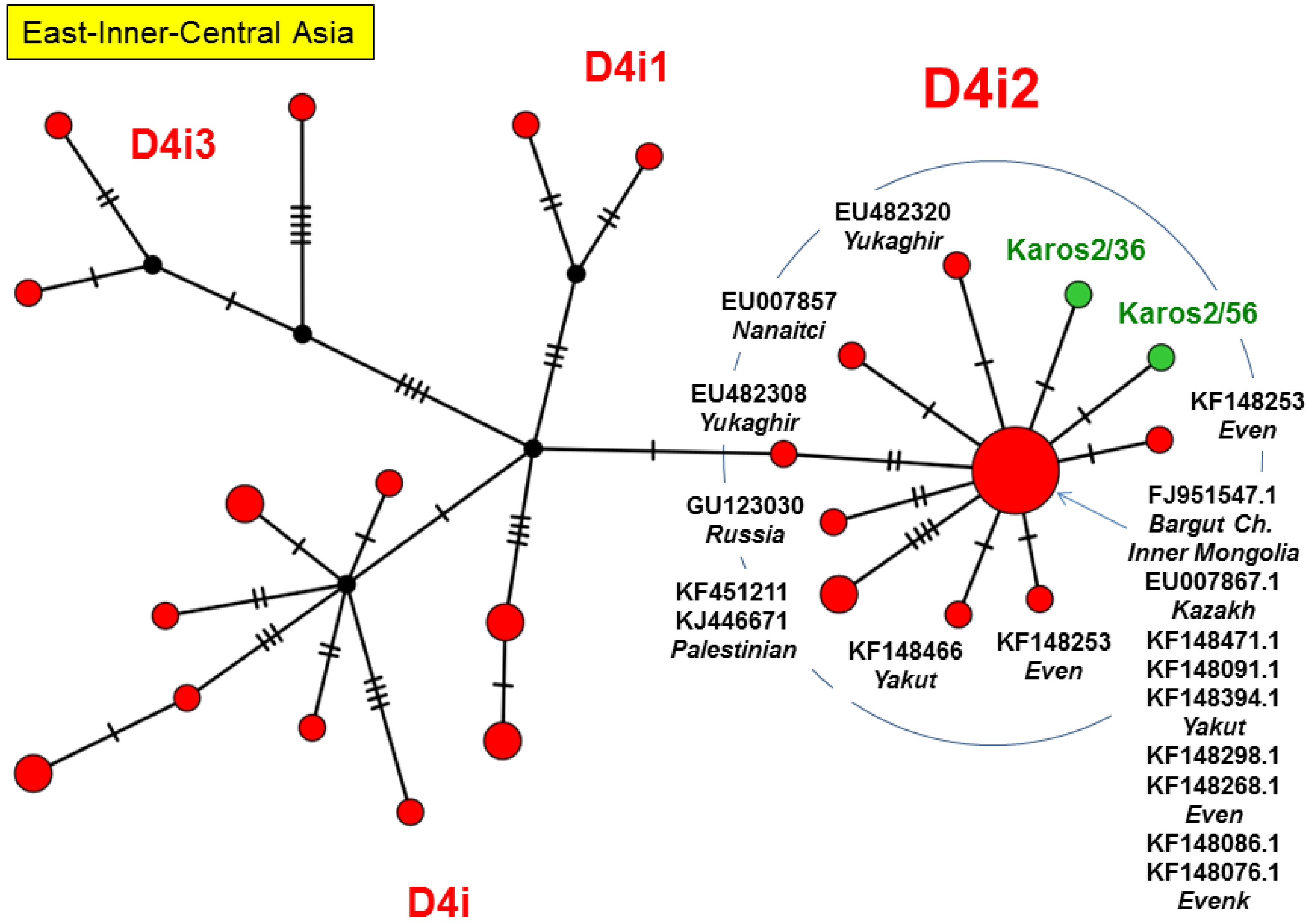
East-Inner-Central Asia
D4i3
D4i1
D4i2
D4i
EU482320
Yukaghir
Karos2/36
Karos2/56
EU007857
Nanaitci
EU482308
Yukaghir
KF148253
Even
GU123030
Russia
KF451211
KJ446671
Palestinian
KF148466
Yakut
KF148253
Even
FJ951547.1
Bargut Ch.
Inner Mongolia
EU007867.1
Kazakh
KF148471.1
KF148091.1
KF148394.1
Yakut
KF148298.1
KF148268.1
Even
KF148086.1
KF148076.1
Evenk

**Network 8.**

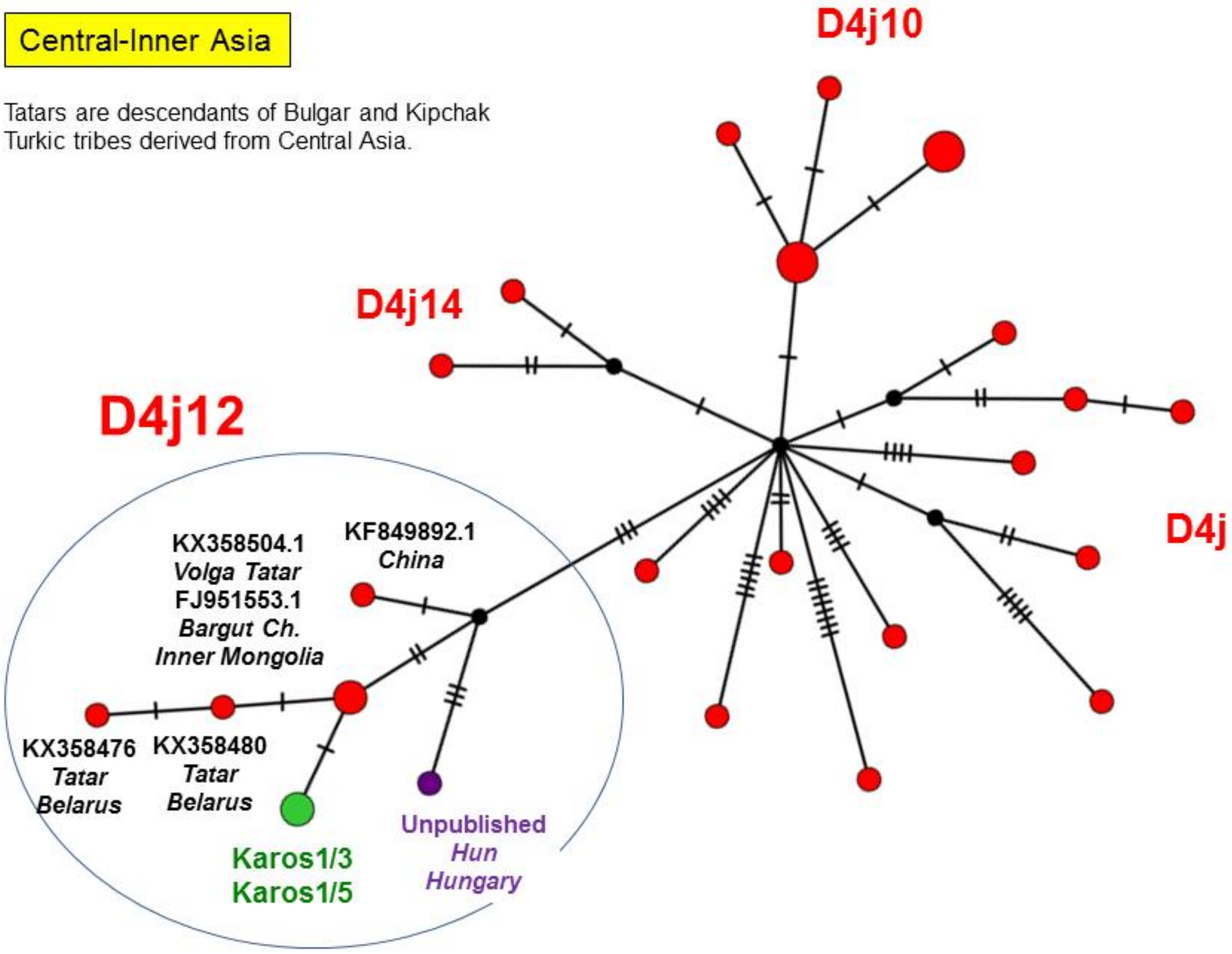

Central-Inner Asia
Tatars are descendants of Bulgar and Kipchak Turkic tribes derived from Central Asia.
D4j10
D4j14
D4j12
D4j
KX358504.1
Volga Tatar
FJ951553.1
Bargut Ch.
Inner Mongolia
KF849892.1
China
KX358476
Tatar
Belarus
KX358480
Tatar
Belarus
Karos1/3
Karos1/5
Unpublished
Hun
Hungary


**Network 9.**

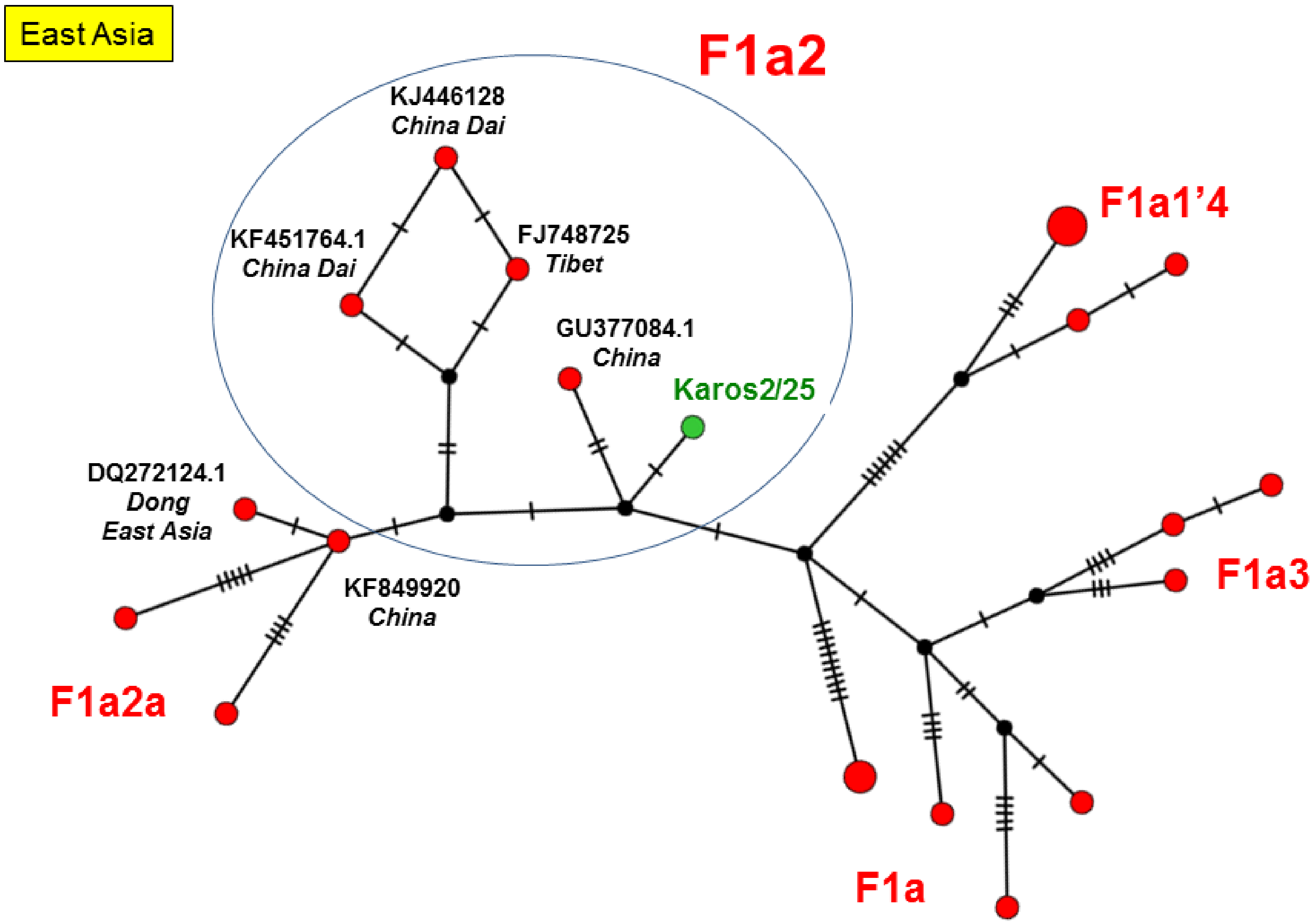
East Asia
F1a2
KJ446128
China Dai
KF451764.1
China Dai
FJ748725
Tibet
GU377084.1
China
Karos2/25
DQ272124.1
Dong
East Asia
KF849920
China
F1a2a
F1a1'4
F1a3
F1a

**Network 10.**

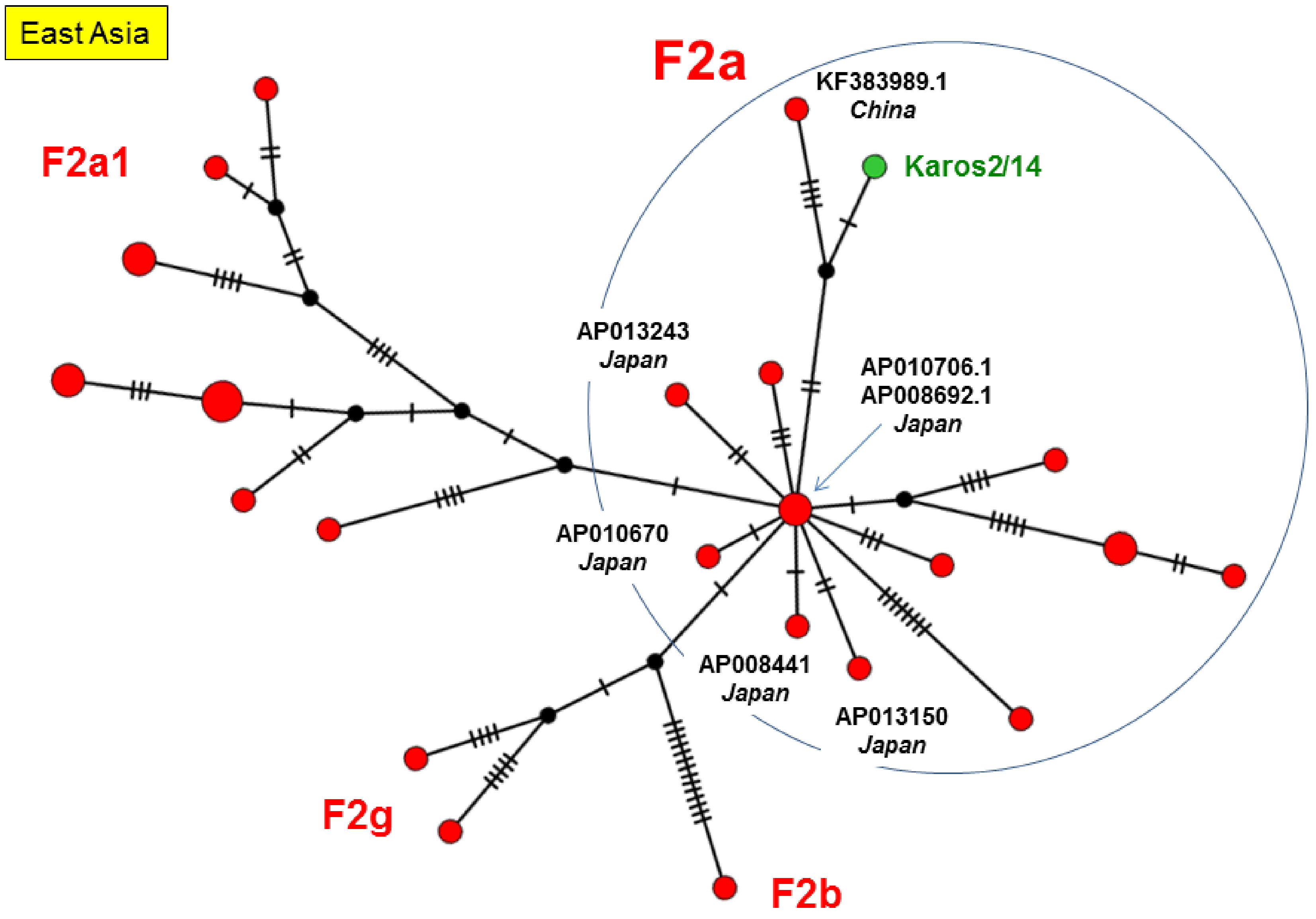

East Asia
F2a
F2a1
KF383989.1
China
Karos2/14
AP013243
Japan
AP010706.1
AP008692.1
Japan
AP010670
Japan
AP008441
Japan
AP013150
Japan
F2g
F2b


**Network 11.**

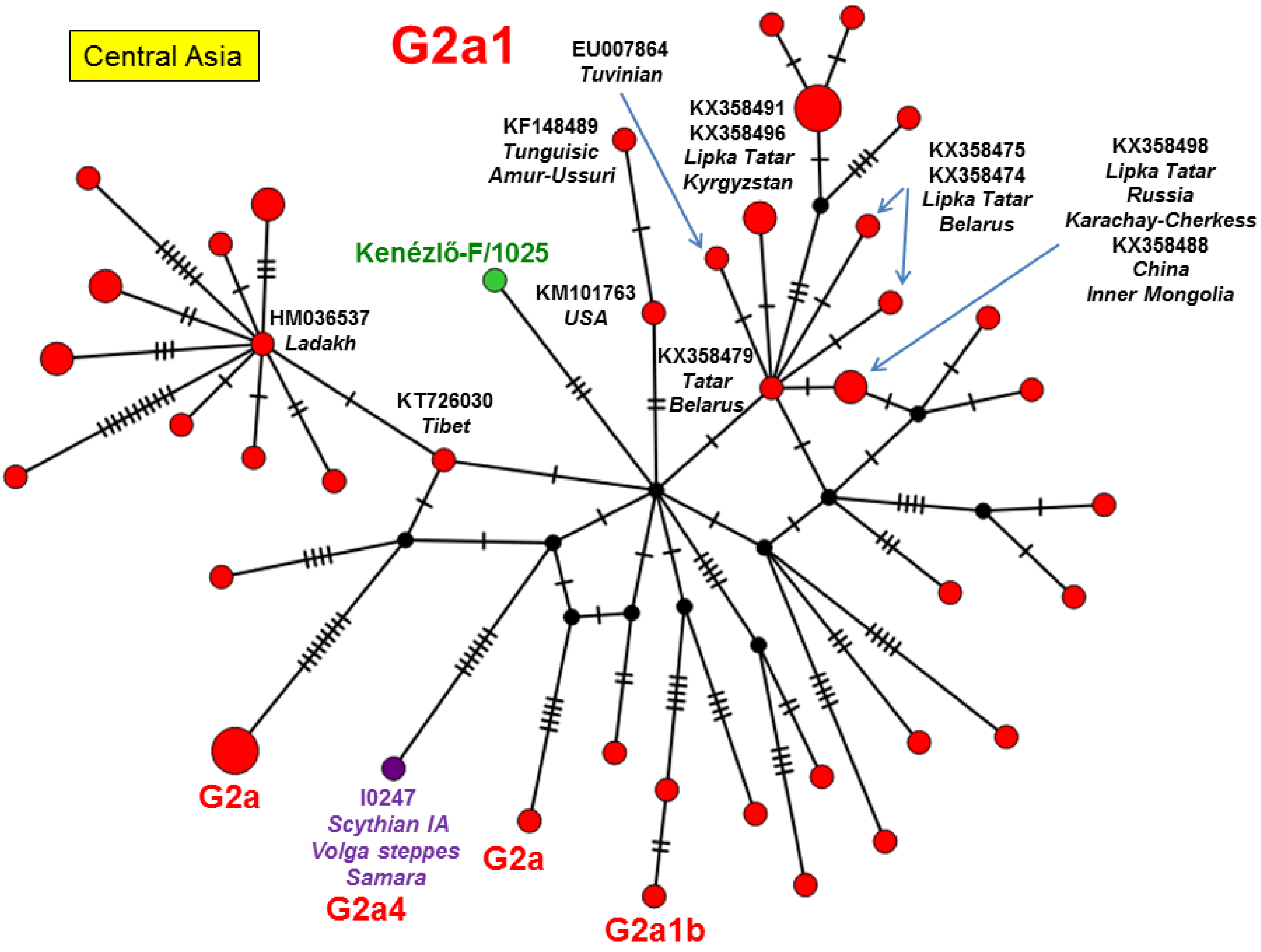
Central Asia
G2a1
EU007864
Tuvinian
KF148489
Tunguisic
Amur-Ussuri
KX358491
KX358496
Lipka Tatar
Kyrgyzstan
KX358475
KX358474
Lipka Tatar
Belarus
KX358498
Lipka Tatar
Russia
Karachay-Cherkess
KX358488
China
Inner Mongolia
Kenézlő-F/1025
KM101763
USA
HM036537
Ladakh
KX358479
Tatar
Belarus
KT726030
Tibet
G2a
I0247
Scythian IA
Volga steppes
Samara
G2a4
G2a
G2a1b

**Network 12.**

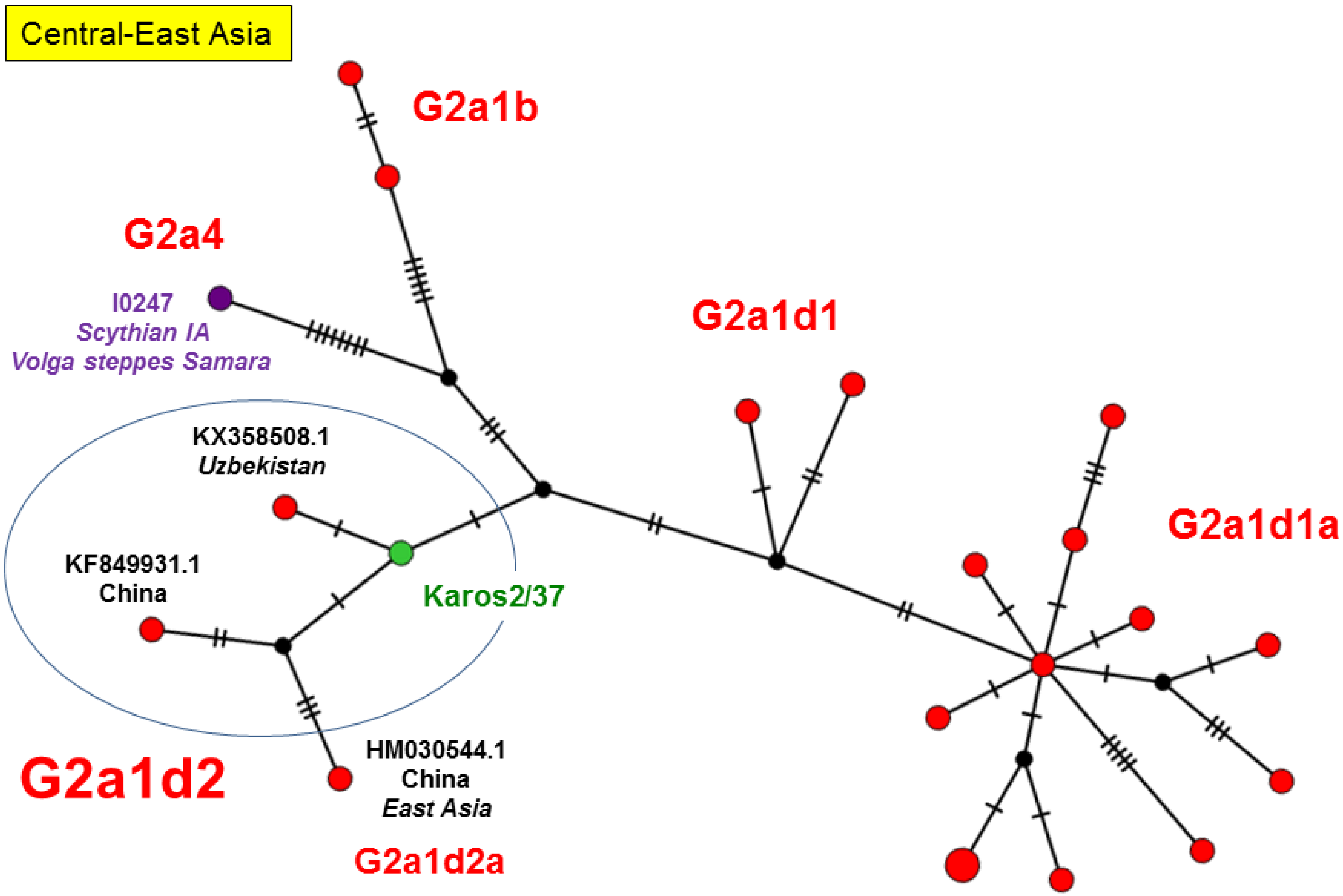
Central-East Asia
G2a1b
G2a4
I0247
Scythian IA
Volga steppes Samara
G2a1d1
KX358508.1
Uzbekistan
G2a1d1a
KF849931.1
China
Karos2/37
G2a1d2
HM030544.1
China
East Asia
G2a1d2a

**Network 13.**

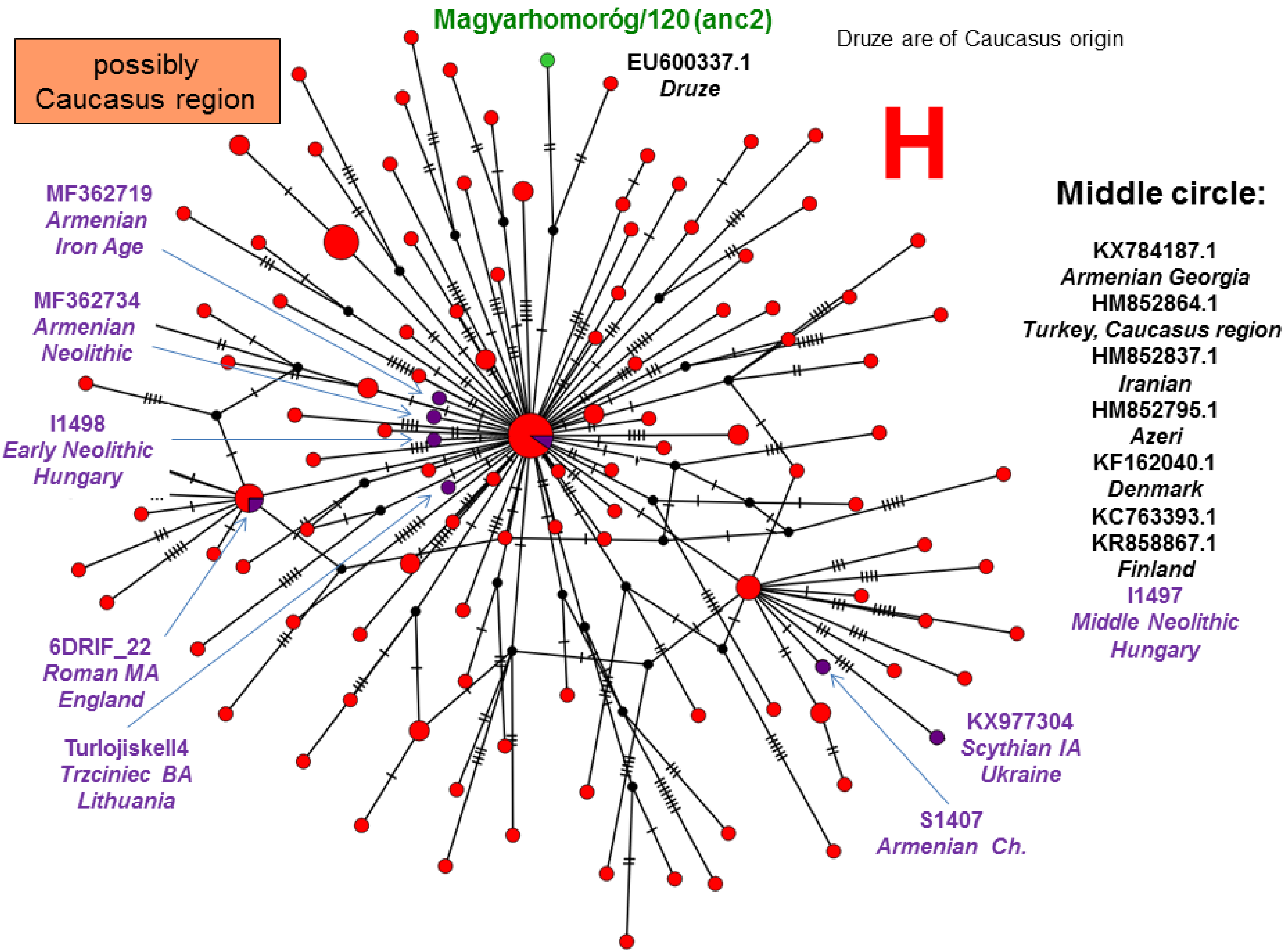
possibly
Caucasus region
Magyarhomoróg/120 (anc2)
EU600337.1
Druze
Druze are of Caucasus origin
H
Middle circle:
KX784187.1
Armenian Georgia
HM852864.1
Turkey, Caucasus region
HM852837.1
Iranian
HM852795.1
Azeri
KF162040.1
Denmark
KC763393.1
KR858867.1
Finland
I1497
Middle Neolithic
Hungary
MF362719
Armenian
Iron Age
MF362734
Armenian
Neolithic
I1498
Early Neolithic
Hungary
6DRIF_22
Roman MA
England
Turlojiskell4
Trzciniec BA
Lithuania
KX977304
Scythian IA
Ukraine
S1407
Armenian Ch.

**Network 14.**

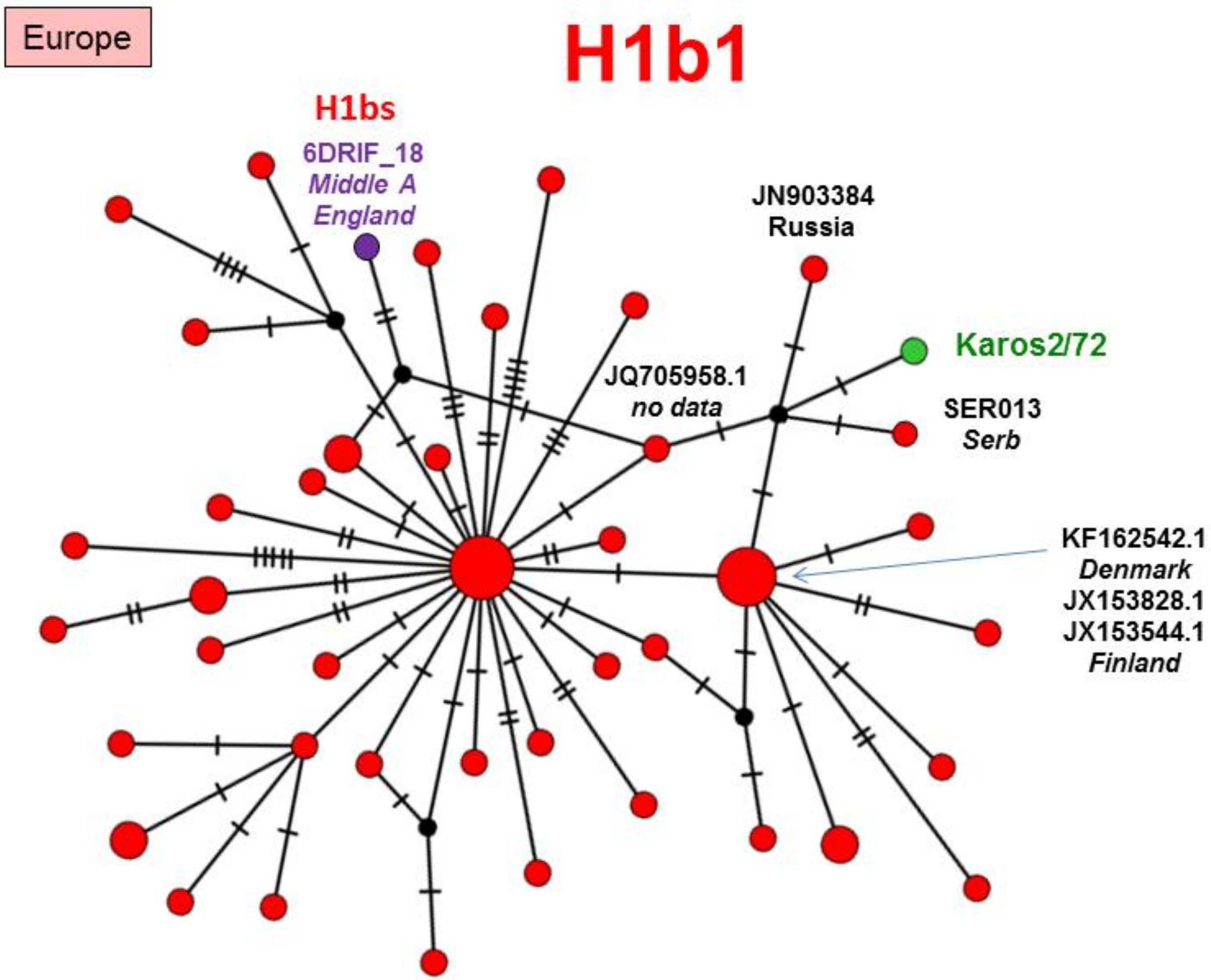
Europe
H1b1
H1bs
6DRIF_18
Middle A
England
JN903384
Russia
Karos2/72
JQ705958.1
no data
SER013
Serb
KF162542.1
Denmark
JX153828.1
JX153544.1
Finland

**Network 15.**

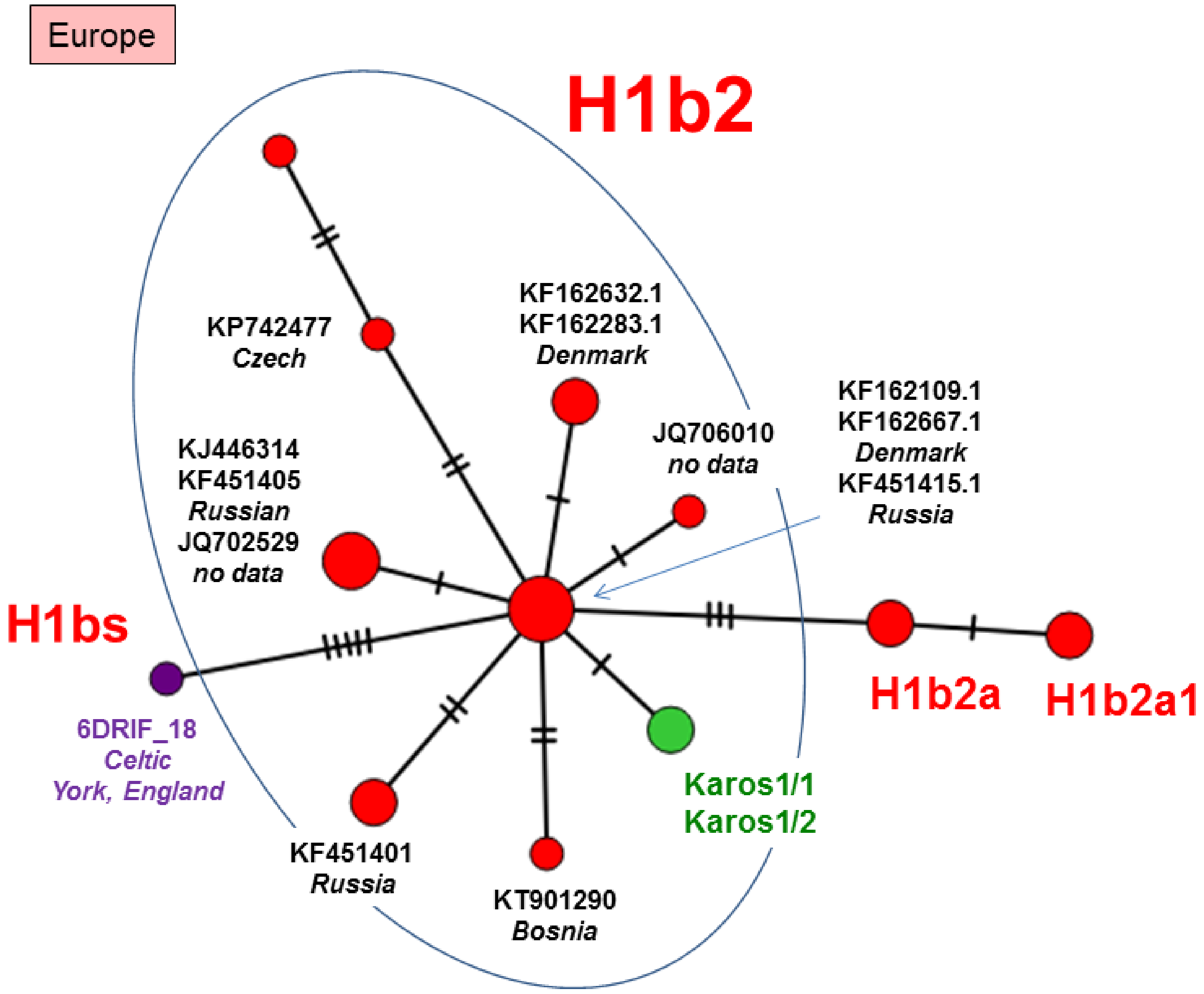
Europe
H1b2
KP742477
Czech
KF162632.1
KF162283.1
Denmark
KJ446314
KF451405
Russian
JQ702529
no data
JQ706010
no data
KF162109.1
KF162667.1
Denmark
KF451415.1
Russia
H1bs
6DRIF_18
Celtic
York, England
H1b2a
H1b2a1
Karos1/1
Karos1/2
KF451401
Russia
KT901290
Bosnia

**Network 16.**

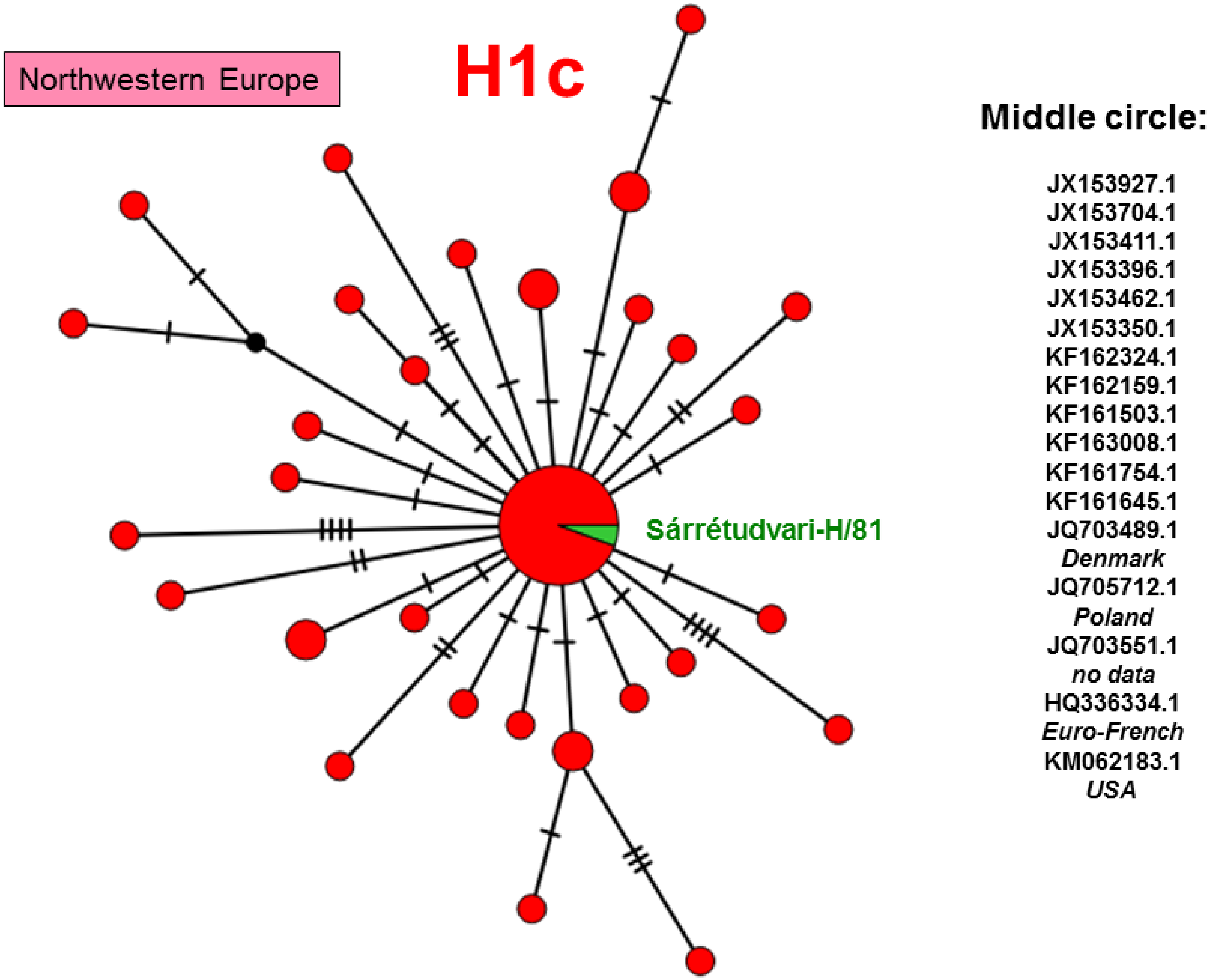
Northwestern Europe
H1c
Sárrétudvari-H/81
Middle circle:
JX153927.1
JX153704.1
JX153411.1
JX153396.1
JX153462.1
JX153350.1
KF162324.1
KF162159.1
KF161503.1
KF163008.1
KF161754.1
KF161645.1
JQ703489.1
Denmark
JQ705712.1
Poland
JQ703551.1
no data
HQ336334.1
Euro-French
KM062183.1
USA

**Network 17.**

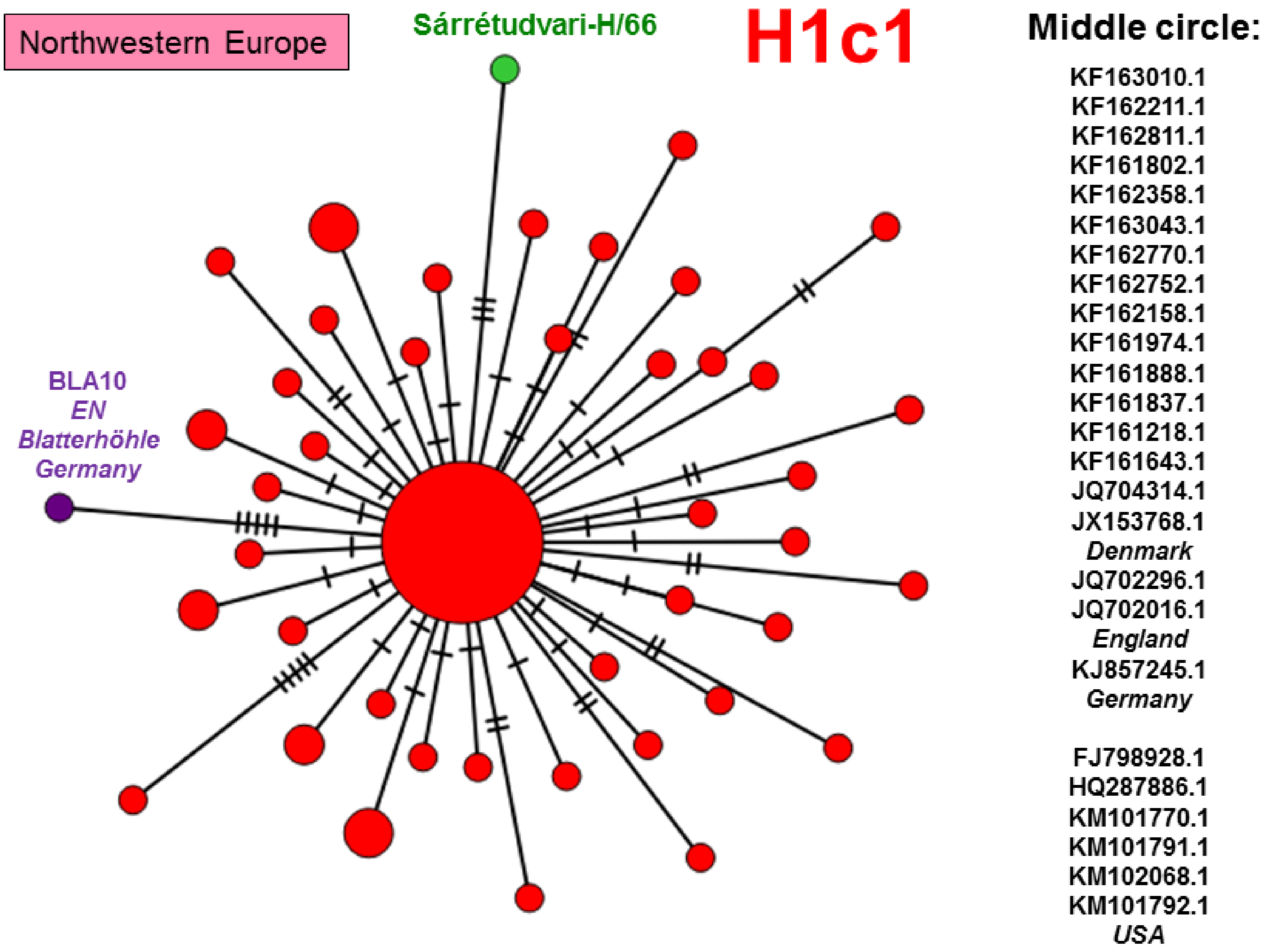
Northwestern Europe
Sárrétudvari-H/66
H1c1
BLA10
EN
Blatterhöhle
Germany
Middle circle:
KF163010.1
KF162211.1
KF162811.1
KF161802.1
KF162358.1
KF163043.1
KF162770.1
KF162752.1
KF162158.1
KF161974.1
KF161888.1
KF161837.1
KF161218.1
KF161643.1
JQ704314.1
JX153768.1
Denmark
JQ702296.1
JQ702016.1
England
KJ857245.1
Germany
FJ798928.1
HQ287886.1
KM101770.1
KM101791.1
KM102068.1
KM101792.1
USA

**Network 18.**

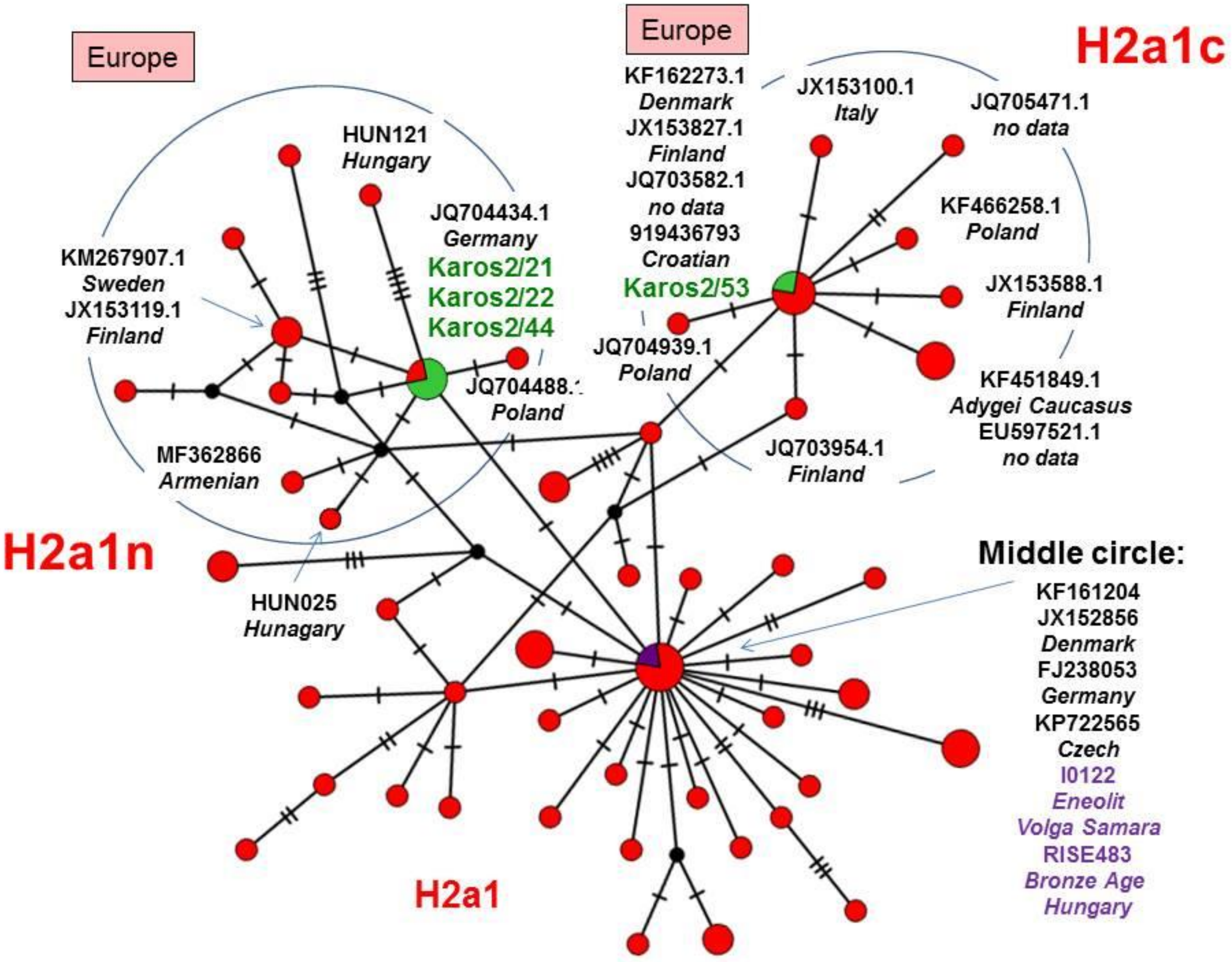

Europe
Europe
H2a1c
KF162273.1
Denmark
JX153827.1
Finland
JQ703582.1
no data
919436793
Croatian
Karos2/53
JX153100.1
Italy
JQ705471.1
no data
KF466258.1
Poland
JX153588.1
Finland
KF451849.1
Adygei Caucasus
EU597521.1
no data
JQ704939.1
Poland
JQ703954.1
Finland
HUN121
Hungary
JQ704434.1
Germany
Karos2/21
Karos2/22
Karos2/44
KM267907.1
Sweden
JX153119.1
Finland
JQ704488.1
Poland
MF362866
Armenian
H2a1n
HUN025
Hunagary
Middle circle:
KF161204
JX152856
Denmark
FJ238053
Germany
KP722565
Czech
I0122
Eneolit
Volga Samara
RISE483
Bronze Age
Hungary
H2a1


**Network 19.**

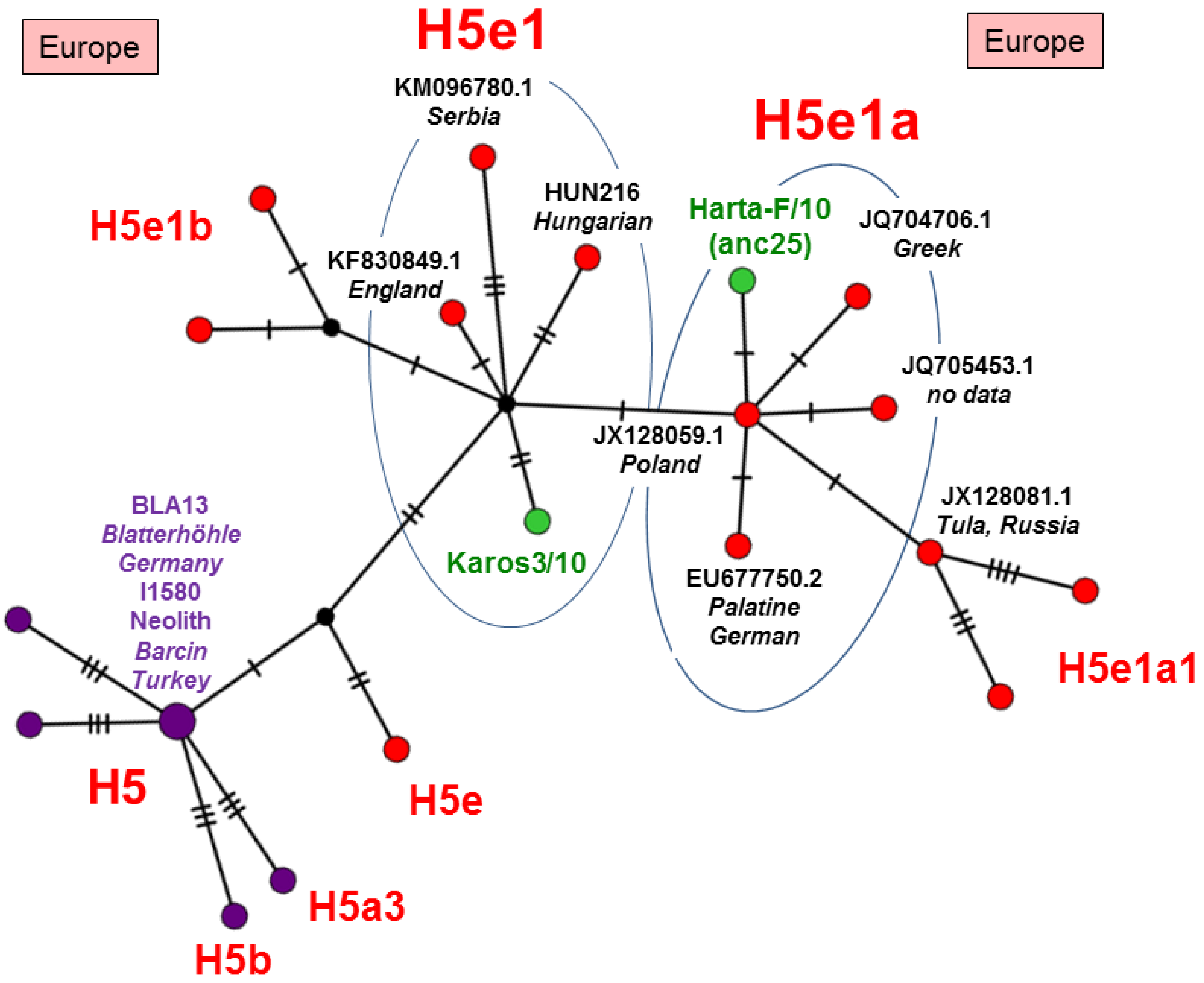
Europe
H5e1
Europe
KM096780.1
Serbia
H5e1a
HUN216
Hungarian
Harta-F/10
(anc25)
JQ704706.1
Greek
H5e1b
KF830849.1
England
JQ705453.1
no data
JX128059.1
Poland
BLA13
Blatterhöhle
Germany
I1580
Neolith
Barcin
Turkey
Karos3/10
JX128081.1
Tula, Russia
EU677750.2
Palatine
German
H5e1a1
H5
H5e
H5a3
H5b

**Network 20.**

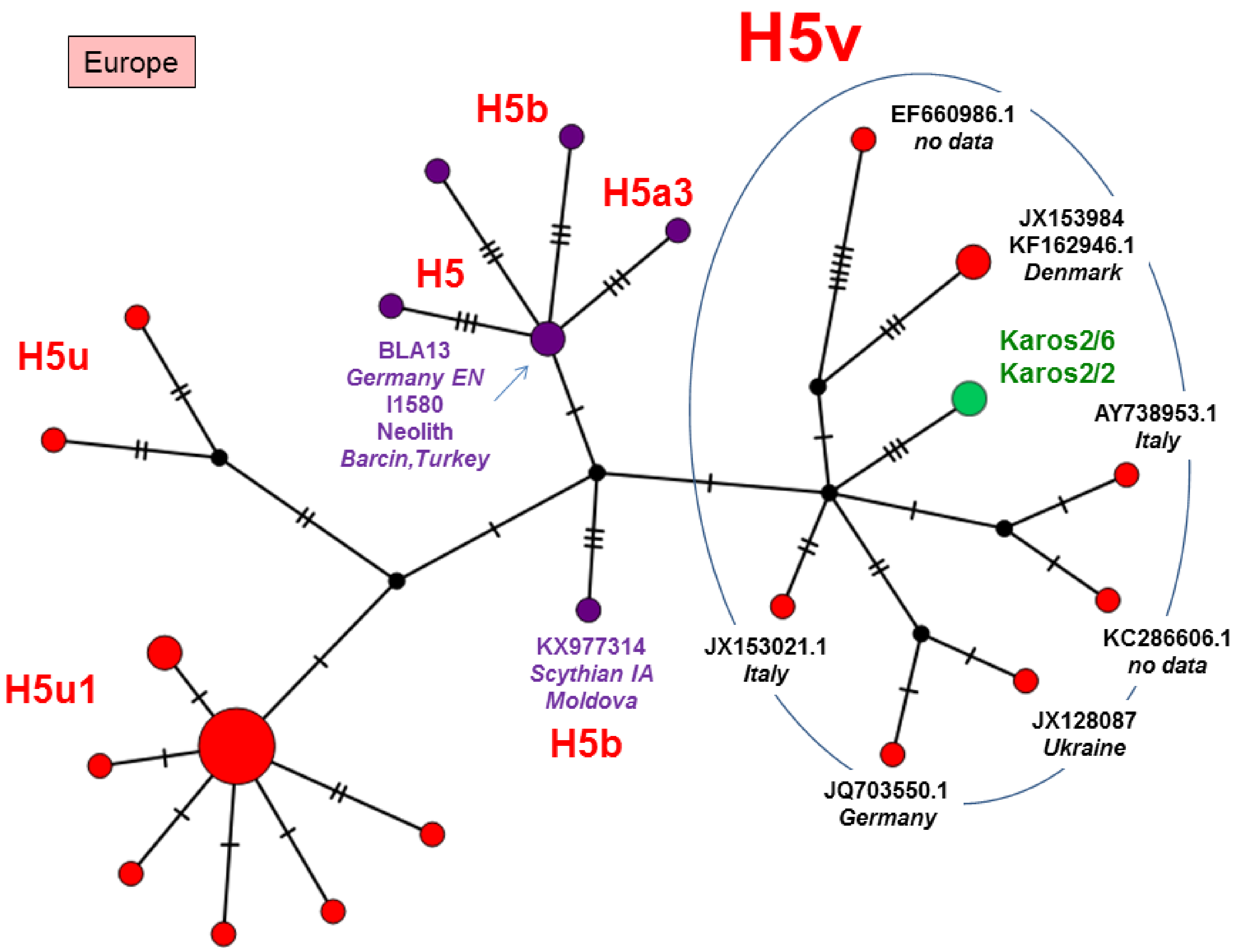
Europe
H5v
H5b
H5a3
H5
EF660986.1
no data
JX153984
KF162946.1
Denmark
Karos2/6
Karos2/2
AY738953.1
Italy
H5u
BLA13
Germany EN
I1580
Neolith
Barcin,Turkey
KX977314
Scythian IA
Moldova
H5b
JX153021.1
Italy
KC286606.1
no data
JX128087
Ukraine
JQ703550.1
Germany
H5u1

**Network 21.**

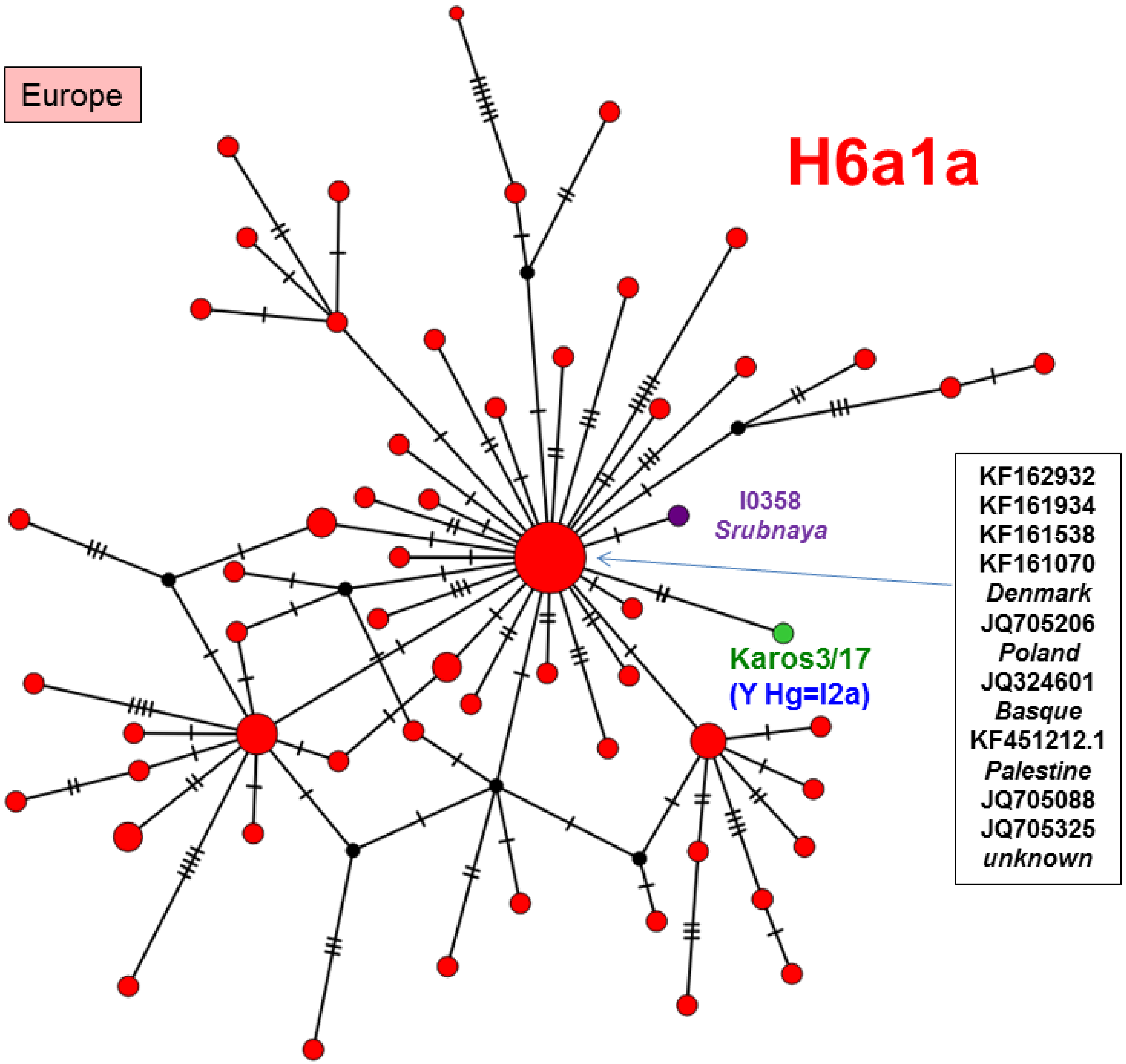
Europe
H6a1a
I0358
Srubnaya
Karos3/17
(Y Hg=I2a)
KF162932
KF161934
KF161538
KF161070
Denmark
JQ705206
Poland
JQ324601
Basque
KF451212.1
Palestine
JQ705088
JQ705325
unknown

**Network 22.**

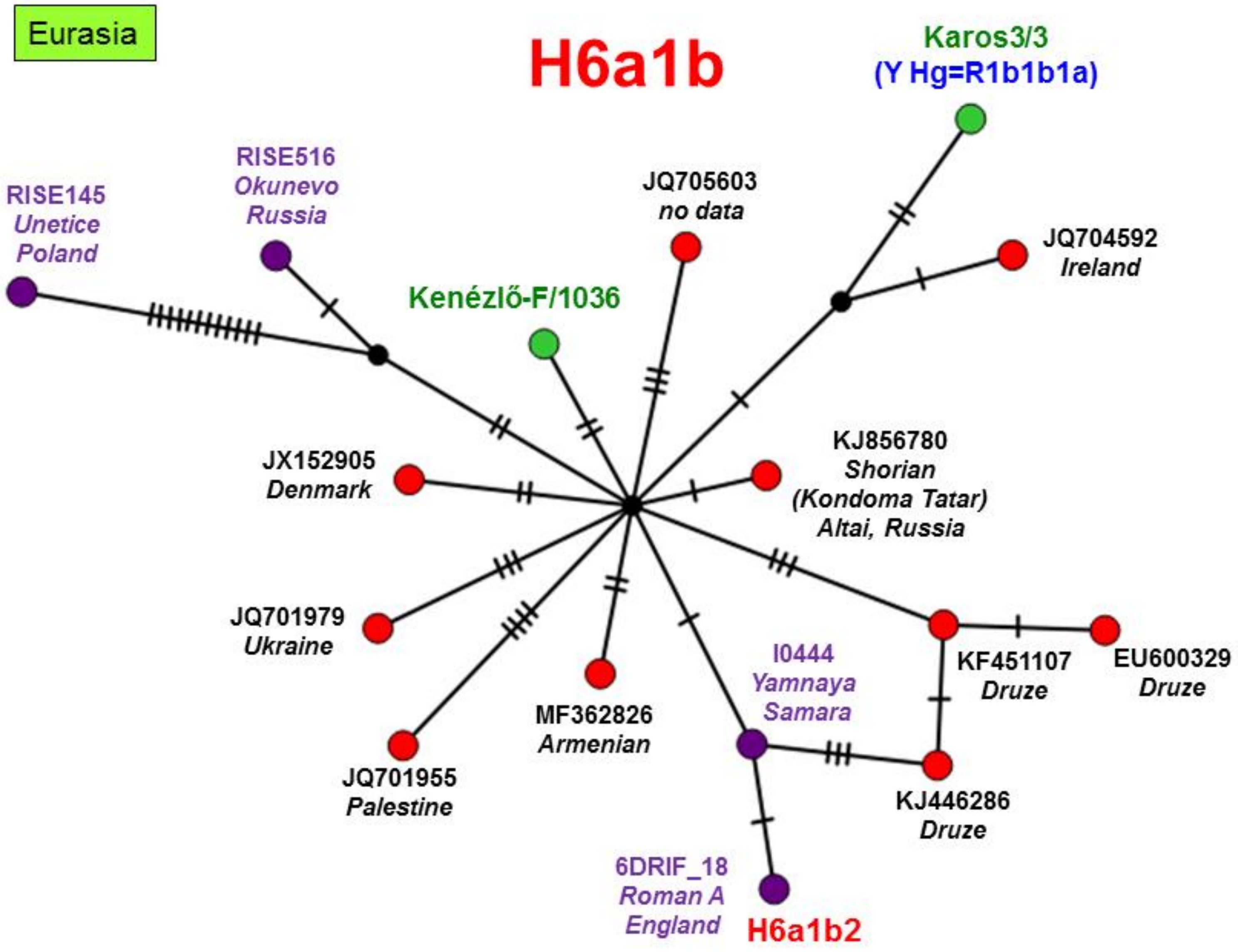

Eurasia
H6a1b
Karos3/3
(Y Hg=R1b1b1a)
RISE516
Okunevo
Russia
RISE145
Unetice
Poland
JQ705603
no data
JQ704592
Ireland
Kenézlő-F/1036
KJ856780
Shorian
(Kondoma Tatar)
Altai, Russia
JX152905
Denmark
JQ701979
Ukraine
MF362826
Armenian
I0444
Yamnaya
Samara
KF451107
Druze
EU600329
Druze
JQ701955
Palestine
KJ446286
Druze
6DRIF_18
Roman A
England
H6a1b2


**Network 23.**

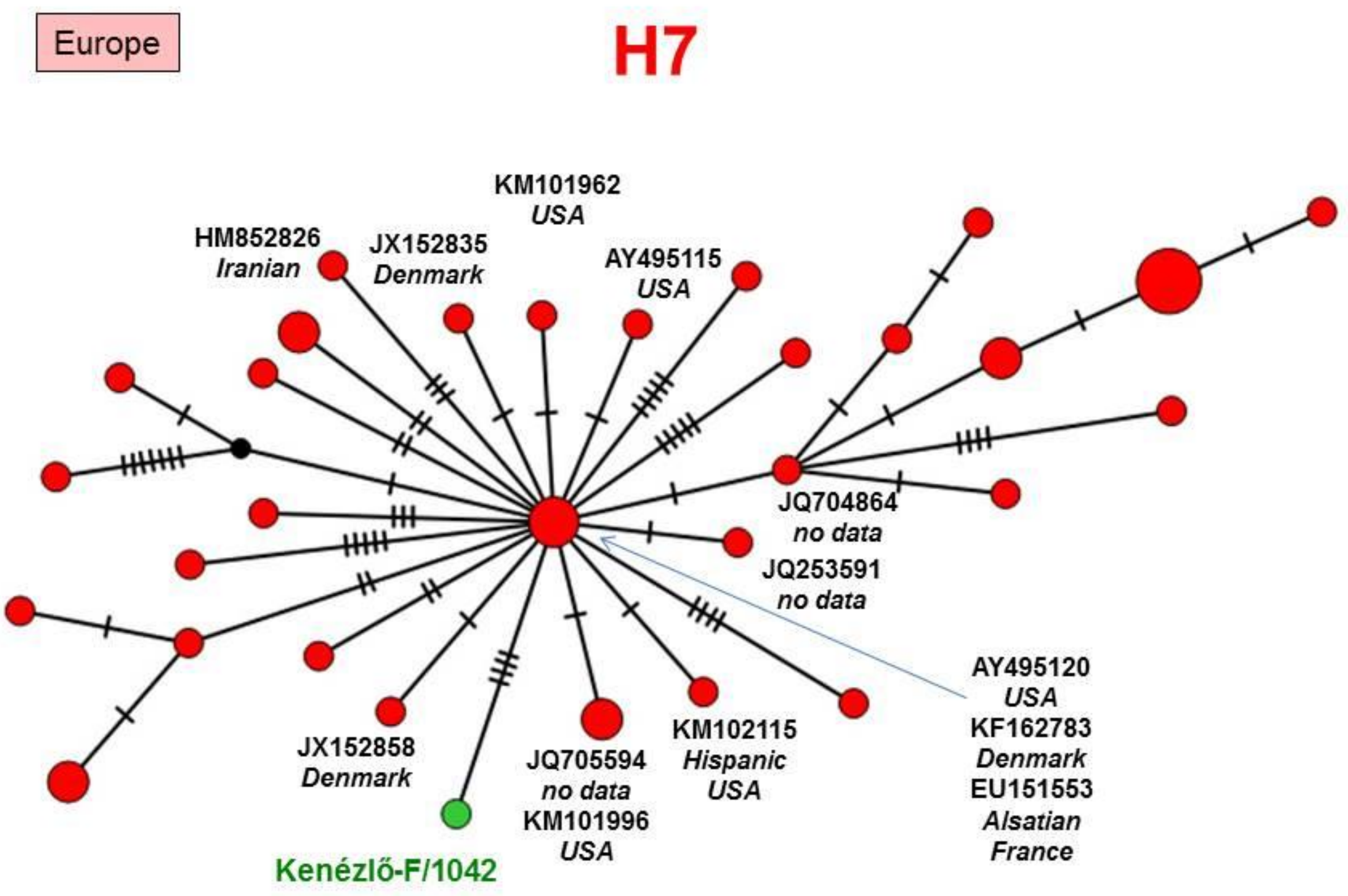
Europe
H7
KM101962
USA
HM852826
Iranian
JX152835
Denmark
AY495115
USA
JQ704864
no data
JQ253591
no data
AY495120
USA
KF162783
Denmark
EU151553
Alsatian
France
KM102115
Hispanic
USA
JQ705594
no data
KM101996
USA
JX152858
Denmark
Kenézlő-F/1042

**Network 24.**

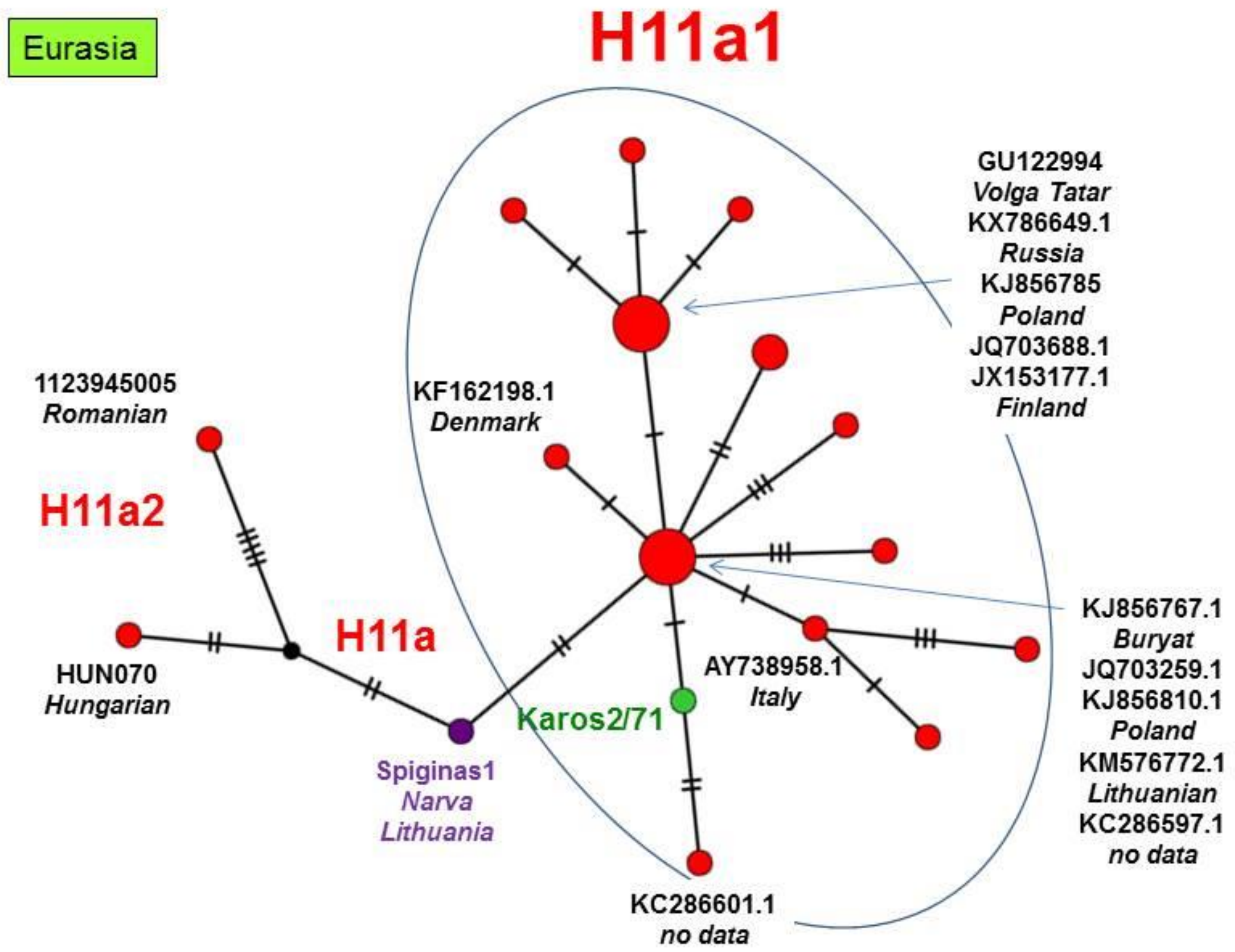
Eurasia
H11a1
GU122994
Volga Tatar
KX786649.1
Russia
KJ856785
Poland
JQ703688.1
JX153177.1
Finland
1123945005
Romanian
KF162198.1
Denmark
H11a2
H11a
HUN070
Hungarian
AY738958.1
Italy
Karos2/71
Spiginas1
Narva
Lithuania
KJ856767.1
Buryat
JQ703259.1
KJ856810.1
Poland
KM576772.1
Lithuanian
KC286597.1
no data
KC286601.1
no data

**Network 25.**

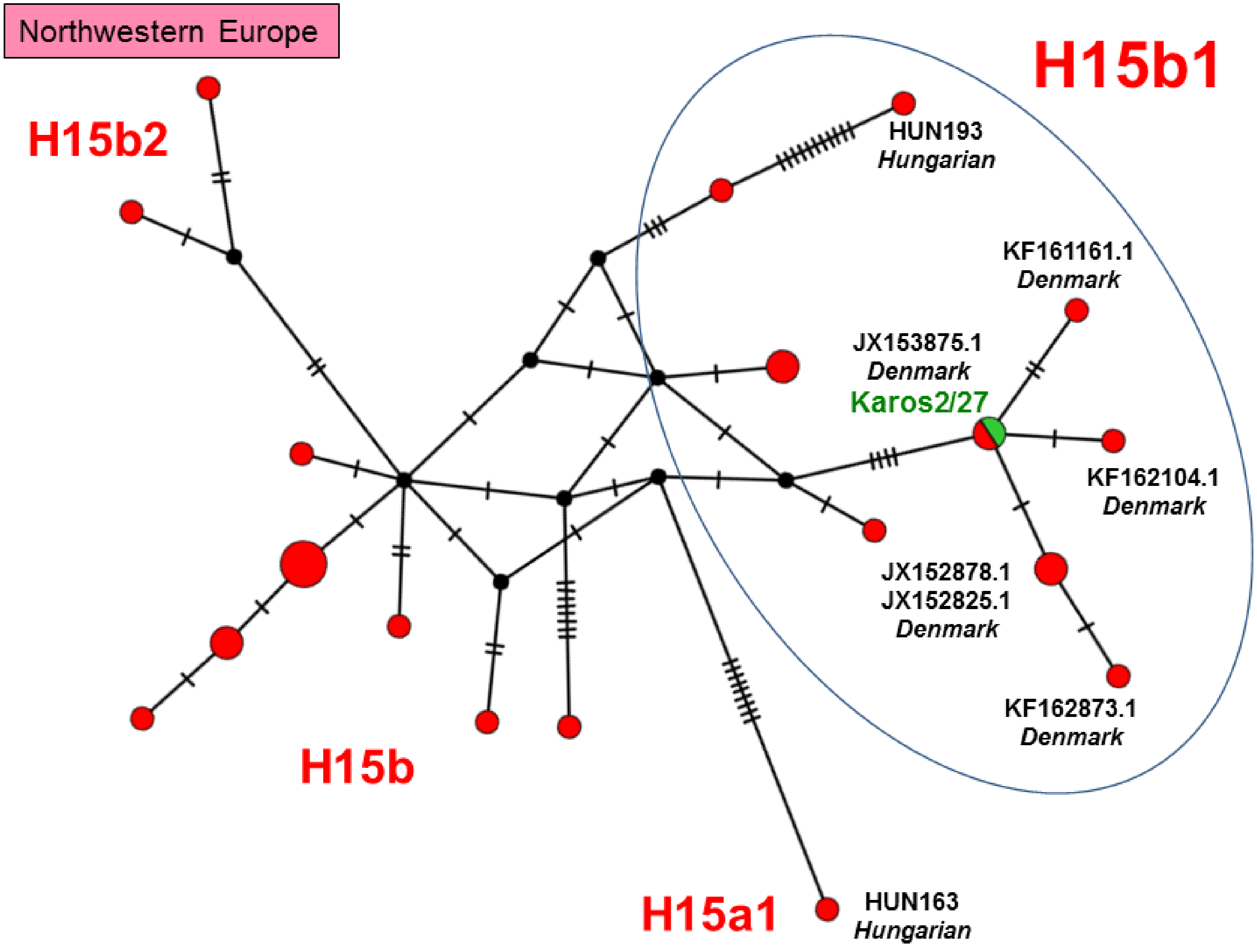
Northwestern Europe
H15b2
H15b1
HUN193
Hungarian
KF161161.1
Denmark
JX153875.1
Denmark
Karos2/27
KF162104.1
Denmark
JX152878.1
JX152825.1
Denmark
KF162873.1
Denmark
H15b
H15a1
HUN163
Hungarian

**Network 26.**

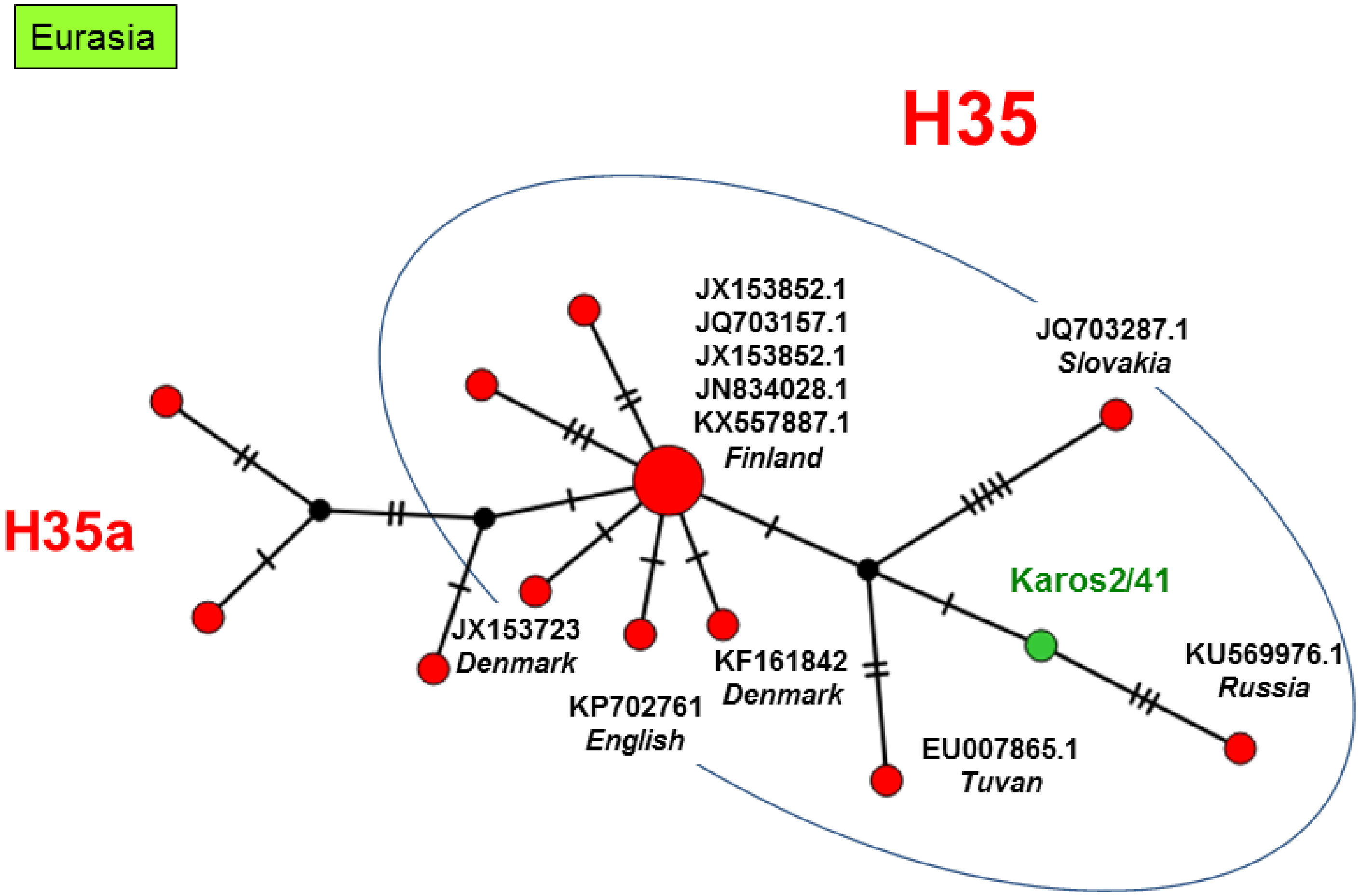
Eurasia
H35
H35a
JX153852.1
JQ703157.1
JX153852.1
JN834028.1
KX557887.1
Finland
JQ703287.1
Slovakia
Karos2/41
KU569976.1
Russia
JX153723
Denmark
KF161842
Denmark
KP702761
English
EU007865.1
Tuvan

**Network 27.**

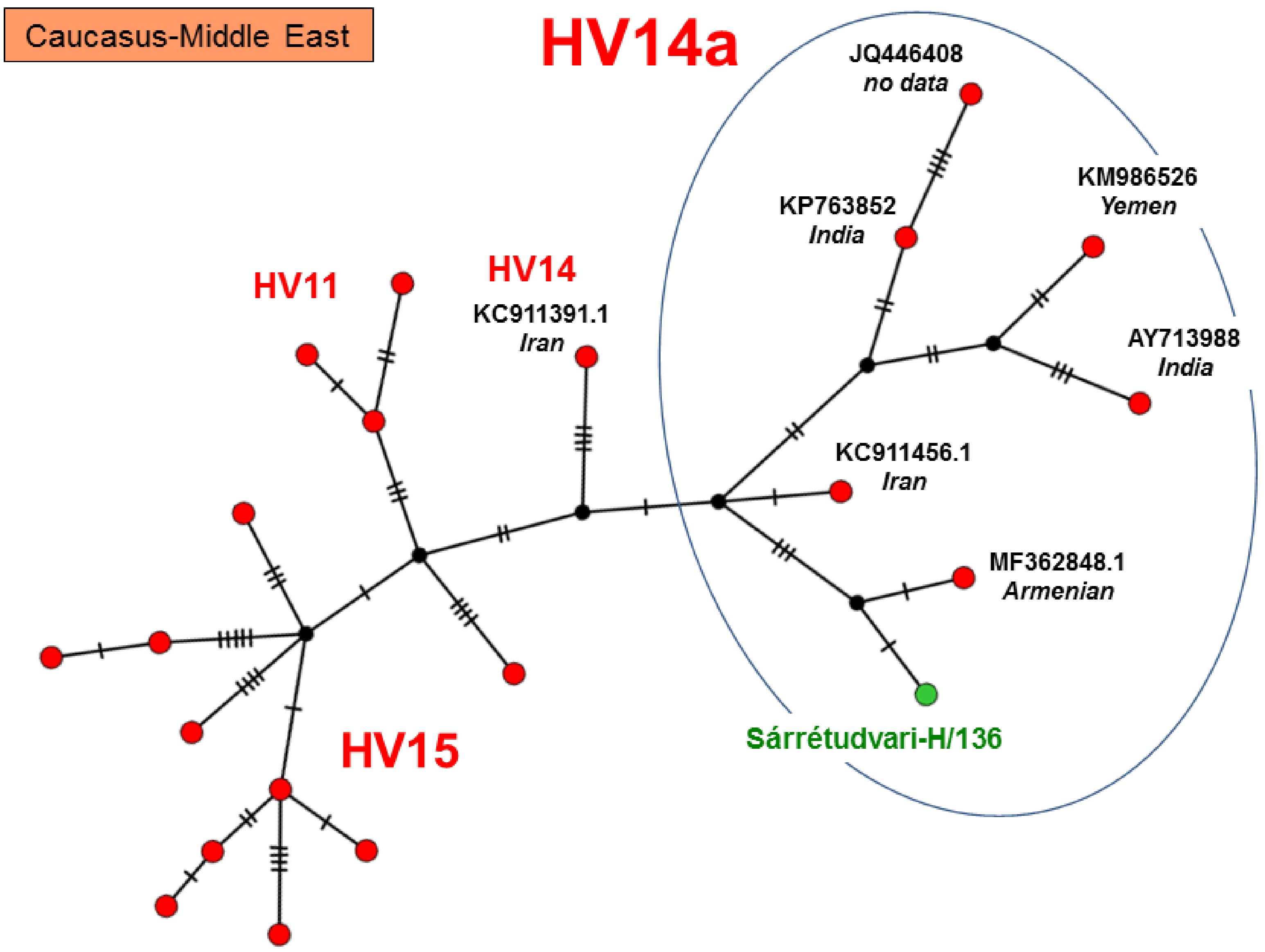
Caucasus-Middle East
HV14a
JQ446408
no data
KP763852
India
KM986526
Yemen
AY713988
India
HV11
HV14
KC911391.1
Iran
KC911456.1
Iran
MF362848.1
Armenian
HV15
Sárrétudvari-H/136

**Network 28.**

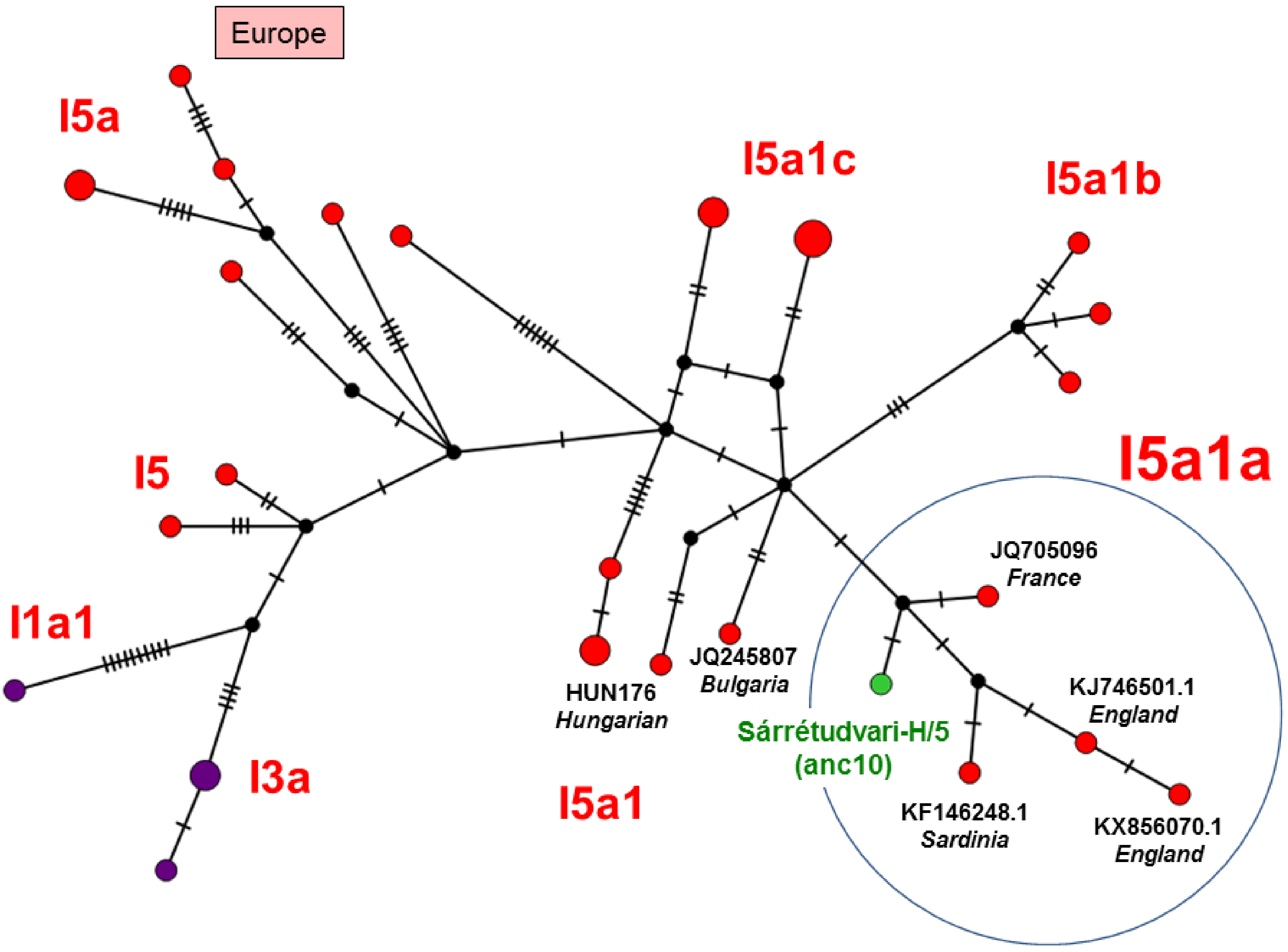
Europe
I5a
I5a1c
I5a1b
I5a1a
I5
I1a1
I3a
I5a1
JQ705096
France
HUN176
Hungarian
JQ245807
Bulgaria
Sárrétudvari-H/5
(anc10)
KJ746501.1
England
KF146248.1
Sardinia
KX856070.1
England

**Network 29.**

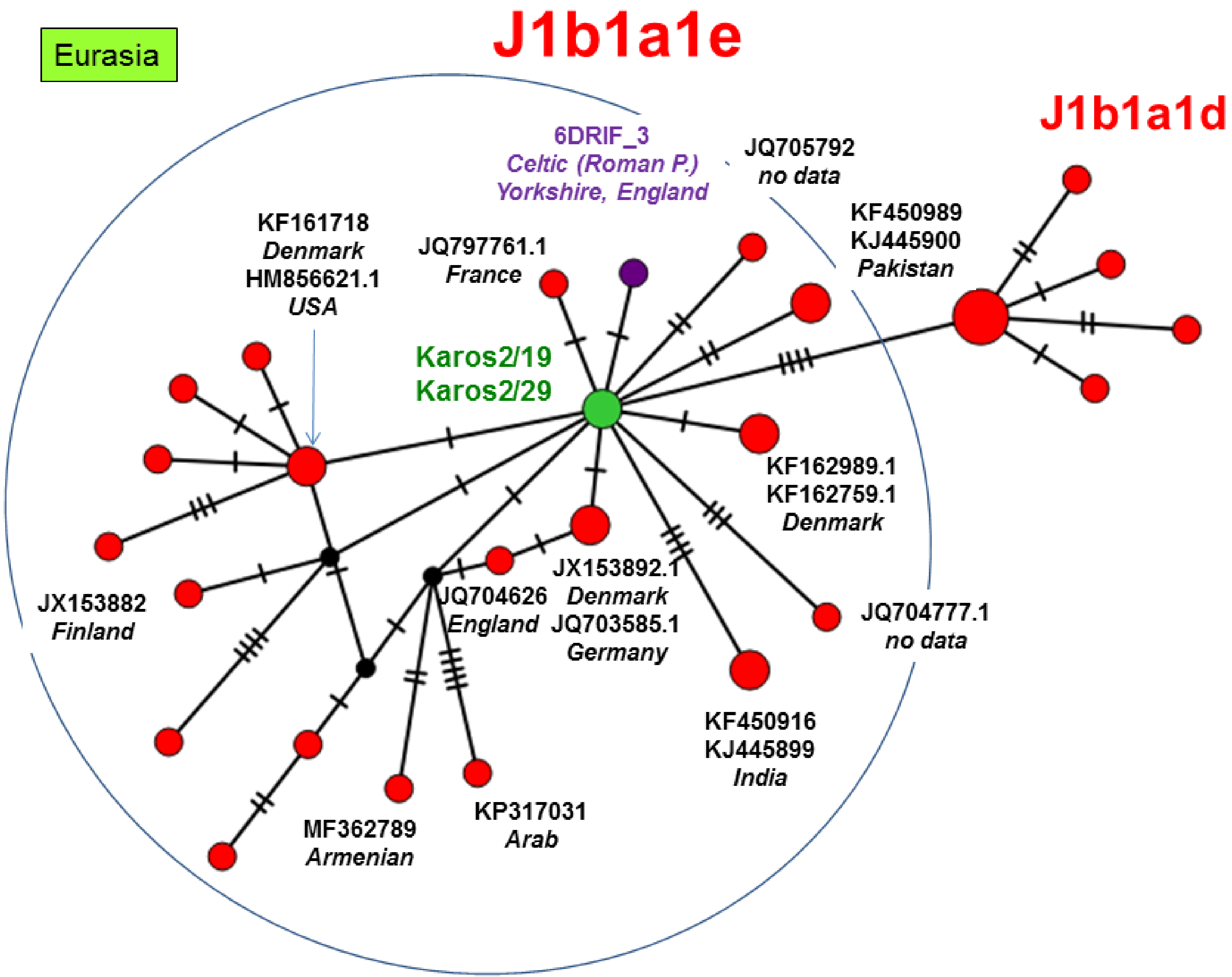
Eurasia
J1b1a1e
J1b1a1d
6DRIF_3
Celtic (Roman P.)
Yorkshire, England
JQ705792
no data
KF450989
KJ445900
Pakistan
KF161718
Denmark
HM856621.1
USA
JQ797761.1
France
Karos2/19
Karos2/29
KF162989.1
KF162759.1
Denmark
JX153892.1
Denmark
JQ703585.1
Germany
JX153882
Finland
JQ704626
England
JQ704777.1
no data
KF450916
KJ445899
India
MF362789
Armenian
KP317031
Arab

**Network 30.**

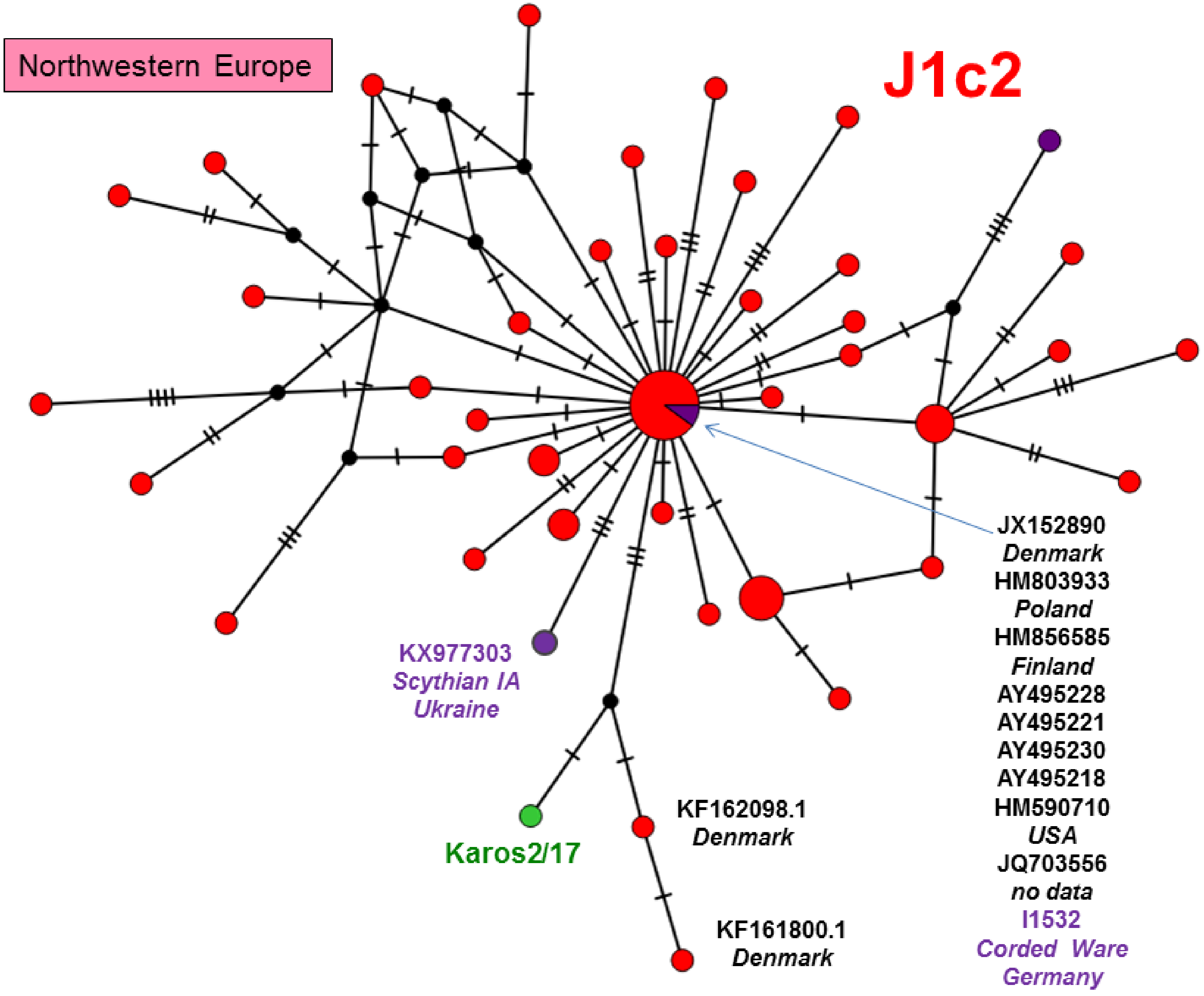

Northwestern Europe
J1c2
JX152890
Denmark
HM803933
Poland
HM856585
Finland
AY495228
AY495221
AY495230
AY495218
HM590710
USA
JQ703556
no data
I1532
Corded Ware
Germany
KX977303
Scythian IA
Ukraine
Karos2/17
KF162098.1
Denmark
KF161800.1
Denmark


**Network 31.**

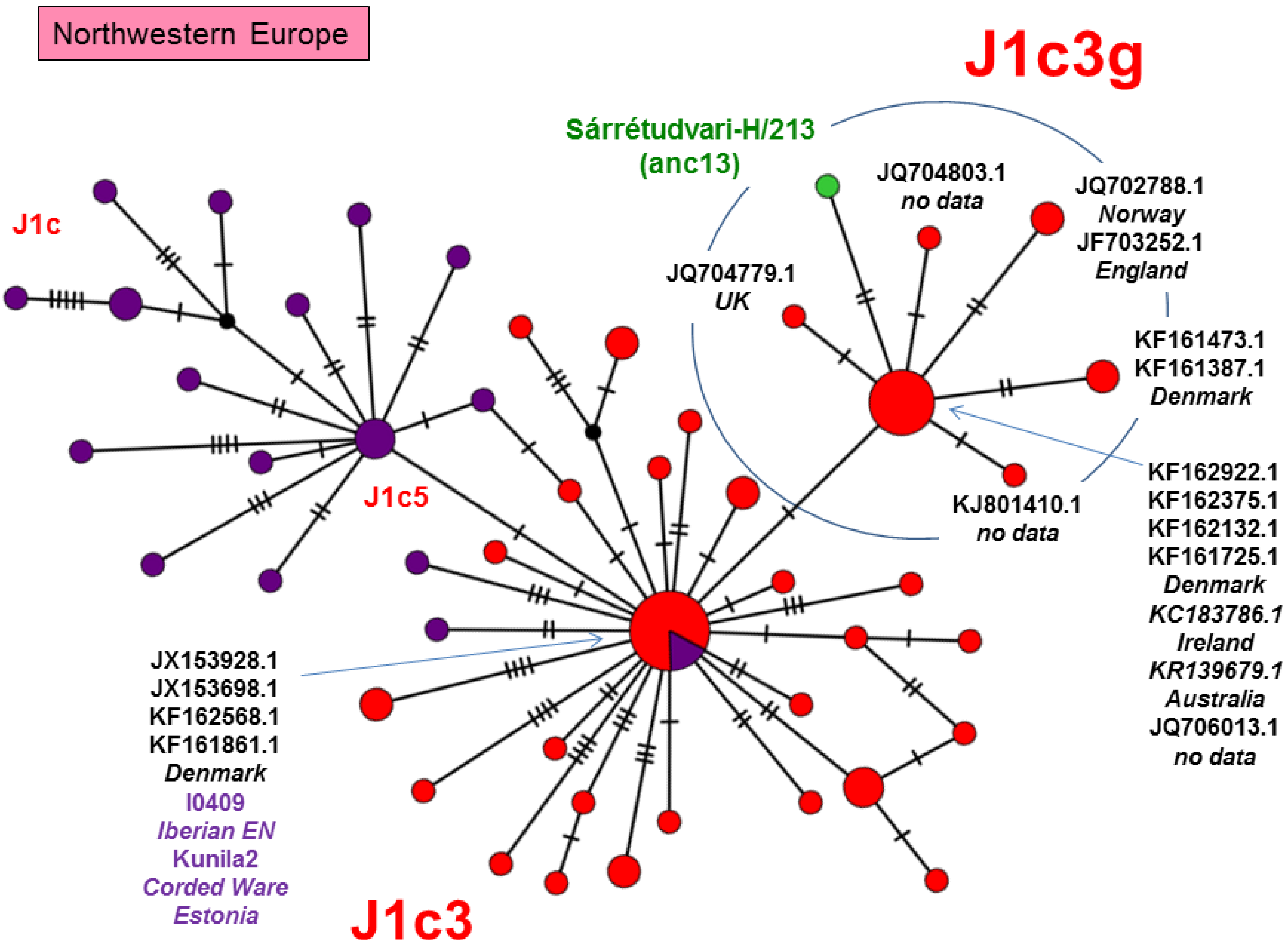

Northwestern Europe
J1c3g
Sárrétudvari-H/213
(anc13)
JQ704803.1
no data
JQ702788.1
Norway
JF703252.1
England
J1c
JQ704779.1
UK
KF161473.1
KF161387.1
Denmark
J1c5
KF162922.1
KF162375.1
KF162132.1
KF161725.1
Denmark
KC183786.1
Ireland
KR139679.1
Australia
JQ706013.1
no data
KJ801410.1
no data
JX153928.1
JX153698.1
KF162568.1
KF161861.1
Denmark
I0409
Iberian EN
Kunila2
Corded Ware
Estonia
J1c3


**Network 32.**

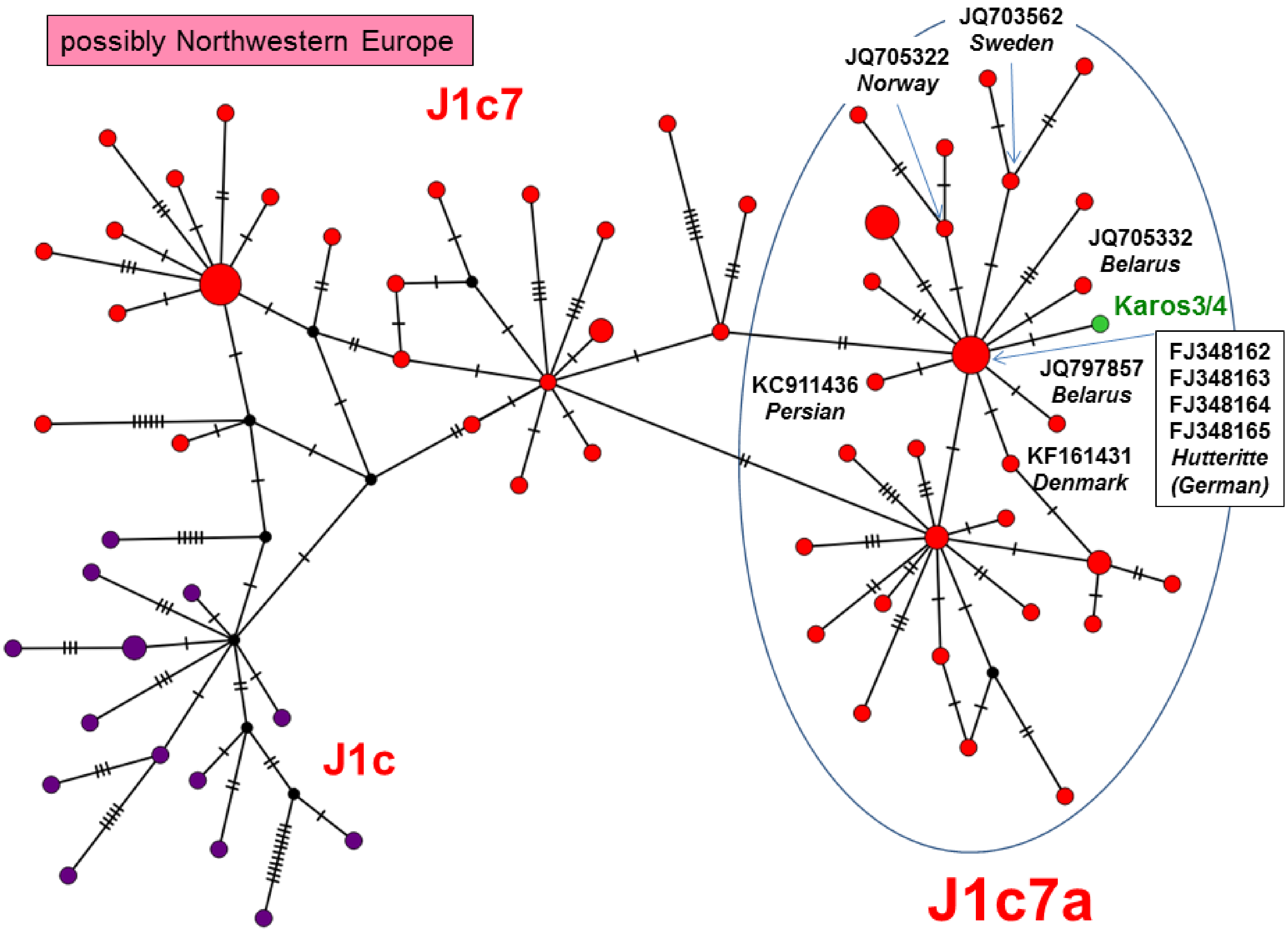
possibly Northwestern Europe
J1c7
J1c
J1c7a
JQ703562
Sweden
JQ705322
Norway
JQ705332
Belarus
Karos3/4
FJ348162
FJ348163
FJ348164
FJ348165
Hutteritte
(German)
JQ797857
Belarus
KC911436
Persian
KF161431
Denmark

**Network 33.**

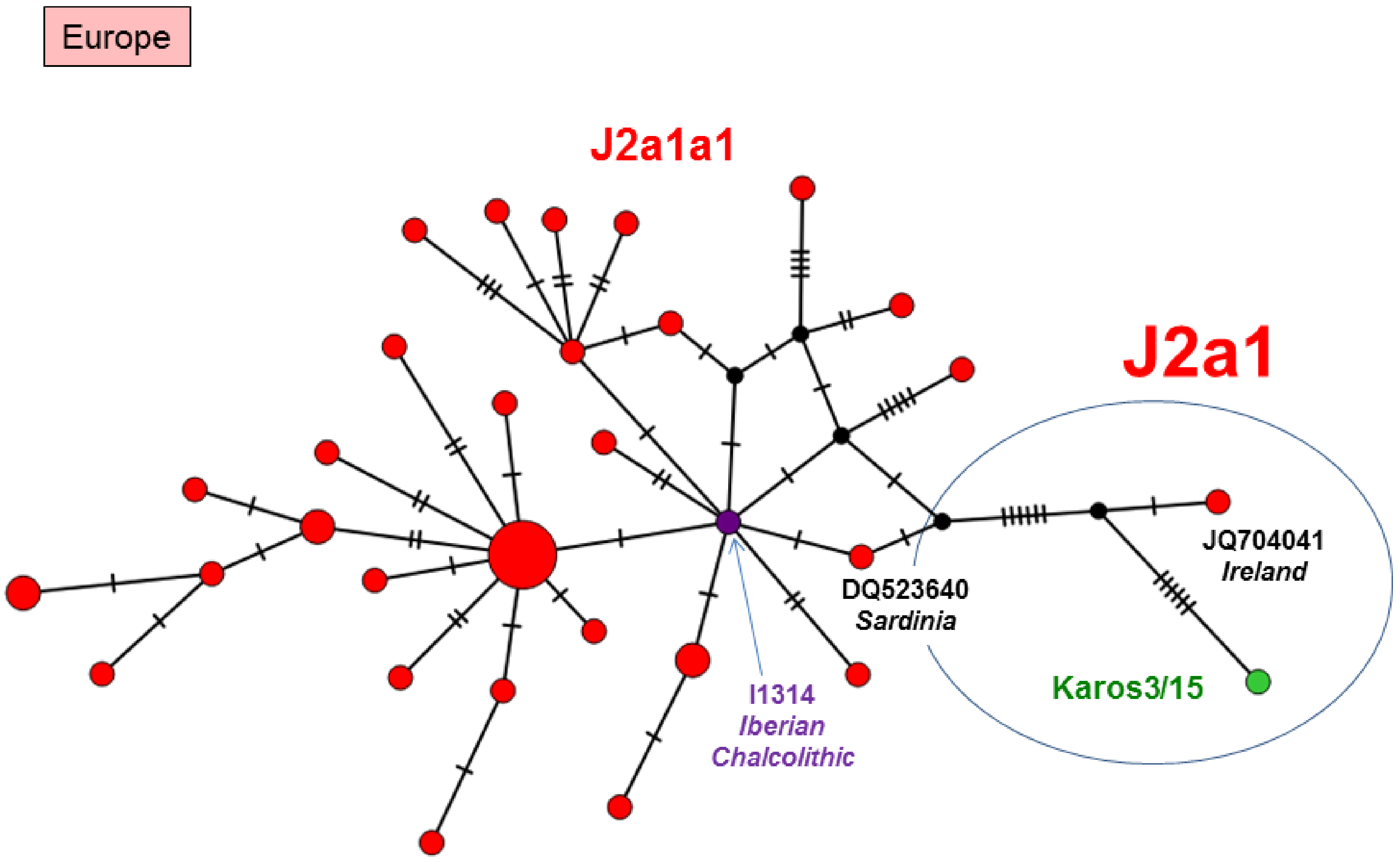
Europe
J2a1a1
J2a1
JQ704041
Ireland
DQ523640
Sardinia
Karos3/15
I1314
Iberian
Chalcolithic

**Network 34.**

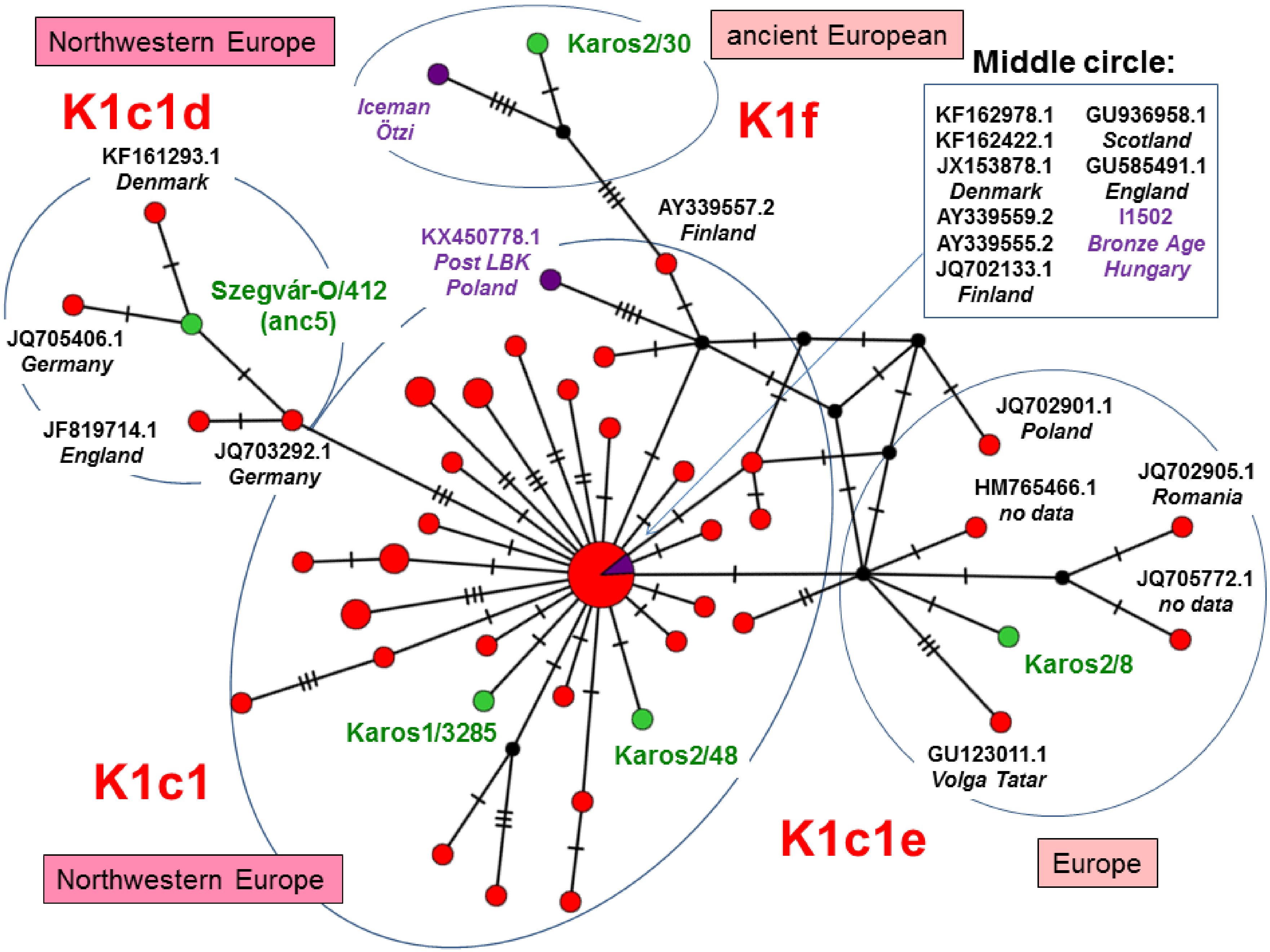
Northwestern Europe
K1c1d
KF161293.1
Denmark
JQ705406.1
Germany
Szegvár-O/412
(anc5)
JF819714.1
England
JQ703292.1
Germany
Karos2/30
Iceman
Ötzi
K1f
ancient European
Middle circle:
KF162978.1
KF162422.1
JX153878.1
Denmark
AY339559.2
AY339555.2
JQ702133.1
Finland
GU936958.1
Scotland
GU585491.1
England
I1502
Bronze Age
Hungary
AY339557.2
Finland
KX450778.1
Post LBK
Poland
JQ702901.1
Poland
HM765466.1
no data
JQ702905.1
Romania
JQ705772.1
no data
Karos2/8
GU123011.1
Volga Tatar
Karos1/3285
Karos2/48
K1c1
K1c1e
Northwestern Europe
Europe

**Network 35.**

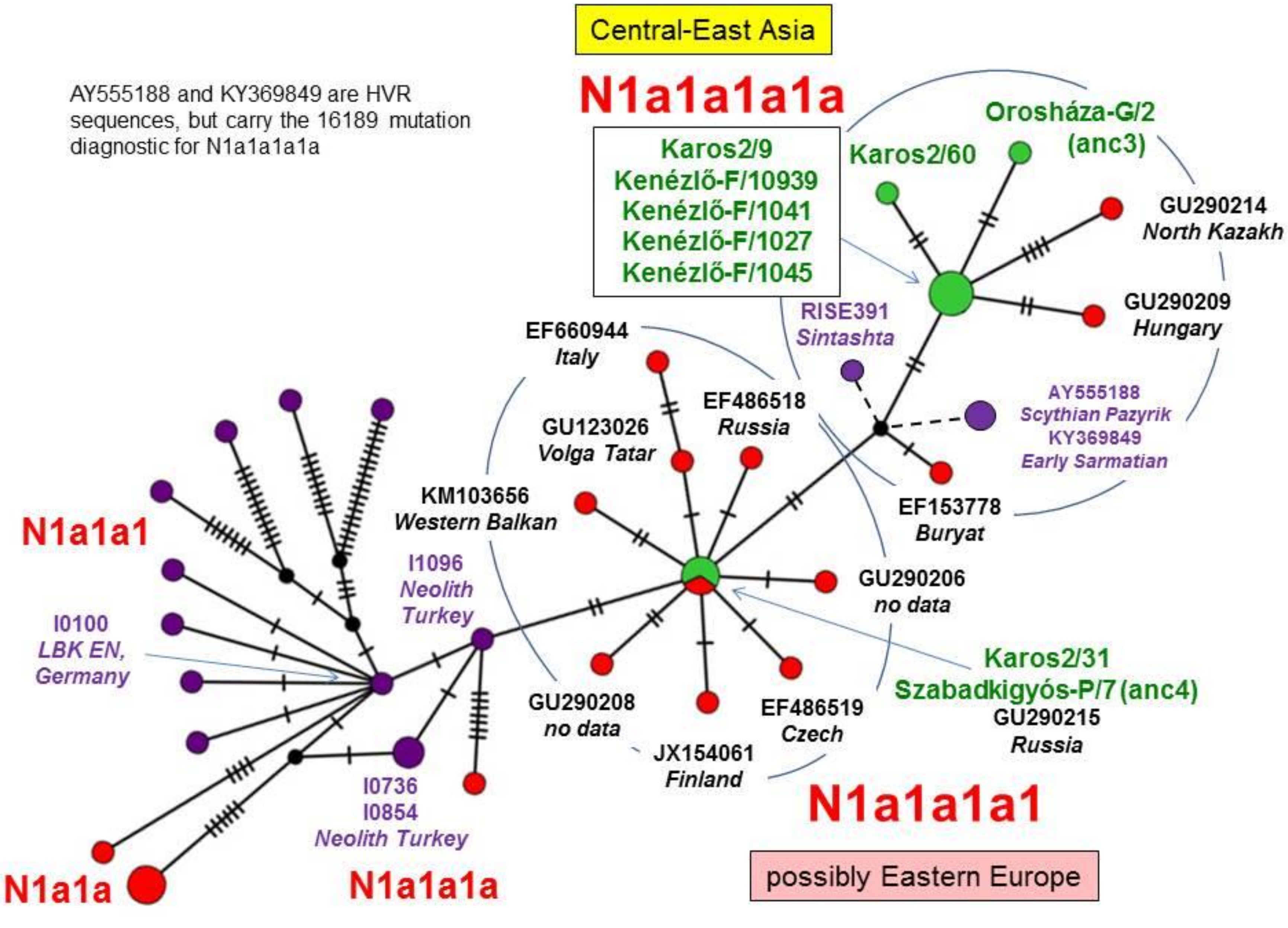
Central-East Asia
N1a1a1a1a
AY555188 and KY369849 are HVR sequences, but carry the 16189 mutation diagnostic for N1a1a1a1a
Karos2/9
Kenézlő-F/10939
Kenézlő-F/1041
Kenézlő-F/1027
Kenézlő-F/1045
Karos2/60
Orosháza-G/2 (anc3)
GU290214 North Kazakh
GU290209 Hungary
RISE391 Sintashta
AY555188 Scythian Pazyrik
KY369849 Early Sarmatian
EF153778 Buryat
EF660944 Italy
EF486518 Russia
GU123026 Volga Tatar
KM103656 Western Balkan
GU290206 no data
Karos2/31
Szabadkigyós-P/7 (anc4)
GU290215 Russia
GU290208 no data
EF486519 Czech
JX154061 Finland
N1a1a1a1
possibly Eastern Europe
N1a1a1
I1096 Neolith Turkey
I0100 LBK EN, Germany
I0736
I0854
Neolith Turkey
N1a1a
N1a1a1a

**Network 36.**

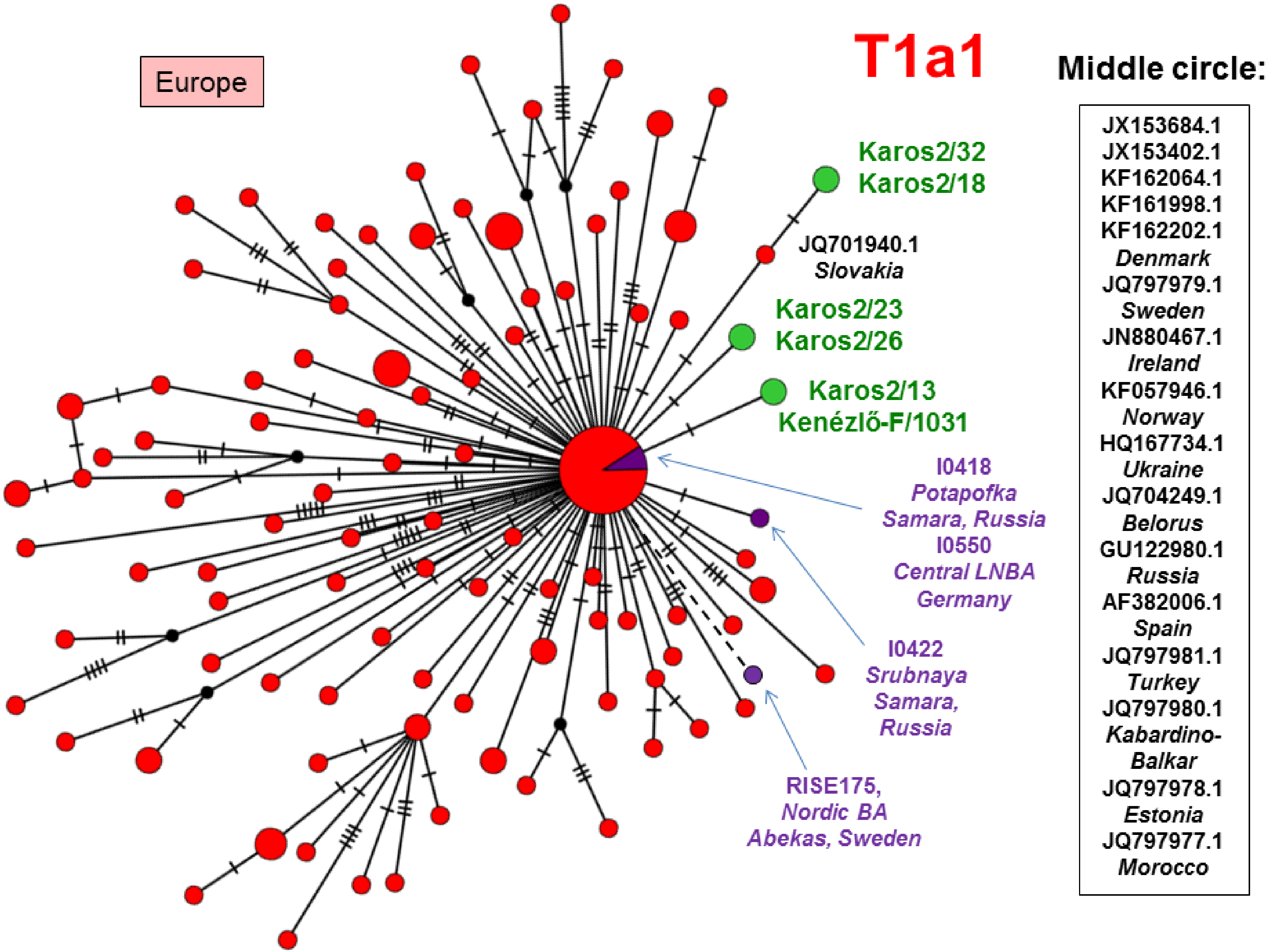
Europe
T1a1
Middle circle:
JX153684.1
JX153402.1
KF162064.1
KF161998.1
KF162202.1
Denmark
JQ797979.1
Sweden
JN880467.1
Ireland
KF057946.1
Norway
HQ167734.1
Ukraine
JQ704249.1
Belorus
GU122980.1
Russia
AF382006.1
Spain
JQ797981.1
Turkey
JQ797980.1
Kabardino-Balkar
JQ797978.1
Estonia
JQ797977.1
Morocco
Karos2/32
Karos2/18
JQ701940.1
Slovakia
Karos2/23
Karos2/26
Karos2/13
Kenézlő-F/1031
I0418
Potapofka
Samara, Russia
I0550
Central LNBA
Germany
I0422
Srubnaya
Samara,
Russia
RISE175,
Nordic BA
Abekas, Sweden

**Network 37.**

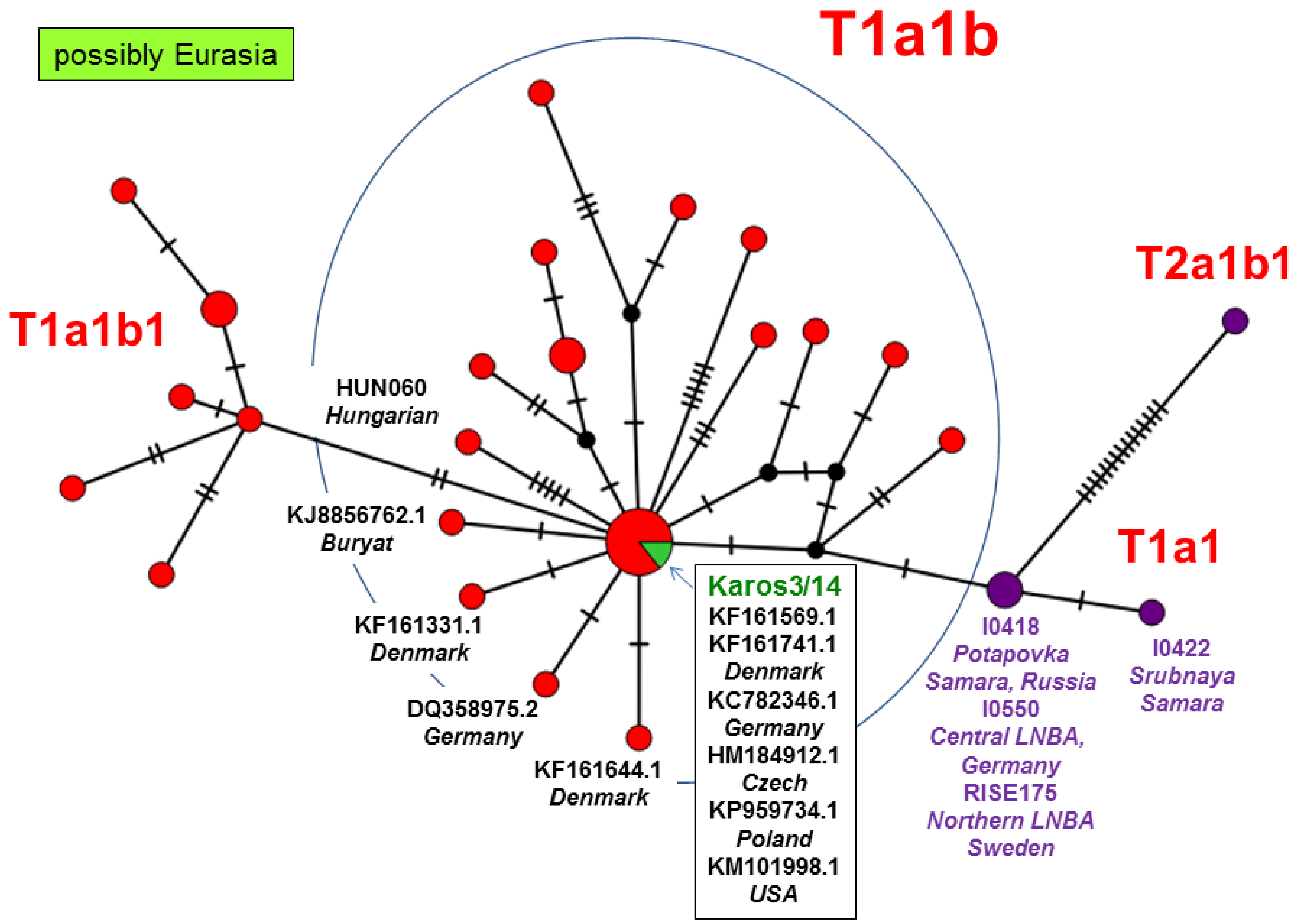
possibly Eurasia
T1a1b
T1a1b1
T2a1b1
T1a1
HUN060
Hungarian
KJ8856762.1
Buryat
KF161331.1
Denmark
DQ358975.2
Germany
KF161644.1
Denmark
Karos3/14
KF161569.1
KF161741.1
Denmark
KC782346.1
Germany
HM184912.1
Czech
KP959734.1
Poland
KM101998.1
USA
I0418
Potapovka
Samara, Russia
I0550
Central LNBA,
Germany
RISE175
Northern LNBA
Sweden
I0422
Srubnaya
Samara

**Network 38.**

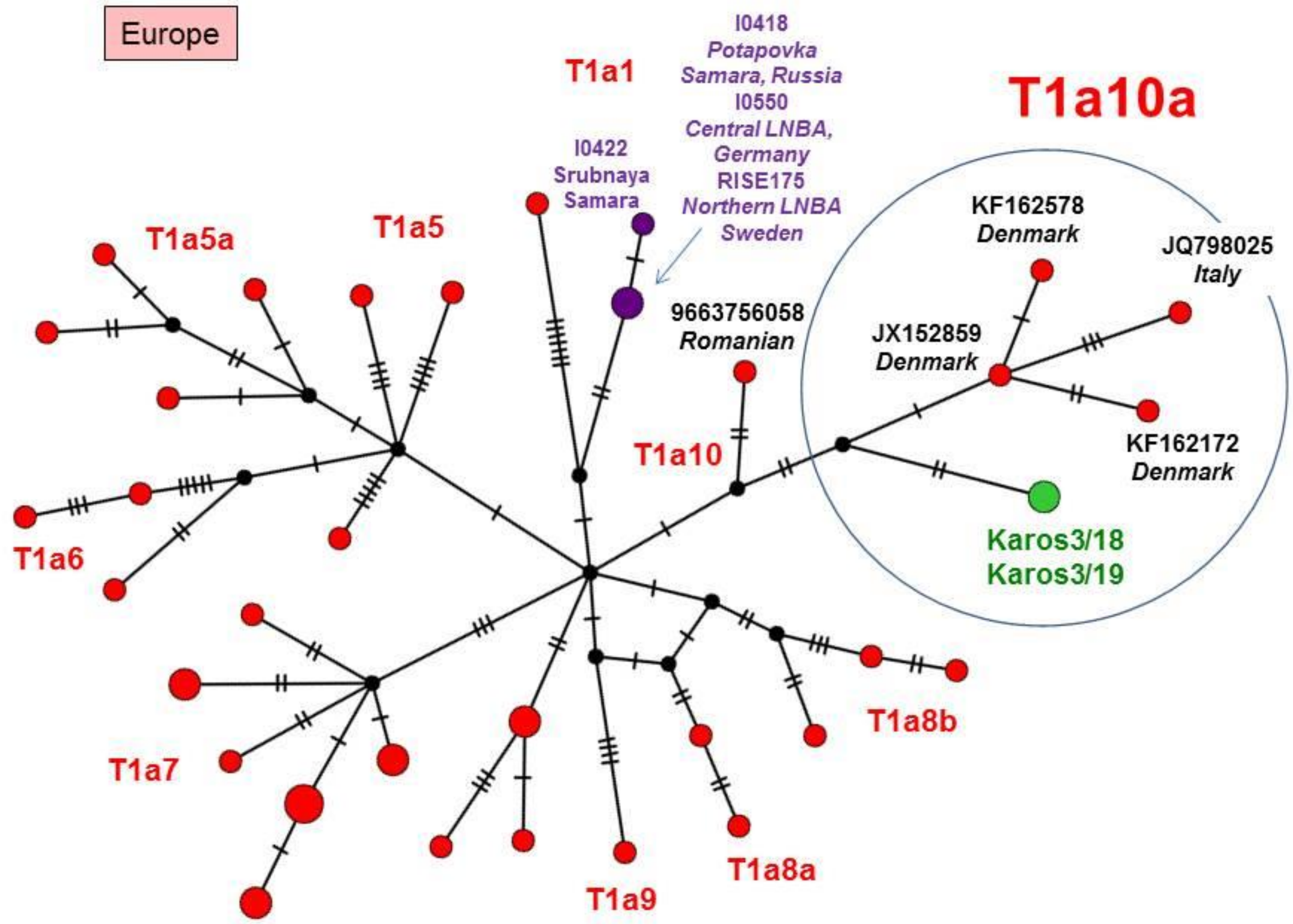
Europe
T1a1
I0418
Potapovka
Samara, Russia
I0550
Central LNBA,
Germany
RISE175
Northern LNBA
Sweden
I0422
Srubnaya
Samara
T1a10a
KF162578
Denmark
JQ798025
Italy
JX152859
Denmark
KF162172
Denmark
9663756058
Romanian
T1a5a
T1a5
T1a10
T1a6
Karos3/18
Karos3/19
T1a7
T1a8b
T1a8a
T1a9

**Network 39.**

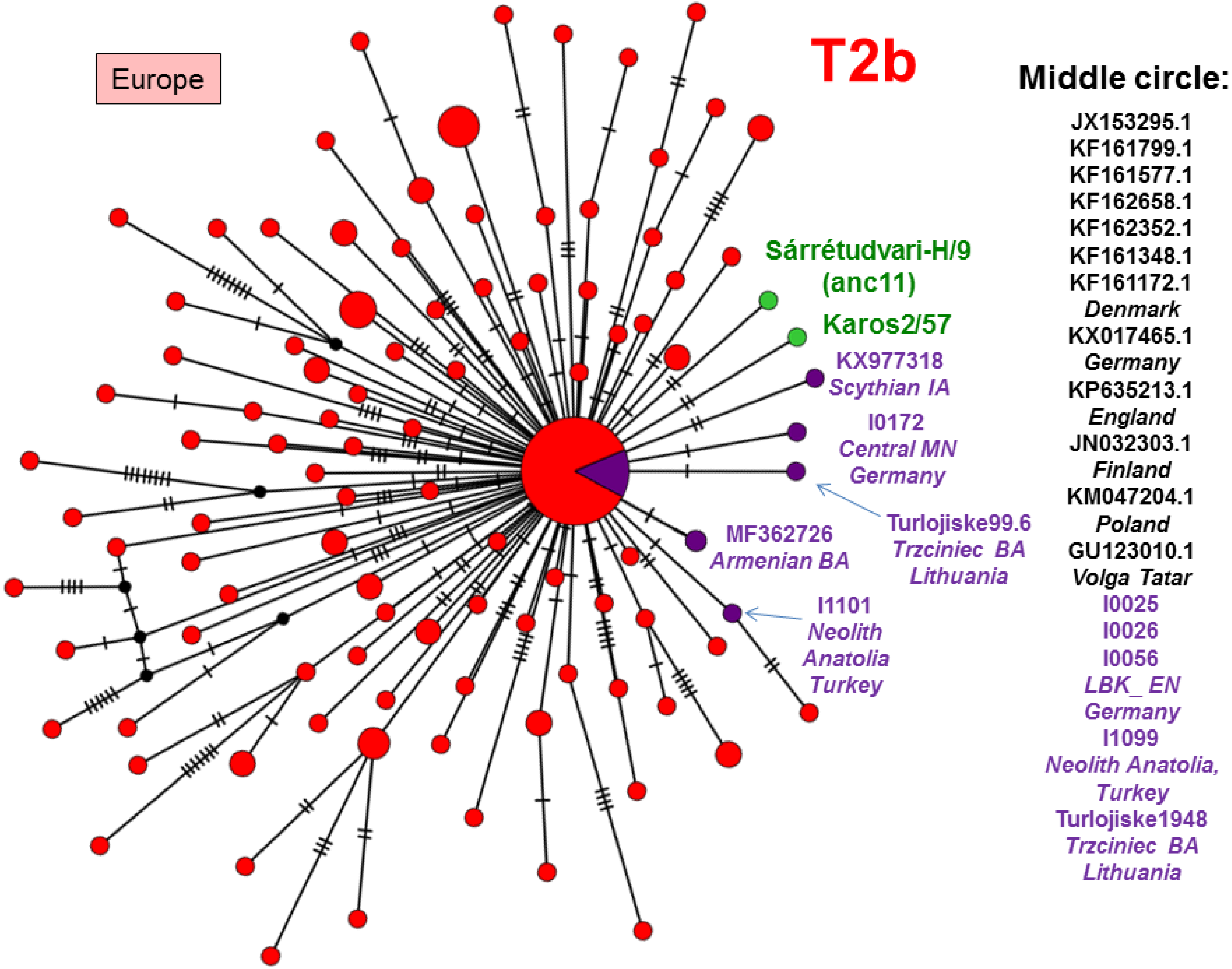
Europe
T2b
Middle circle:
JX153295.1
KF161799.1
KF161577.1
KF162658.1
KF162352.1
KF161348.1
KF161172.1
Denmark
KX017465.1
Germany
KP635213.1
England
JN032303.1
Finland
KM047204.1
Poland
GU123010.1
Volga Tatar
I0025
I0026
I0056
LBK_ EN
Germany
I1099
Neolith Anatolia,
Turkey
Turlojiske1948
Trzciniec BA
Lithuania
Sárrétudvari-H/9
(anc11)
Karos2/57
KX977318
Scythian IA
I0172
Central MN
Germany
Turlojiske99.6
Trzciniec BA
Lithuania
MF362726
Armenian BA
I1101
Neolith
Anatolia
Turkey

**Network 40.**

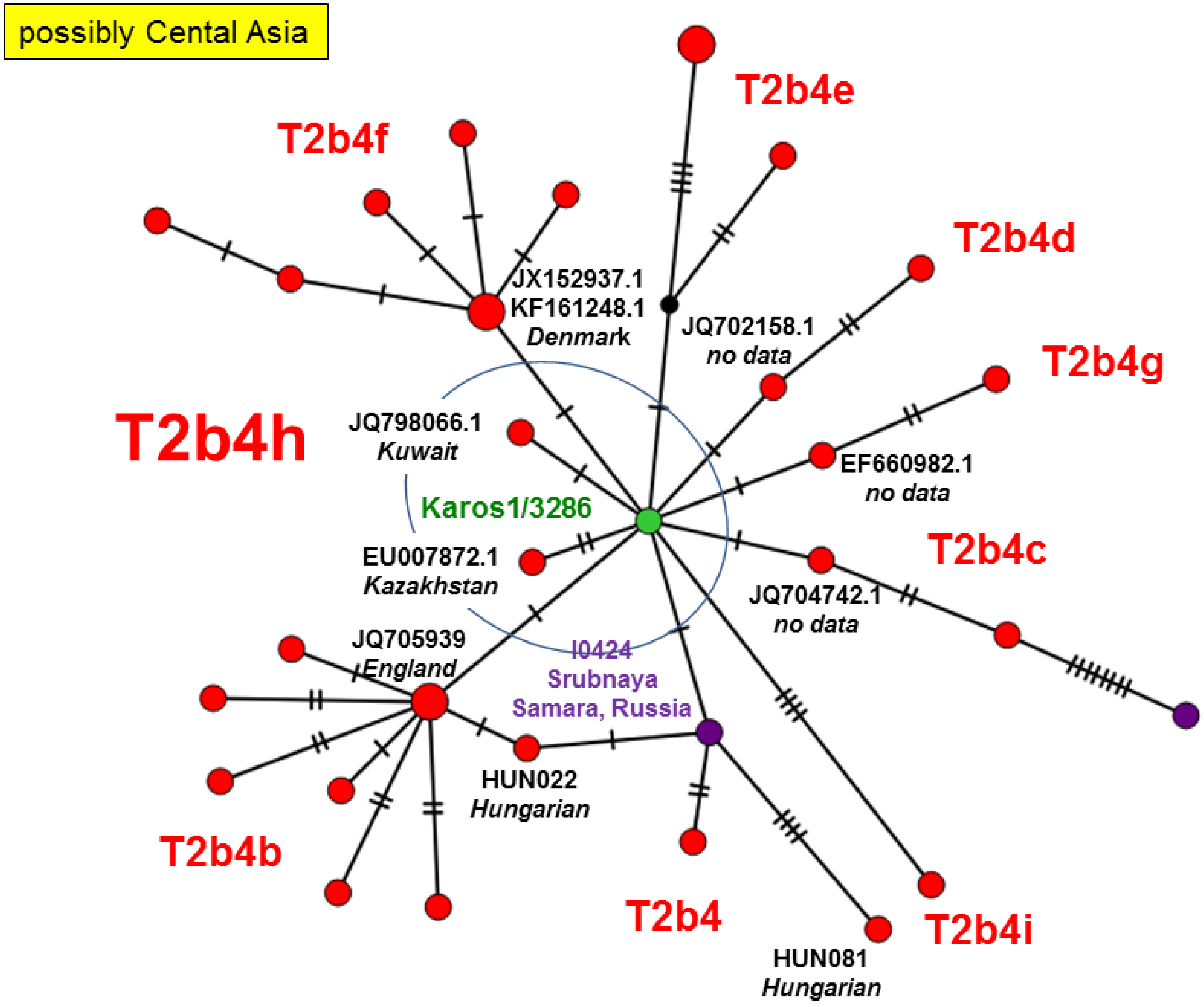
possibly Cental Asia
T2b4e
T2b4f
T2b4d
JX152937.1
KF161248.1
Denmark
JQ702158.1
no data
T2b4g
T2b4h
JQ798066.1
Kuwait
EF660982.1
no data
Karos1/3286
EU007872.1
Kazakhstan
T2b4c
JQ704742.1
no data
JQ705939
England
I0424
Srubnaya
Samara, Russia
HUN022
Hungarian
T2b4b
T2b4
T2b4i
HUN081
Hungarian

**Network 41.**

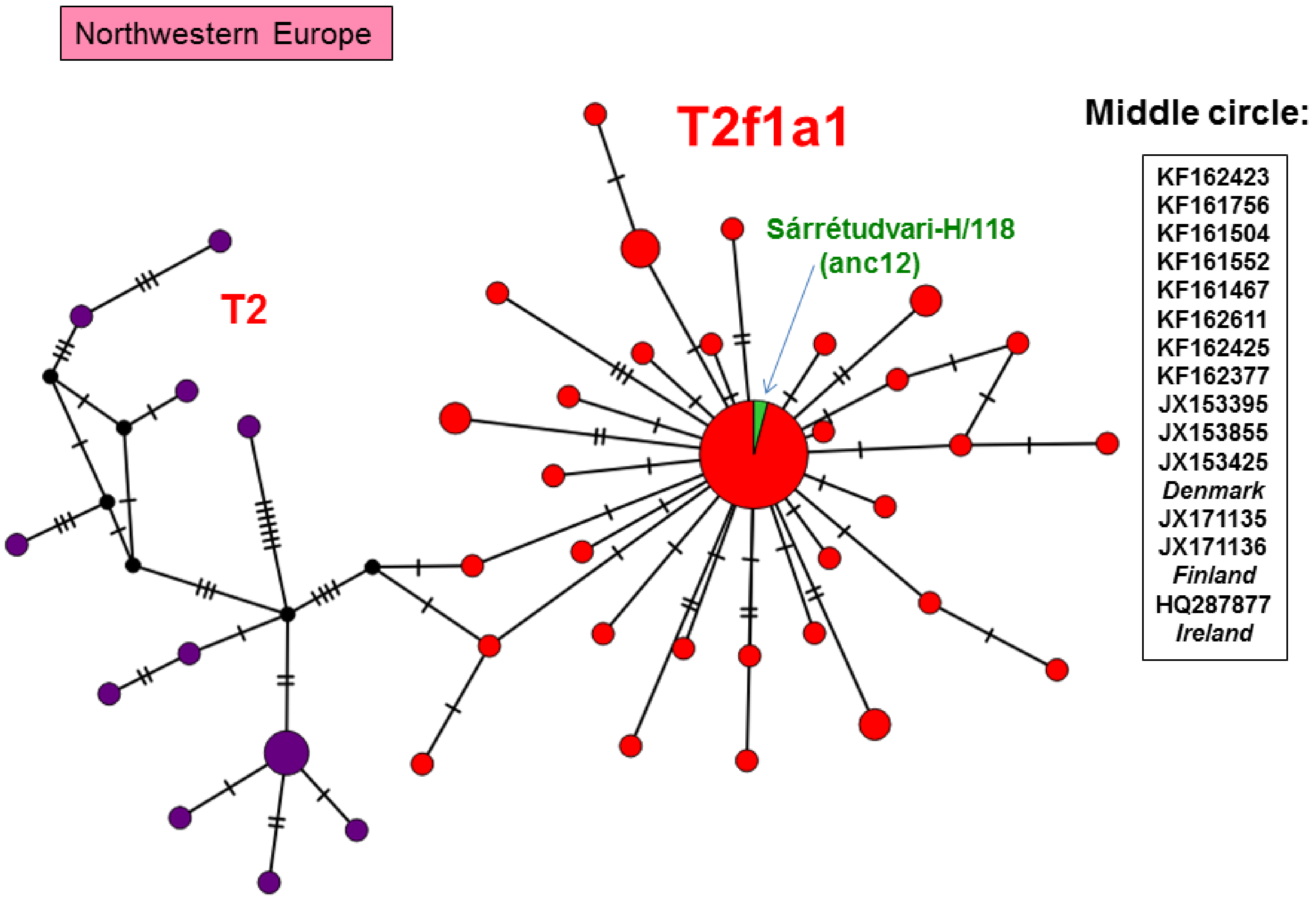

Northwestern Europe
T2f1a1
Middle circle:
KF162423
KF161756
KF161504
KF161552
KF161467
KF162611
KF162425
KF162377
JX153395
JX153855
JX153425
Denmark
JX171135
JX171136
Finland
HQ287877
Ireland
Sárrétudvari-H/118
(anc12)
T2


**Network 42.**

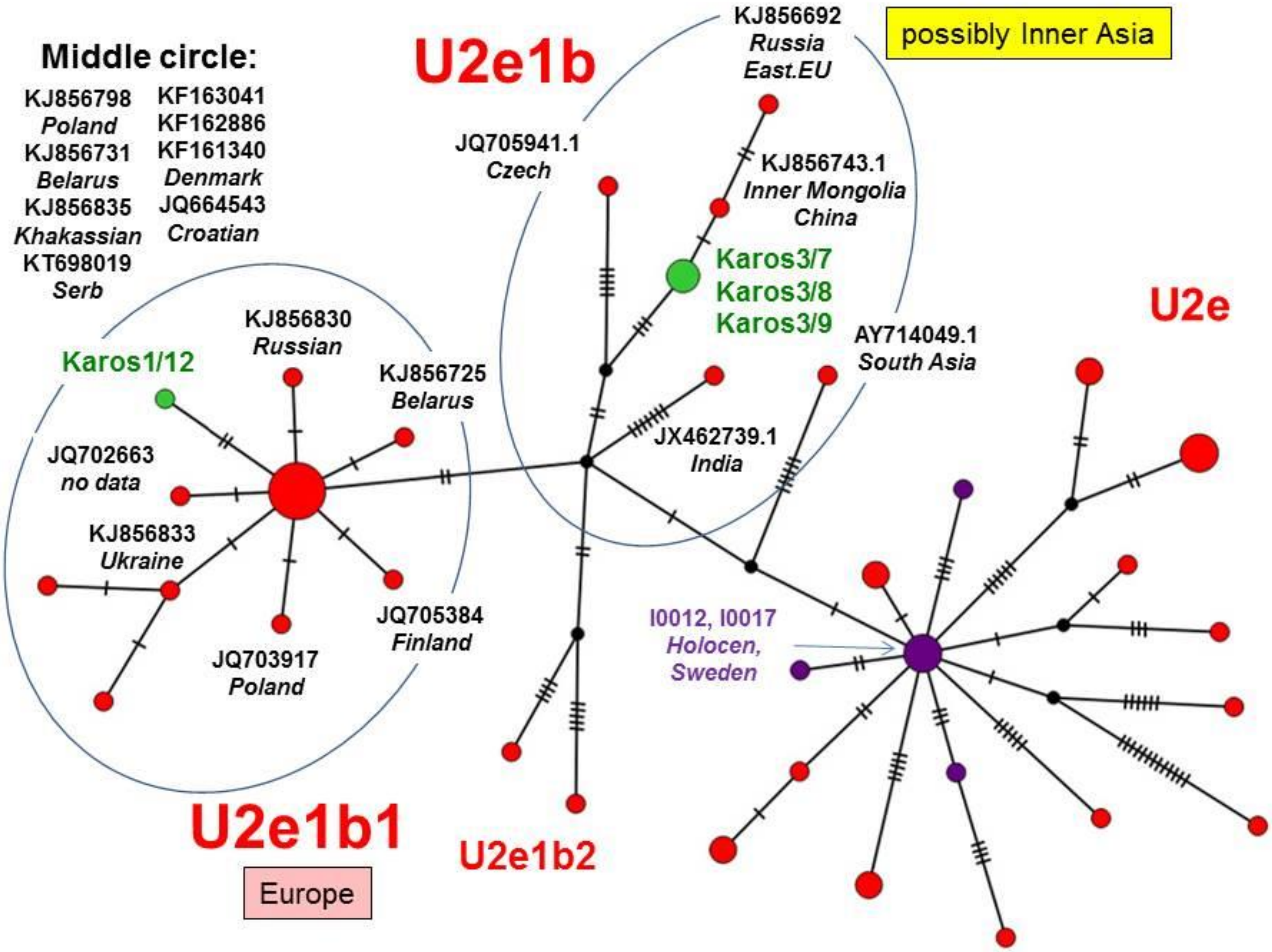
Middle circle:
KJ856798 Poland
KJ856731 Belarus
KJ856835 Khakassian
KT698019 Serb
KF163041
KF162886
KF161340 Denmark
JQ664543 Croatian
U2e1b
KJ856692 Russia East.EU
possibly Inner Asia
JQ705941.1 Czech
KJ856743.1 Inner Mongolia China
Karos3/7
Karos3/8
Karos3/9
U2e
AY714049.1 South Asia
KJ856830 Russian
Karos1/12
KJ856725 Belarus
JQ702663 no data
JX462739.1 India
KJ856833 Ukraine
JQ705384 Finland
I0012, I0017 Holocen, Sweden
JQ703917 Poland
U2e1b1
Europe
U2e1b2

**Network 43.**

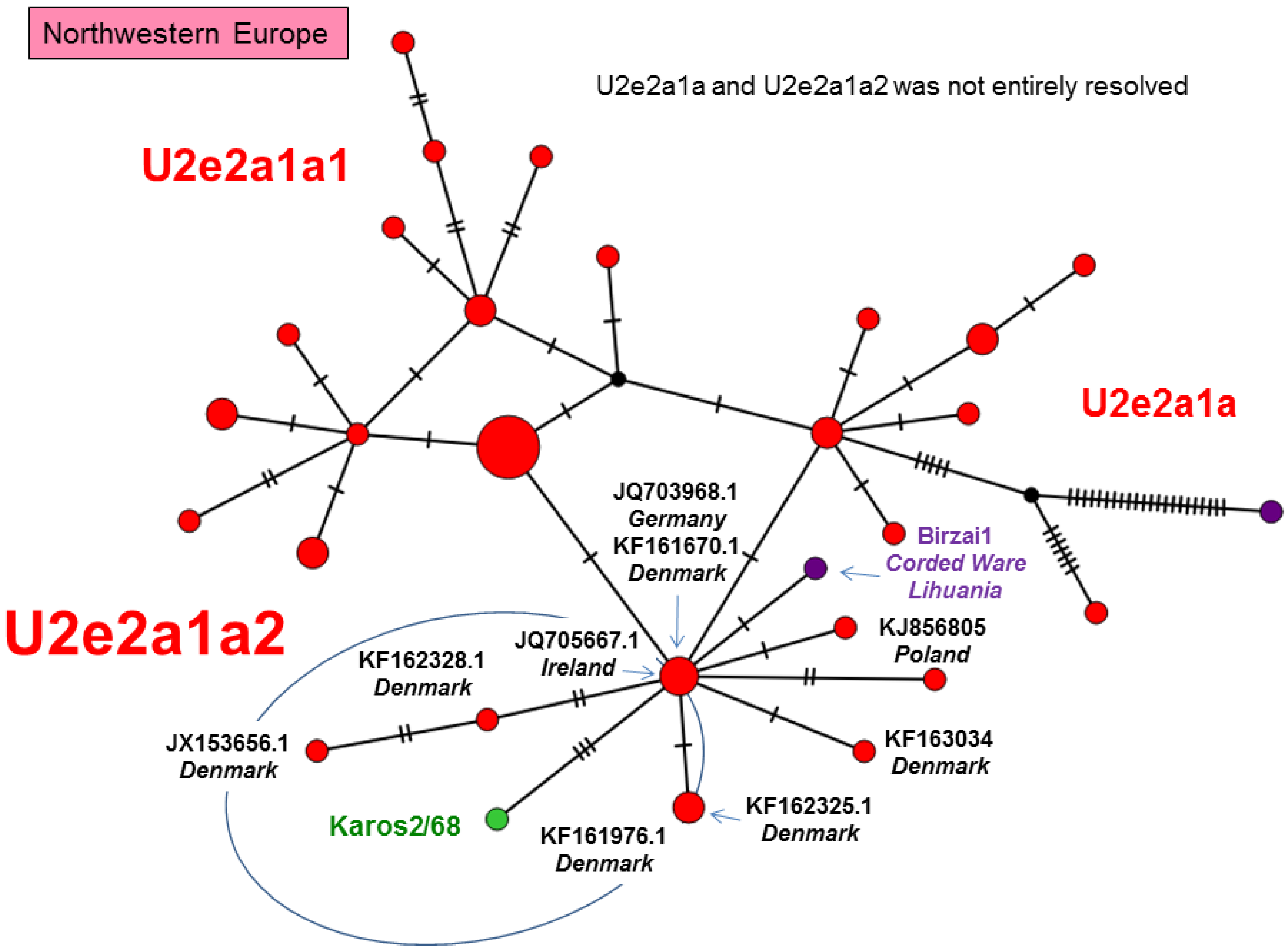

Northwestern Europe
U2e2a1a and U2e2a1a2 was not entirely resolved
U2e2a1a1
U2e2a1a
U2e2a1a2
JQ703968.1
Germany
KF161670.1
Denmark
Birzai1
Corded Ware
Lihuania
KJ856805
Poland
JQ705667.1
Ireland
KF162328.1
Denmark
JX153656.1
Denmark
KF163034
Denmark
KF162325.1
Denmark
Karos2/68
KF161976.1
Denmark


**Network 44.**

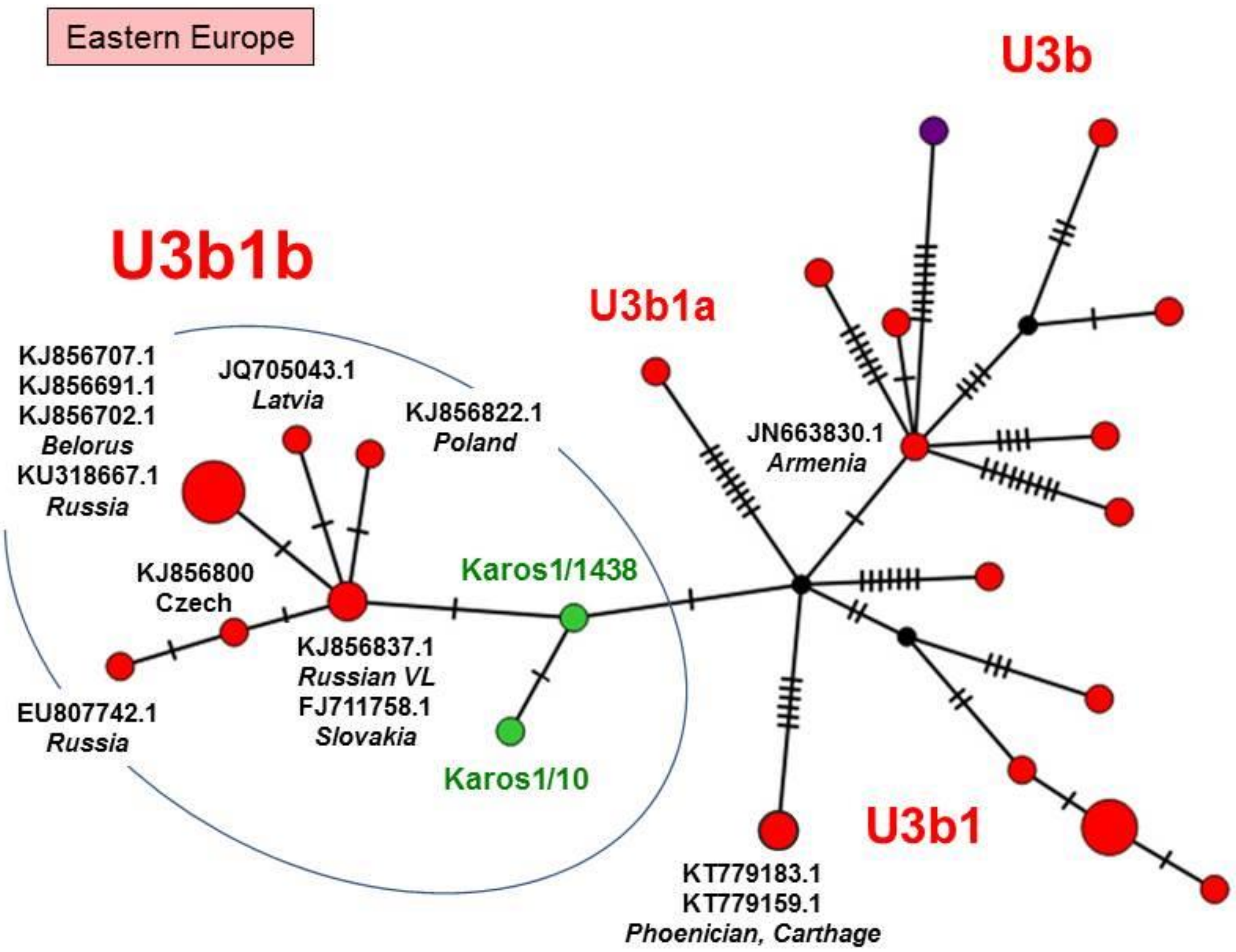
Eastern Europe
U3b
U3b1b
U3b1a
KJ856707.1
KJ856691.1
KJ856702.1
Belorus
KU318667.1
Russia
JQ705043.1
Latvia
KJ856822.1
Poland
JN663830.1
Armenia
KJ856800
Czech
Karos1/1438
KJ856837.1
Russian VL
FJ711758.1
Slovakia
EU807742.1
Russia
Karos1/10
U3b1
KT779183.1
KT779159.1
Phoenician, Carthage

**Network 45.**

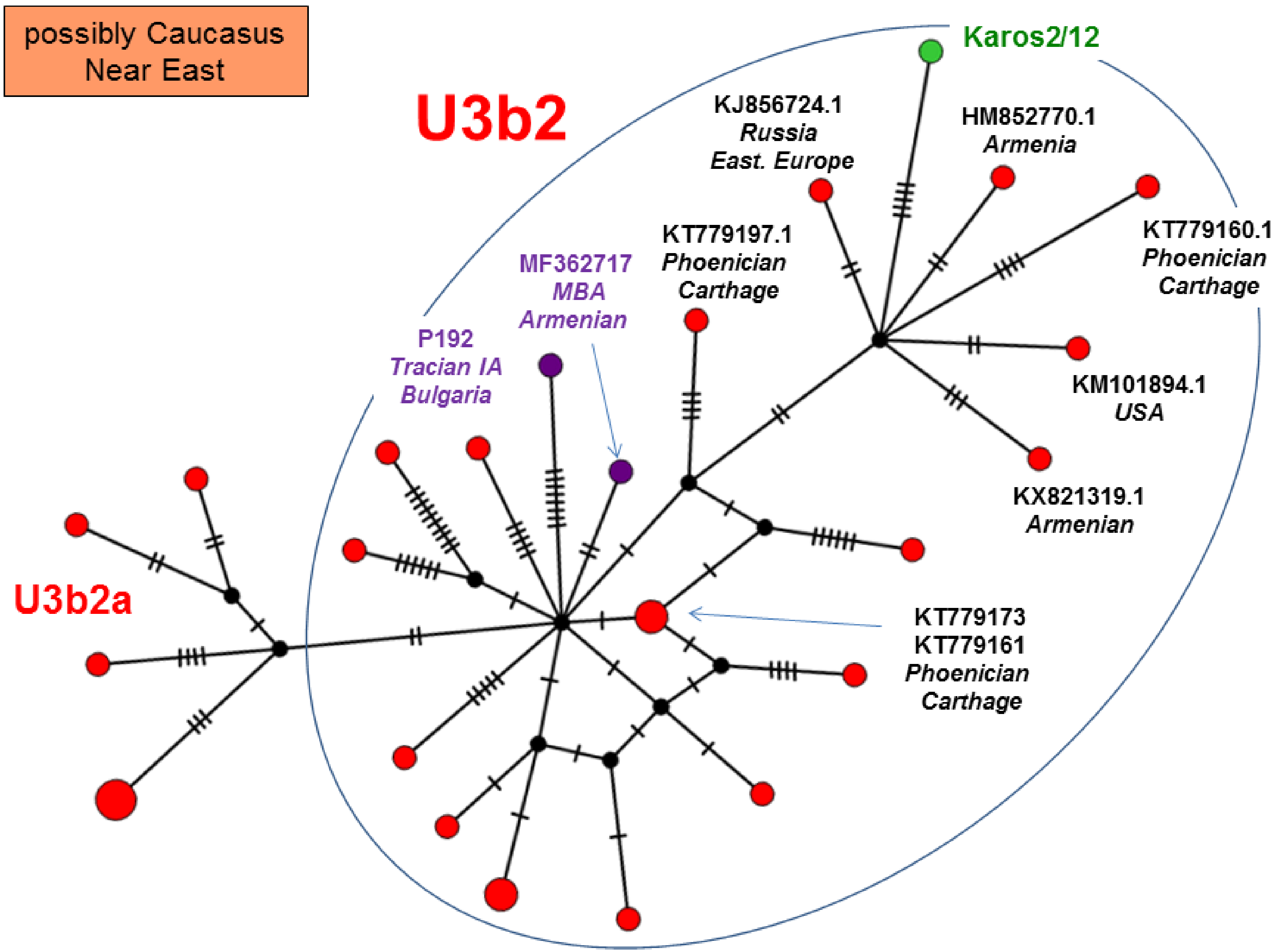
possibly Caucasus
Near East
U3b2
Karos2/12
KJ856724.1
Russia
East. Europe
HM852770.1
Armenia
KT779197.1
Phoenician
Carthage
KT779160.1
Phoenician
Carthage
MF362717
MBA
Armenian
P192
Tracian IA
Bulgaria
KM101894.1
USA
KX821319.1
Armenian
U3b2a
KT779173
KT779161
Phoenician
Carthage

**Network 46.**

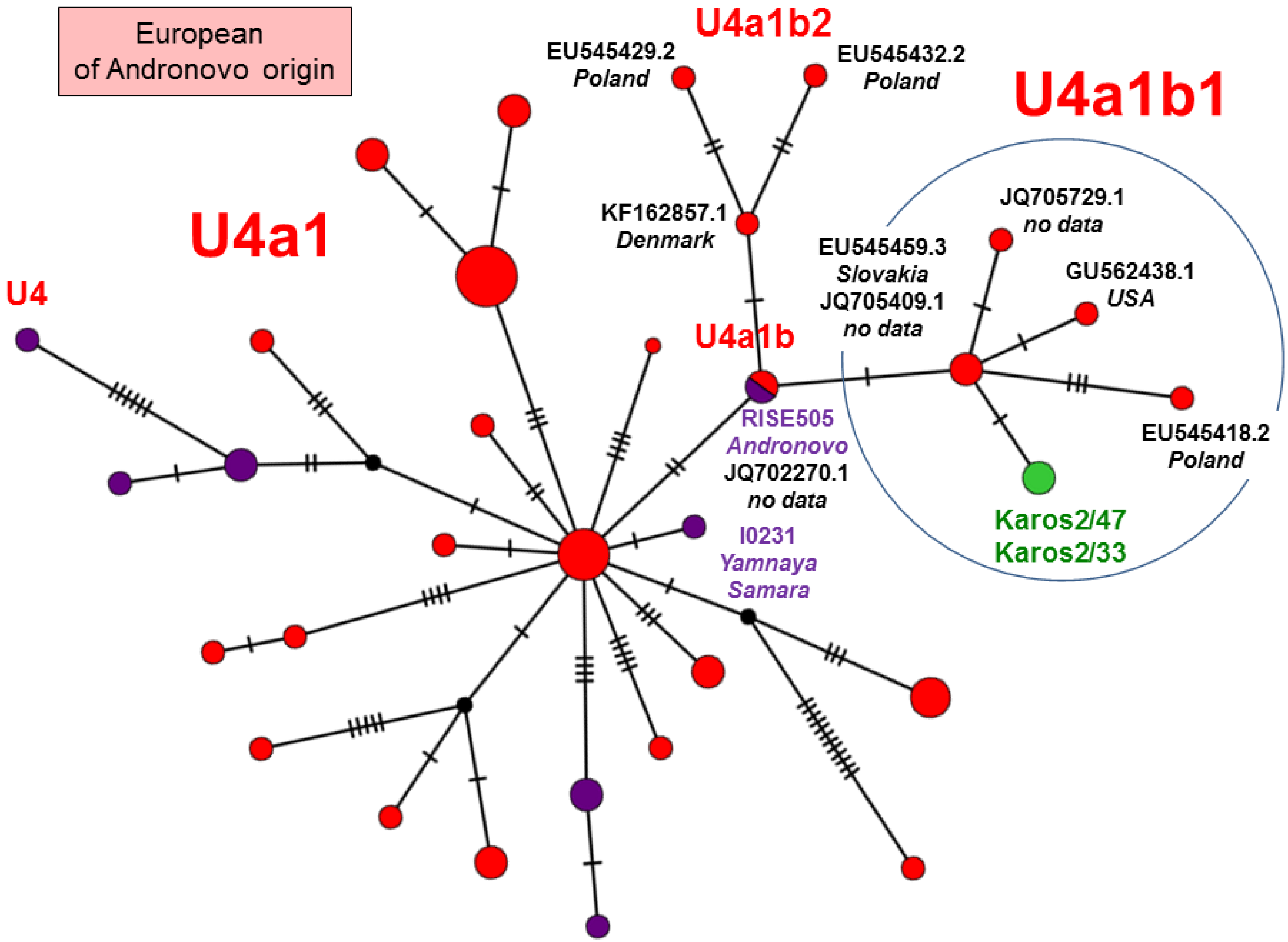
European
of Andronovo origin
U4a1b2
EU545429.2
Poland
EU545432.2
Poland
U4a1b1
U4a1
U4
KF162857.1
Denmark
JQ705729.1
no data
EU545459.3
Slovakia
JQ705409.1
no data
GU562438.1
USA
U4a1b
RISE505
Andronovo
JQ702270.1
no data
EU545418.2
Poland
I0231
Yamnaya
Samara
Karos2/47
Karos2/33

**Network 47.**

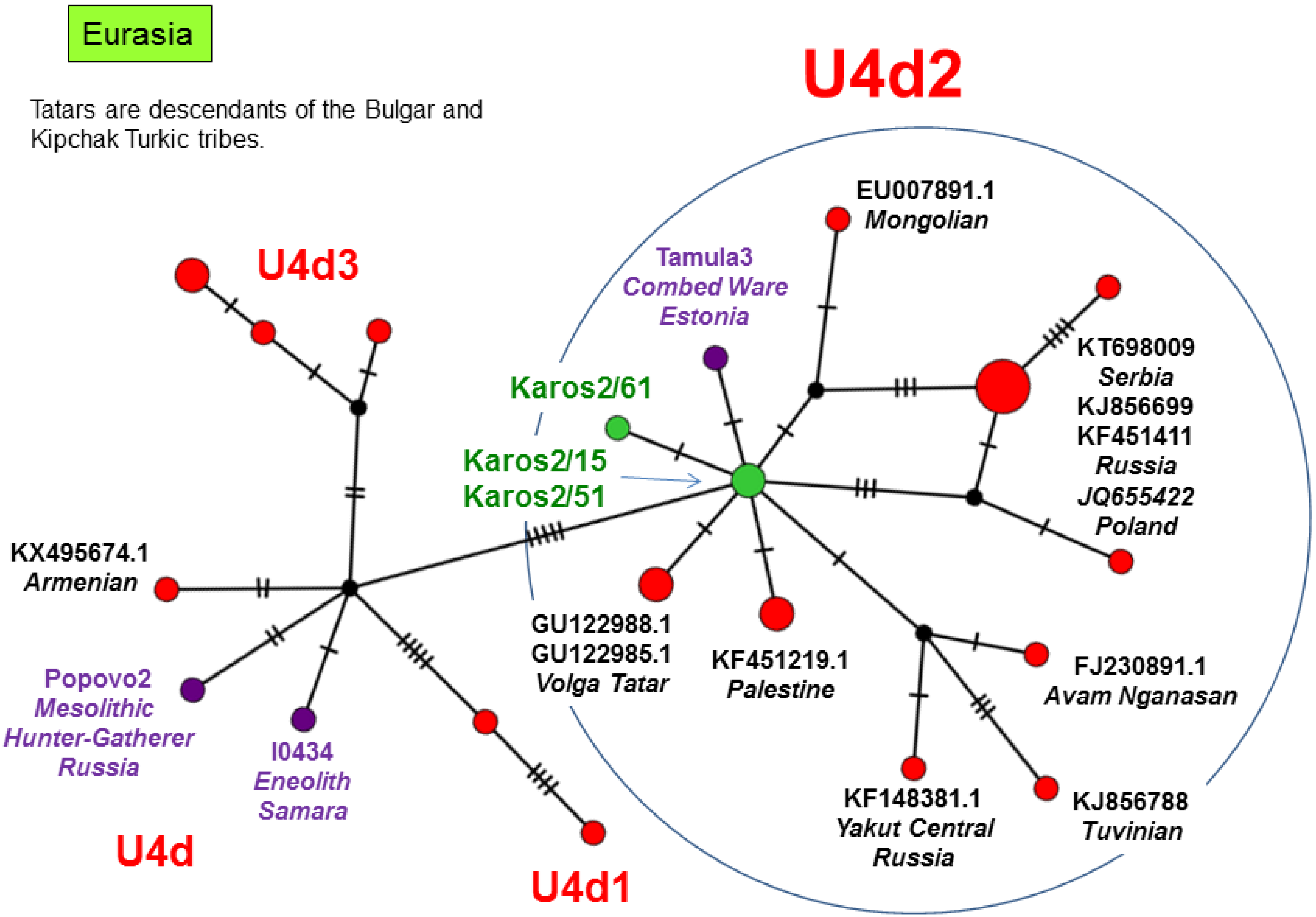
Eurasia
Tatars are descendants of the Bulgar and Kipchak Turkic tribes.
U4d2
EU007891.1
Mongolian
U4d3
Tamula3
Combed Ware
Estonia
KT698009
Serbia
KJ856699
KF451411
Russia
JQ655422
Poland
Karos2/61
Karos2/15
Karos2/51
KX495674.1
Armenian
GU122988.1
GU122985.1
Volga Tatar
KF451219.1
Palestine
FJ230891.1
Avam Nganasan
Popovo2
Mesolithic
Hunter-Gatherer
Russia
I0434
Eneolith
Samara
KF148381.1
Yakut Central
Russia
KJ856788
Tuvinian
U4d
U4d1

**Network 48.**

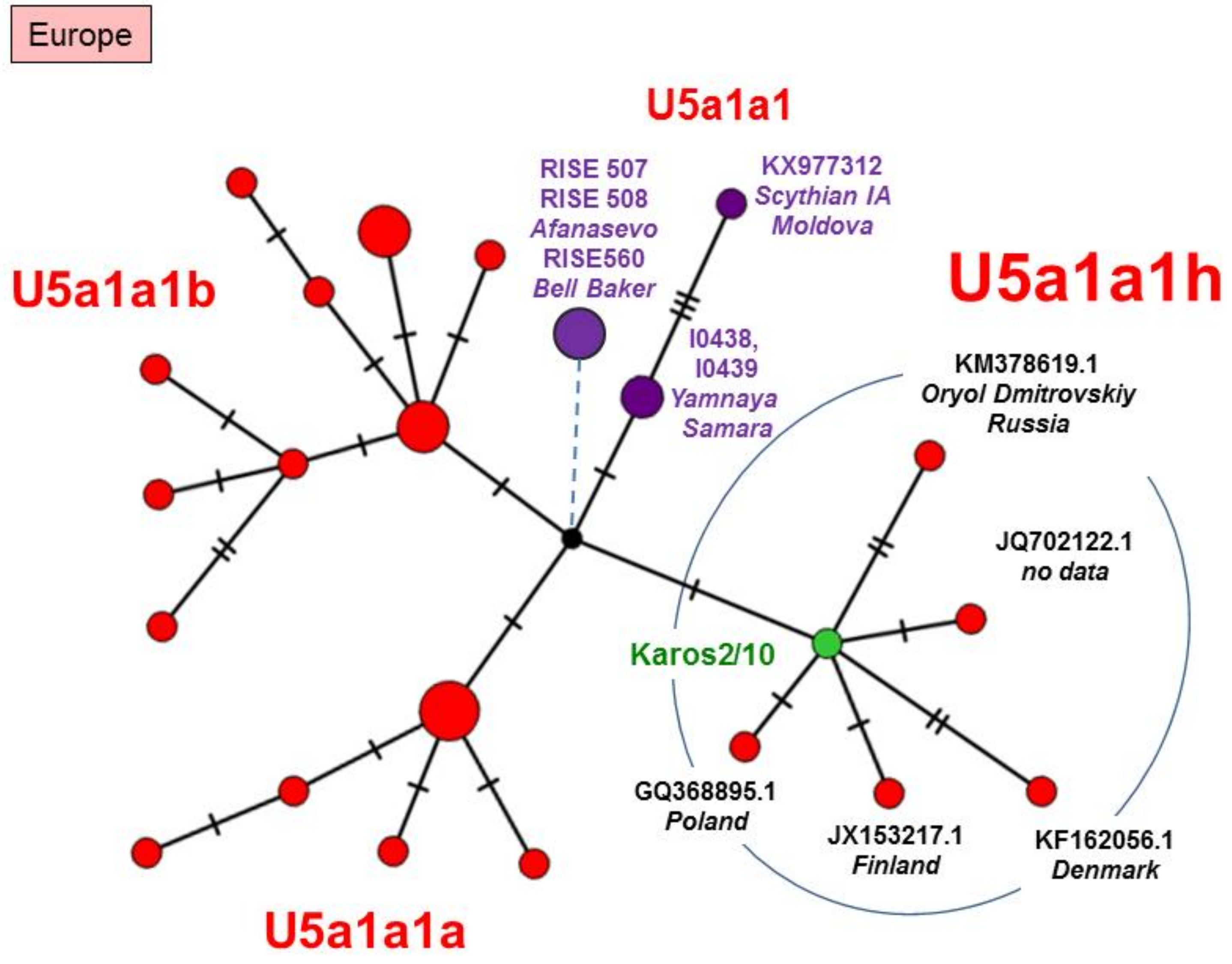
Europe
U5a1a1
RISE 507
RISE 508
Afanasevo
RISE560
Bell Baker
KX977312
Scythian IA
Moldova
U5a1a1b
U5a1a1h
I0438,
I0439
Yamnaya
Samara
KM378619.1
Oryol Dmitrovskiy
Russia
JQ702122.1
no data
Karos2/10
GQ368895.1
Poland
JX153217.1
Finland
KF162056.1
Denmark
U5a1a1a

**Network 49.**

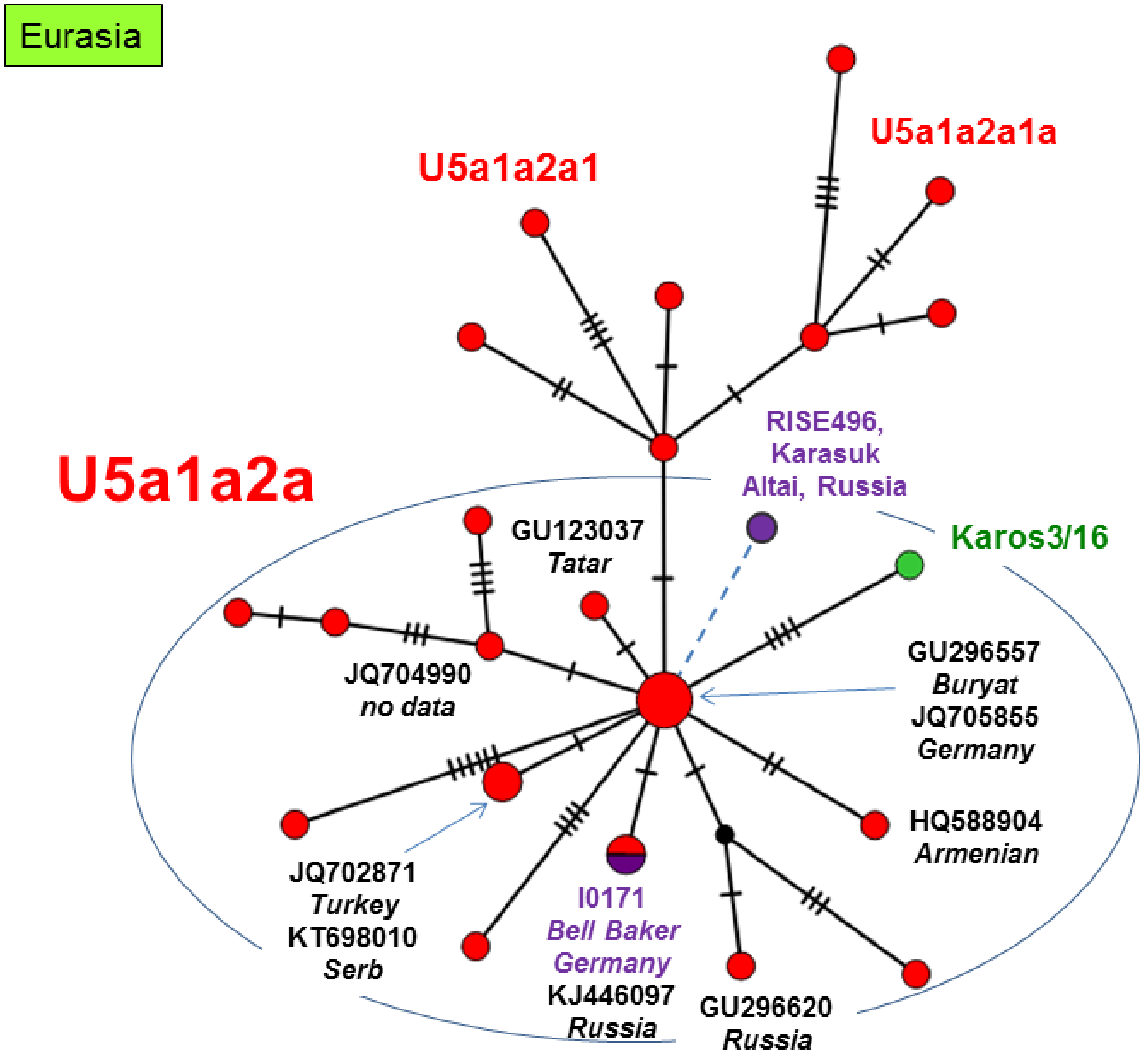
Eurasia
U5a1a2a1a
U5a1a2a1
U5a1a2a
RISE496,
Karasuk
Altai, Russia
Karos3/16
GU123037
Tatar
JQ704990
no data
GU296557
Buryat
JQ705855
Germany
HQ588904
Armenian
JQ702871
Turkey
KT698010
Serb
I0171
Bell Baker
Germany
KJ446097
Russia
GU296620
Russia

**Network 50.**

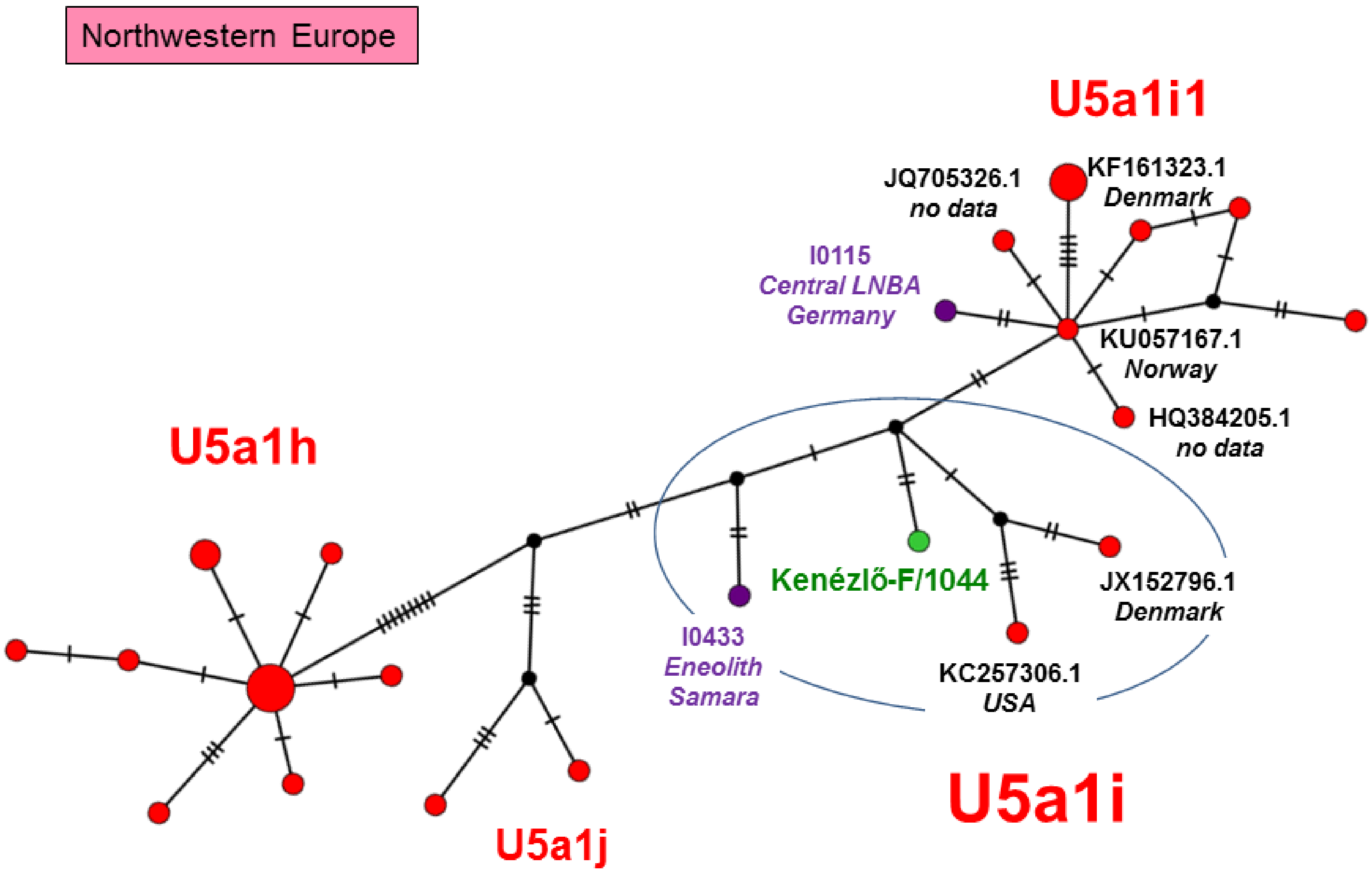
Northwestern Europe
U5a1i1
JQ705326.1
no data
KF161323.1
Denmark
I0115
Central LNBA
Germany
KU057167.1
Norway
HQ384205.1
no data
U5a1h
Kenézlő-F/1044
JX152796.1
Denmark
I0433
Eneolith
Samara
KC257306.1
USA
U5a1i
U5a1j

**Network 51.**

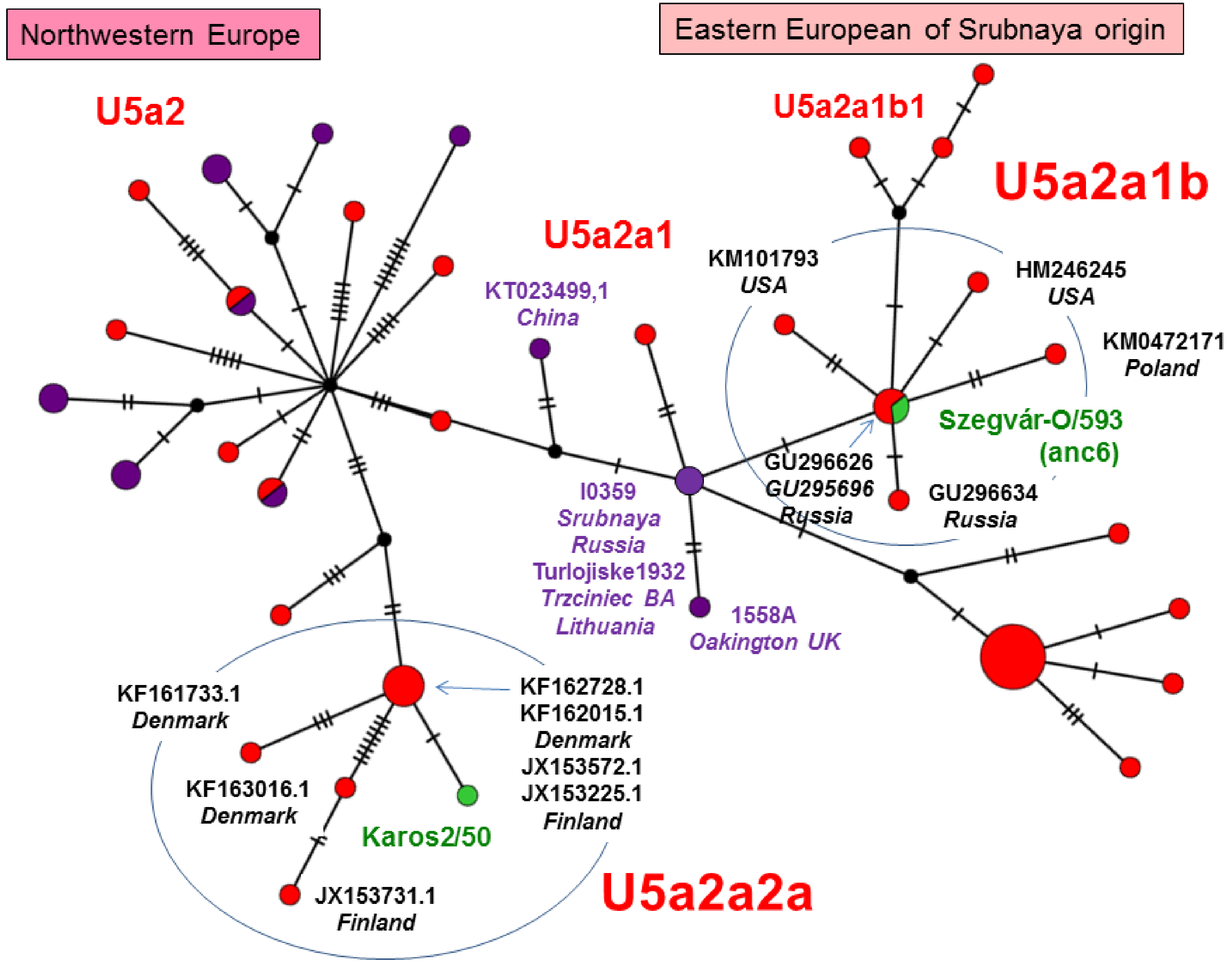
Northwestern Europe
Eastern European of Srubnaya origin
U5a2
U5a2a1b1
U5a2a1b
U5a2a1
KT023499,1
China
KM101793
USA
HM246245
USA
KM0472171
Poland
Szegvár-O/593
(anc6)
GU296626
GU295696
Russia
GU296634
Russia
I0359
Srubnaya
Russia
Turlojiske1932
Trzciniec BA
Lithuania
1558A
Oakington UK
KF161733.1
Denmark
KF162728.1
KF162015.1
Denmark
JX153572.1
JX153225.1
Finland
KF163016.1
Denmark
Karos2/50
JX153731.1
Finland
U5a2a2a

**Network 52.**

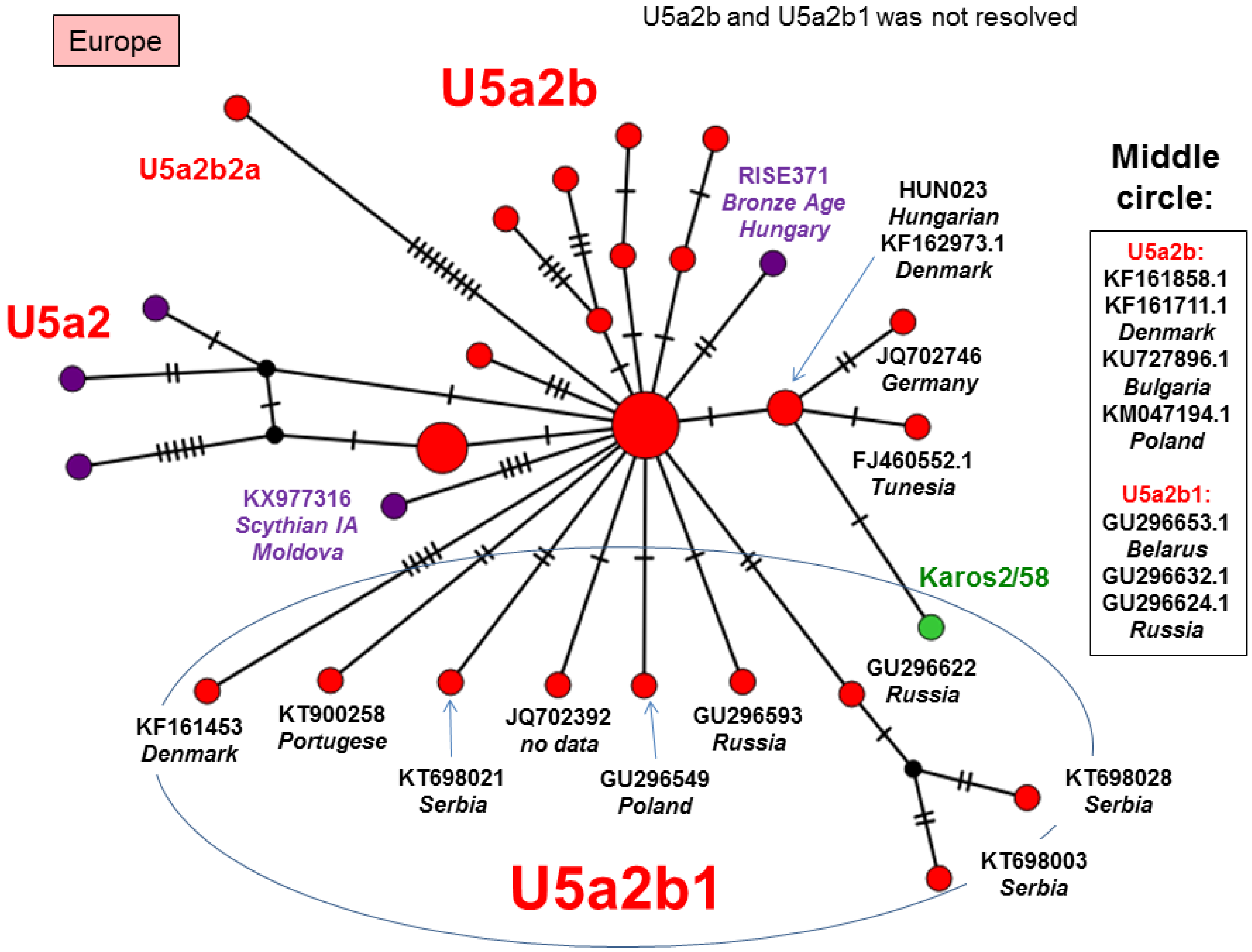

Europe
U5a2b and U5a2b1 was not resolved
U5a2b
U5a2b2a
U5a2
RISE371
Bronze Age
Hungary
HUN023
Hungarian
KF162973.1
Denmark
Middle circle:
U5a2b:
KF161858.1
KF161711.1
Denmark
KU727896.1
Bulgaria
KM047194.1
Poland
U5a2b1:
GU296653.1
Belarus
GU296632.1
GU296624.1
Russia
JQ702746
Germany
FJ460552.1
Tunesia
KX977316
Scythian IA
Moldova
Karos2/58
GU296622
Russia
KF161453
Denmark
KT900258
Portugese
KT698021
Serbia
JQ702392
no data
GU296549
Poland
GU296593
Russia
KT698028
Serbia
KT698003
Serbia
U5a2b1


**Network 53.**

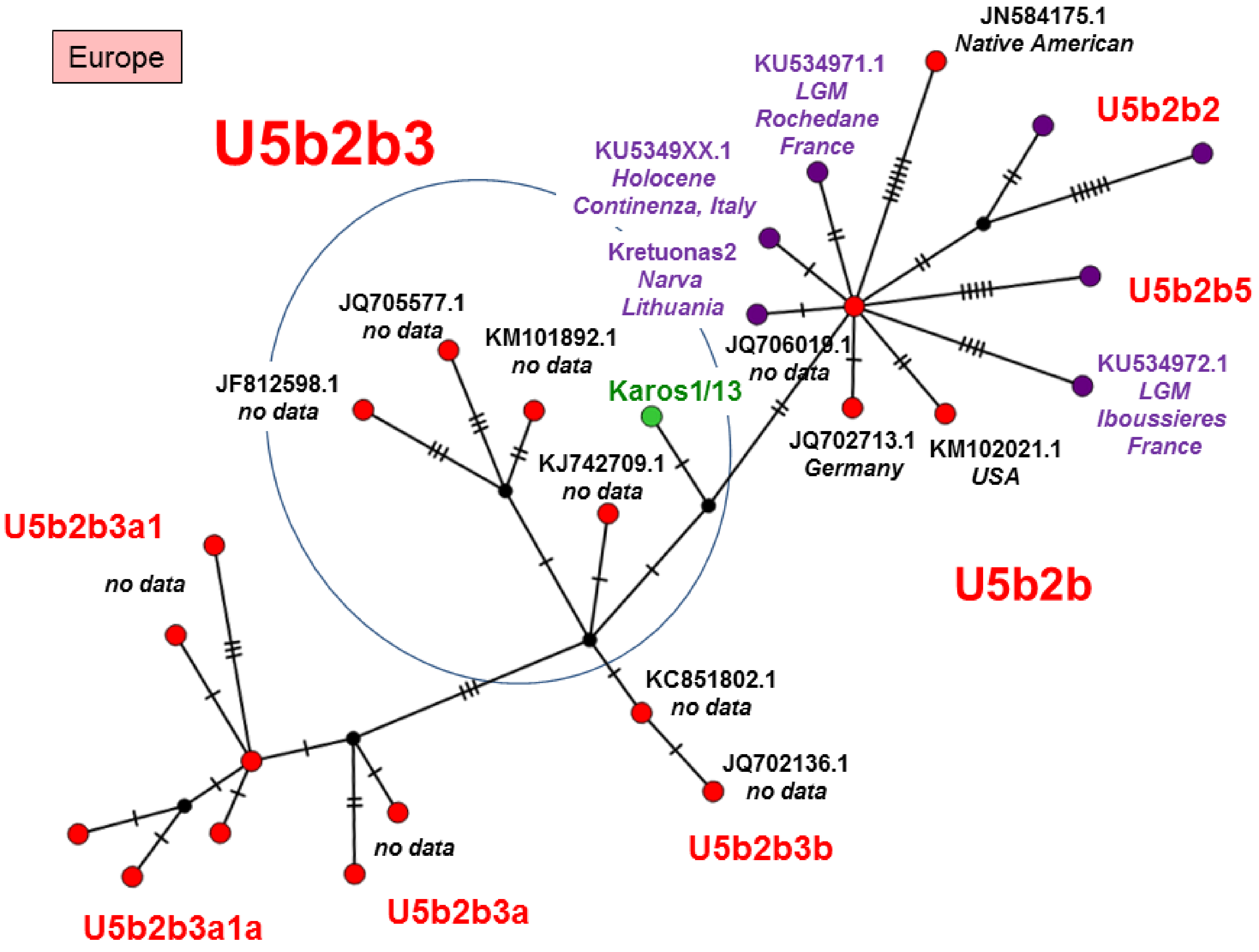
Europe
U5b2b3
JN584175.1
Native American
KU534971.1
LGM
Rochedane
France
U5b2b2
KU5349XX.1
Holocene
Continenza, Italy
Kretuonas2
Narva
Lithuania
U5b2b5
JQ705577.1
no data
KM101892.1
no data
JF812598.1
no data
Karos1/13
JQ706019.1
no data
KU534972.1
LGM
Ibousslieres
France
JQ702713.1
Germany
KM102021.1
USA
KJ742709.1
no data
U5b2b3a1
no data
U5b2b
KC851802.1
no data
JQ702136.1
no data
no data
U5b2b3b
U5b2b3a1a
U5b2b3a

Network 54.

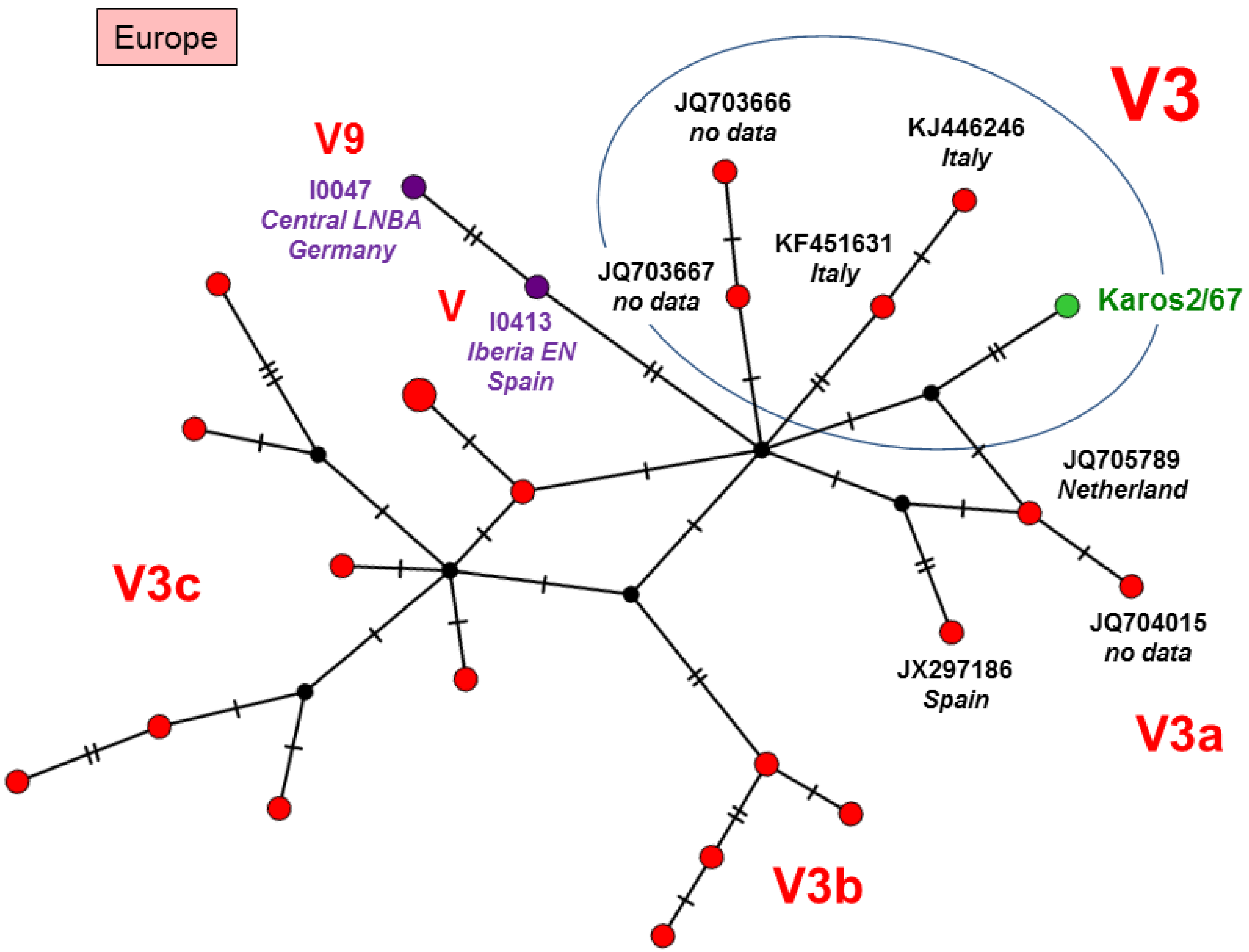
Europe
V3
V9
I0047
Central LNBA
Germany
V
I0413
Iberia EN
Spain
JQ703666
no data
KJ446246
Italy
KF451631
Italy
JQ703667
no data
Karos2/67
JQ705789
Netherland
JQ704015
no data
JX297186
Spain
V3c
V3a
V3b

**Network 55.**

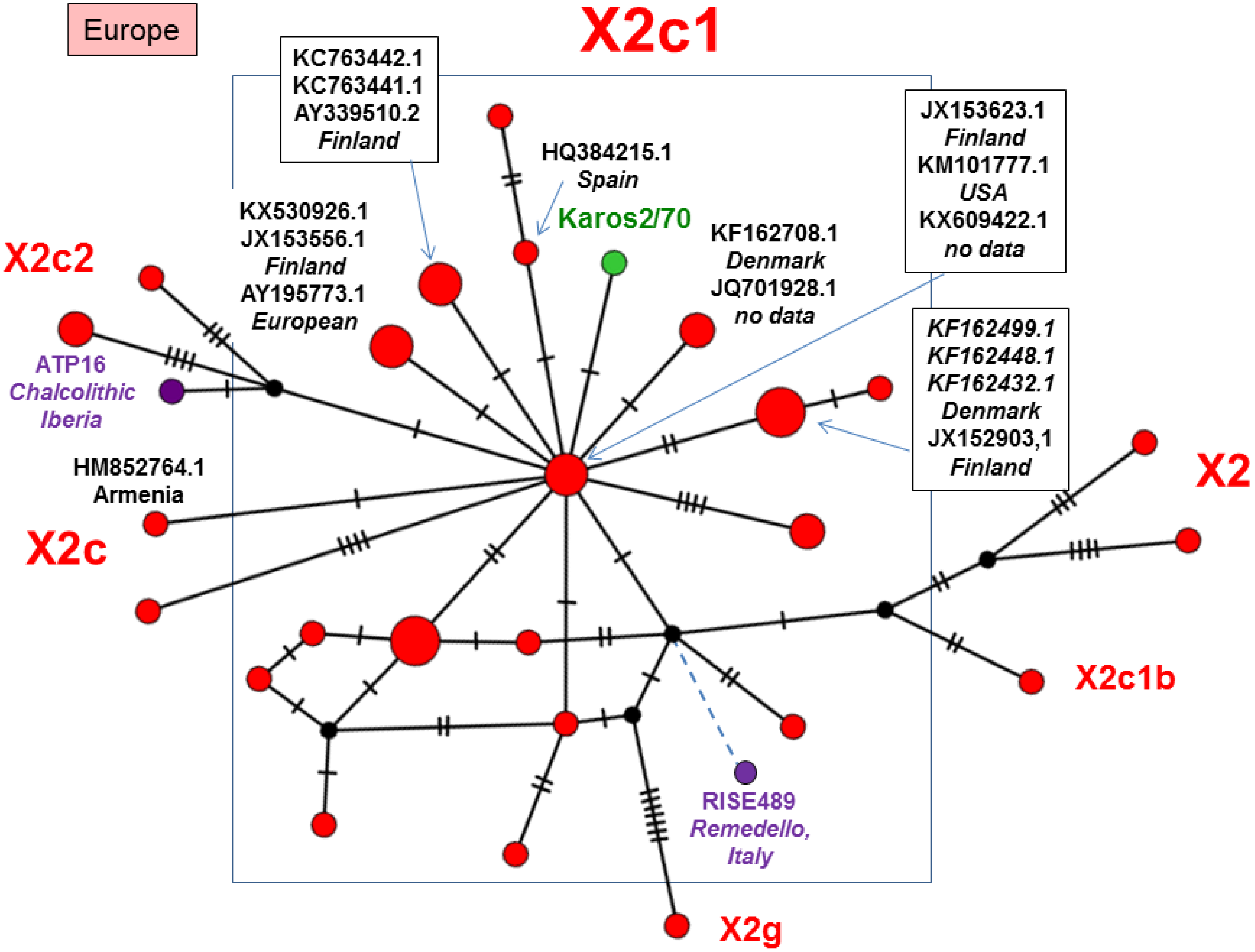
Europe
X2c1
KC763442.1
KC763441.1
AY339510.2
Finland
HQ384215.1
Spain
Karos2/70
JX153623.1
Finland
KM101777.1
USA
KX609422.1
no data
KX530926.1
JX153556.1
Finland
AY195773.1
European
KF162708.1
Denmark
JQ701928.1
no data
X2c2
ATP16
Chalcolithic
Iberia
KF162499.1
KF162448.1
KF162432.1
Denmark
JX152903,1
Finland
HM852764.1
Armenia
X2
X2c
X2c1b
RISE489
Remedello,
Italy
X2g

**Network 56.**

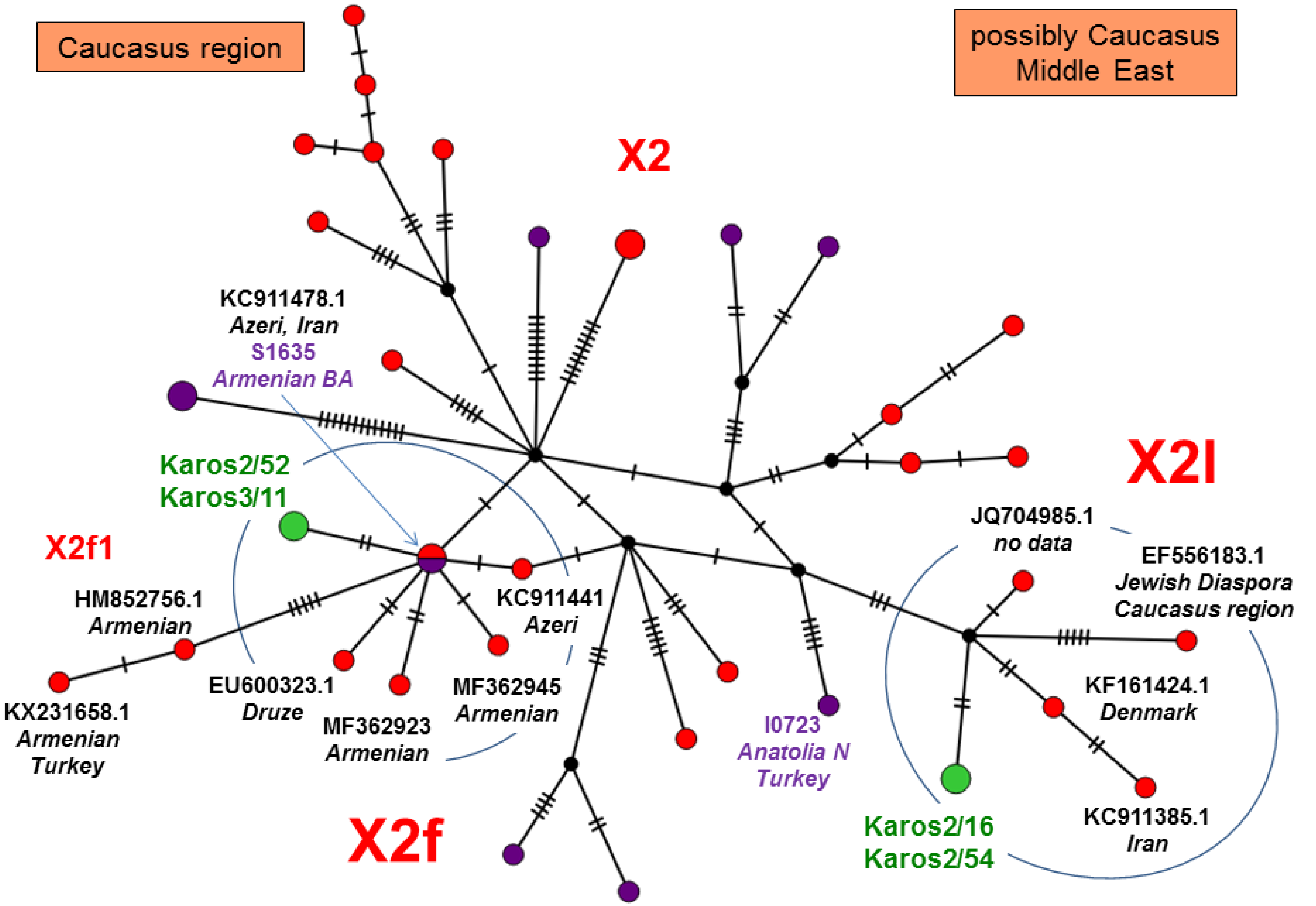

Caucasus region
possibly Caucasus Middle East
X2
X2l
X2f1
X2f
KC911478.1
Azeri, Iran
S1635
Armenian BA
Karos2/52
Karos3/11
HM852756.1
Armenian
KX231658.1
Armenian
Turkey
EU600323.1
Druze
MF362923
Armenian
MF362945
Armenian
KC911441
Azeri
I0723
Anatolia N
Turkey
JQ704985.1
no data
EF556183.1
Jewish Diaspora
Caucasus region
KF161424.1
Denmark
KC911385.1
Iran
Karos2/16
Karos2/54


**Network 57.**

East Asia

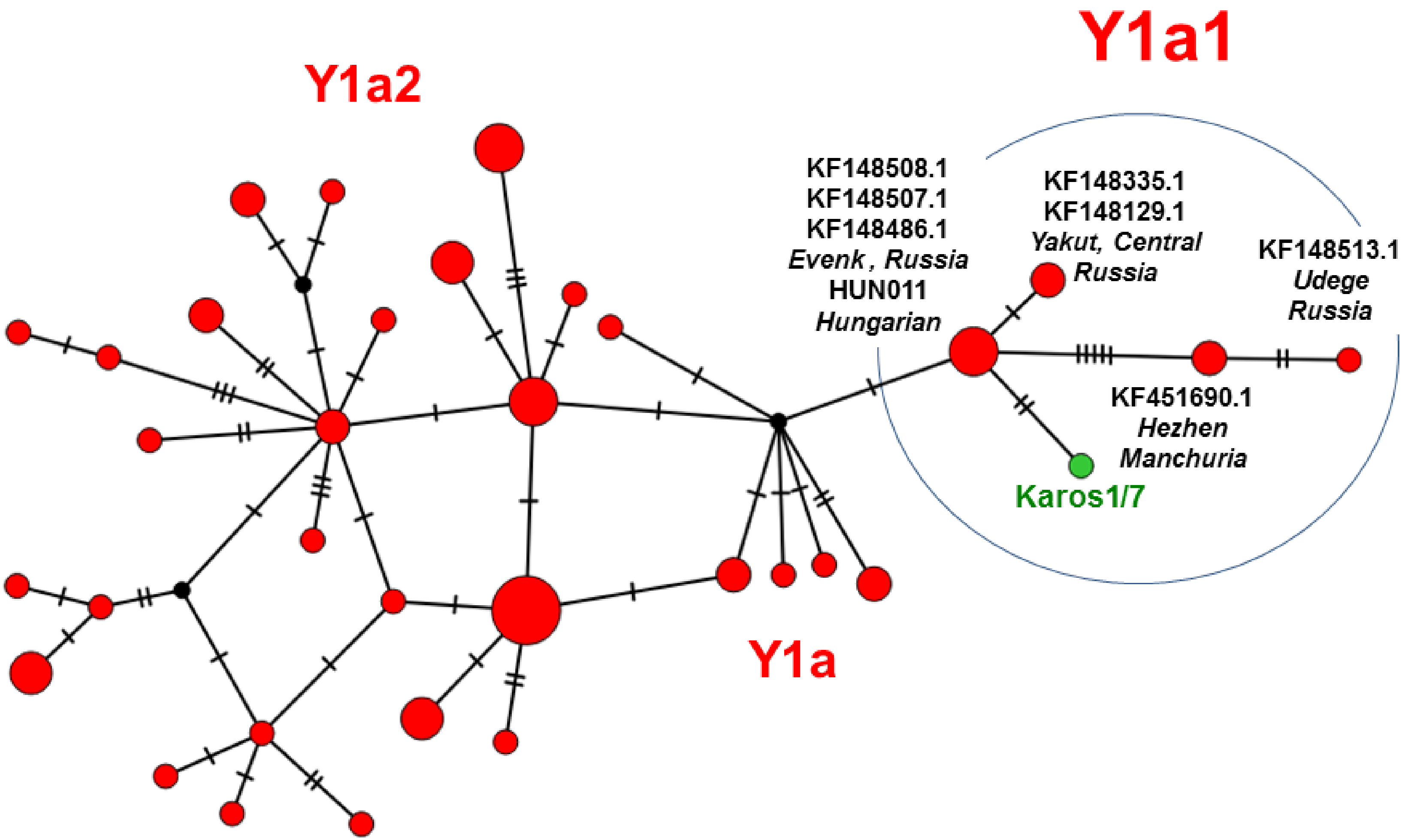


**Network 58.**
